# Gaze patterns during visual mental imagery reflect part-based generation

**DOI:** 10.1038/s41598-026-35447-z

**Published:** 2026-01-13

**Authors:** Enea J. Weber, Fred W. Mast

**Affiliations:** Department of Psychology, University of Bern, 3012 Bern, Switzerland

**Keywords:** Mental imagery, Eye-tracking, Gaze-contingent window, Psychology, Human behaviour

## Abstract

Eye movements during visual mental imagery resemble those made during prior perception. Across two experiments, we investigated whether eye movements during imagery reflect a part-by-part generation of mental images, by comparing gaze patterns during mental imagery to those during part-based viewing (using a gaze-contingent window, GCW) and to those during holistic viewing (using an artificial scotoma, AS). In Experiment 1, participants freely encoded and imagined pictures before reinspecting them either part-by-part (GCW condition), or holistically (AS condition). The results show that fixation scanpaths (MultiMatch) and refixation patterns (recurrence quantification analysis) during mental imagery largely mirror those during GCW viewing. In Experiment 2, we examined whether this effect depends on prior perceptual encoding. Pictures were initially encoded either freely, with the AS, or with the GCW, and subsequently imagined. The results show that regardless of how the pictures were initially encoded, gaze patterns during mental imagery systematically resembled part-based perception. The current study provides direct evidence that eye movements during mental imagery reflect a part-by-part generation process of the imagined content, independent of prior perceptual encoding.

## Introduction

Seeing and imagining feel different, but our eyes tell a strikingly similar story. When we imagine a visual scene, eye fixations tend to return to the same locations visited during perceptual encoding. This phenomenon is known as the “Looking at Nothing” (LAN) effect^1–4^. Previous studies show that restricting eye movements when recalling a scene impairs memory performance^5–9^, suggesting that eye fixations help generate and maintain mental images^1,5,7,10^. It has been proposed that spatial indices tied to specific fixations are automatically stored in memory^3,11,12^. During subsequent mental imagery of the scene, the eyes return to these spatial indices and thereby help to reactivate visual information stored in memory and to arrange the different parts in their corresponding spatial location^5,6,13^.

The resemblance of eye movements during visual mental imagery and perceptual encoding is in line with concepts of mental imagery as a simulation of perception^14,15^ or as “vision in reverse”^16^. This view is supported by both neuroimaging^17,18^ and behavioral research^19,20^ showing substantial overlap between imagery and perception. However, clinical cases show that the two processes can dissociate, with selective impairments in visual imagery^21^ or in perception^22,23^.

Several studies suggested that oculomotor patterns are encoded alongside visual information and later reinstated during imagery^6,24,25^. However, there is growing evidence that eye fixations during imagery are not replayed from perceptual encoding. Previous studies showed that even when participants had to keep central fixation during encoding, they still made spread out fixations during subsequent imagery^5,11^. Yet another study showed that participants made spontaneous eye movements toward the respective side of an imagined map of France in response to verbal cues, despite not having seen the map during the experiment^26^. Moreover, fine-grained analyses in fixation scanpaths using the MultiMatch algorithm (which compares scanpaths along multiple dimensions) show less similarity between imagery and perception^27^, and the analysis of temporal gaze patterns reveal substantial differences between mental imagery and perception^28,29^. Recurrence quantification analysis (RQA) shows more frequent refixations during imagery, and these refixations follow a sequential order that is less pronounced during perceptual encoding^27–29^. The temporal patterns suggest a reactivation of place-bound pictorial content during imagery^29^, providing further evidence that eye movements during imagery are not simply replayed from perception.

During perceptual encoding, the visual system creates a coherent representation of the scene by integrating information part-by-part and at the same time forming a holistic representation of the scene^30^. Unlike percepts, mental images are generated entirely from memory and are likely constructed part-by-part^31^. Spatial indexes serve to guide the eyes, and assemble from memory different parts of the mental image^5,6,10,13^. In the same vein, consistent refixations to specific parts reactivate visual representations, preventing the mental image from fading^28,29^. Thus, eye movements during imagery can resemble those during perception not necessarily because they are replayed, but because they rely on part-based processes.

In the present study, we manipulate the way participants inspect scenes and compare the resulting eye movements to those during visual mental imagery. Specifically, we compared eye movements during imagery to those observed under viewing conditions that promote either part-based (gaze-contingent window, GCW) or holistic perceptual encoding (artificial scotoma, AS). By means of a GCW, participants see only a small circular region of the locations around their fixation (similar to tunnel vision), leading them to gather information in discrete chunks^32–34^. This encourages participants to create a coherent percept from isolated parts. When using an artificial scotoma (AS), participants lose central vision but retain peripheral visual input. As a result, they rely on peripheral vision to encode the general gist of the scene without focusing on individual details. This encourages a more holistic encoding strategy^33–35^. Prior work has demonstrated that holistic and part-based encoding give rise to distinct scanpaths^36,37^. If mental imagery involves assembling a scene from individual parts, spatial and temporal gaze patterns should resemble those observed under GCW viewing. If, however, eye movements during AS viewing turn out to be similar to those during mental imagery, this would imply a holistic representation. Finally, if gaze patterns during imagery resemble those from free viewing (as suggested by a replay of perception), this would suggest the involvement of holistic and part-based processes.

In Experiment 1, pictures were always encoded freely prior to the imagery phase. This is the usual procedure for research in mental imagery. In order to investigate the role of encoding, we designed Experiment 2, in which we varied how participants viewed the pictures for the first time (freely, holistically, or part-by-part). If mental imagery involves a part-based construction process, we expected spatiotemporal gaze patterns during imagery to resemble those observed during GCW viewing, independent of the type of encoding.

## Results

### Experiment 1

**Fig. 1:**
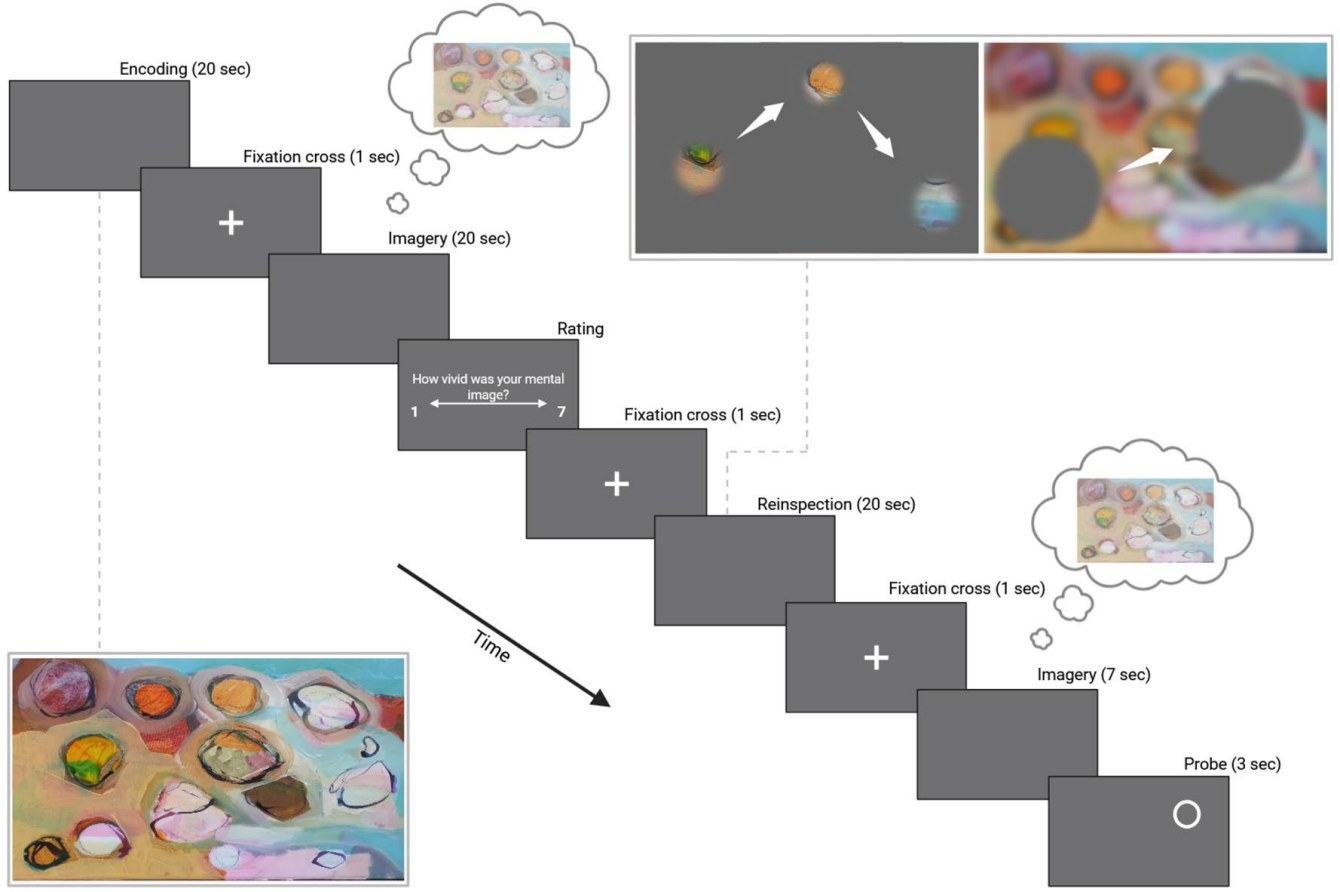
Procedure for Experiment 1. First, participants freely encoded pictures from three categories (art, indoor, and outdoor scenes) for 20 s, before a 1 sec fixation cross. Afterwards, they were instructed to imagine the content of the picture as vividly as possible for 20 s, and they had to rate the vividness of their mental image on a scale from 1 to 7. Subsequently, another fixation cross (1 s) appeared and participants saw the initial picture again with either a gaze-contingent window (GCW) or an artificial scotoma (AS) for 20 s. After the reinspection, participants saw a fixation cross (1 s) and imagined the picture again for 10 sec. A probe appeared within the 3 last seconds, followed by a question about whether a specific object or color fell under it. The first encoding was without any constraints, and the reinspection occurred either by means of a GCW (left panel) or by means of an AS (right panel). In the AS illustration, the image is blurred to illustrate that participants had access only to peripheral vision.

In Experiment 1, we investigate spatiotemporal similarities between eye movements during mental imagery compared to part-based and holistic perception. To ensure that eye movements during imagery were not influenced by encoding, participants first encoded the pictures freely, then imagined what they saw, and viewed the pictures again under GCW or AS conditions (see Figure 1). We used pictures from three categories (abstract art, landscapes and indoor scenes) to test how different contents affect eye movements.

#### AOI analysis

**Fig. 2:**
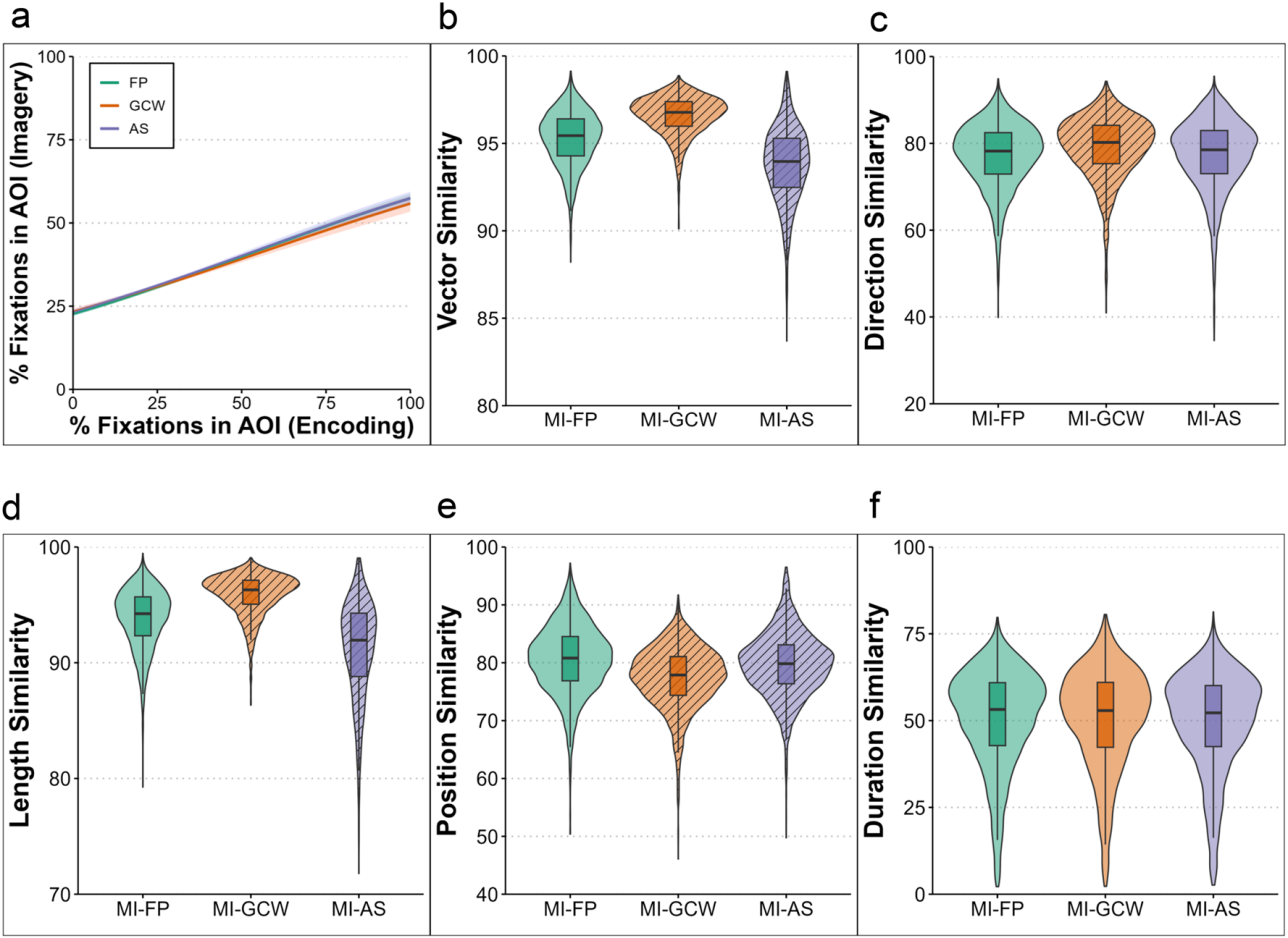
Spatial analyses. (**a**) shows the relationship between the percentage of fixations within a specific AOI (LAN effect) during perception (x-axis) and mental imagery (y-axis), for free perception (green), gaze-contingent window (orange), and the artificial scotoma (purple) encoding conditions. Solid lines represent predicted values (estimates of the Bayesian model), and shaded areas show the 95 percent credible intervals. Overall, the LAN effect is robust and similar across all conditions. All other panels show the MultiMatch similarity scores (y-axes) between mental imagery (MI) and the different encoding conditions (x-axes) for the different parameters. The violins display the distribution of the similarity scores for each participant and trial across the different comparisons. Striped violins indicate comparisons (MI-GCW or MI-AS) for which the Bayesian model estimated a credible difference from the MI-FP reference condition, with 95% credible intervals that did not include zero. The similarity between MI and GCW was higher compared to the other comparisons for the general vector shape (**b**), the length of saccades (**c**), and the direction of saccades (**d**). However, this was not the case for the similarity in the absolute position of the fixations (**e**) and for the fixation durations within the scanpaths (**f**).

We found a main effect of fixations across all types of encoding (estimate = 0.018, lower CI = 0.017, upper CI = 0.019) on fixations during mental imagery (MI), showing a clear LAN effect. The credible intervals of the interaction between fixations during encoding and the GCW (estimate = -0.001, lower CI = -0.003, upper CI = 0.001), and the AS condition (estimate = -0.000, lower CI = -0.002, upper CI = 0.001) included 0. Thus, the similarity in the spatial distribution was comparable between MI and all viewing conditions (see Fig. 2a).

#### Scanpaths

The vector (shape) similarity (Fig. 2b) was higher between MI and GCW (estimate = 0.349, lower CI = 0.323, upper CI = 0.375) and lower between MI and AS (estimate = -0.243, lower CI = -0.265, upper CI = -0.221) compared to the similarity between MI and FP. For the direction parameter (Fig. 2c), the similarity between MI and GCW was higher than with FP (estimate = 0.085, lower CI = 0.052, upper CI = 0.116). The similarity between MI and AS did not differ from the similarity with FP (estimate = -0.021, lower CI = -0.052, upper CI = 0.011). Thus, participants made saccades following similar directions between MI and part-based encoding. The similarity in length (Fig. 2d) was also higher for the MI-GCW comparison (estimate = 0.432, lower CI = 0.398, upper CI = 0.466) and lower for the AS-MI comparison (estimate = -0.312, lower CI = -0.340, upper CI = -0.284), compared to the MI-FP comparison, showing that the length of saccades during MI was most closely aligned with those during part-based encoding. The similarity score for position (Fig. 2e) was lower between MI-GCW (estimate = -0.258, lower CI = -0.285, upper CI = -0.230) and between MI-AS (estimate = -0.078, lower CI = -0.106, upper CI = -0.049) compared to MI-FP. Thus, the distance between the fixation locations within the scanpaths was smallest between MI and FP compared to the other encoding conditions. The credible intervals for the similarity in duration (Fig. 2f) of fixations between MI and GCW (estimate = -0.015, lower CI = -0.057, upper CI = 0.028) as well as MI and AS (estimate = -0.039, lower CI = -0.082, upper CI = 0.002) included 0, suggesting that both these comparisons did not differ from the comparison between MI and FP.

#### Temporal gaze dynamics

**Fig. 3:**
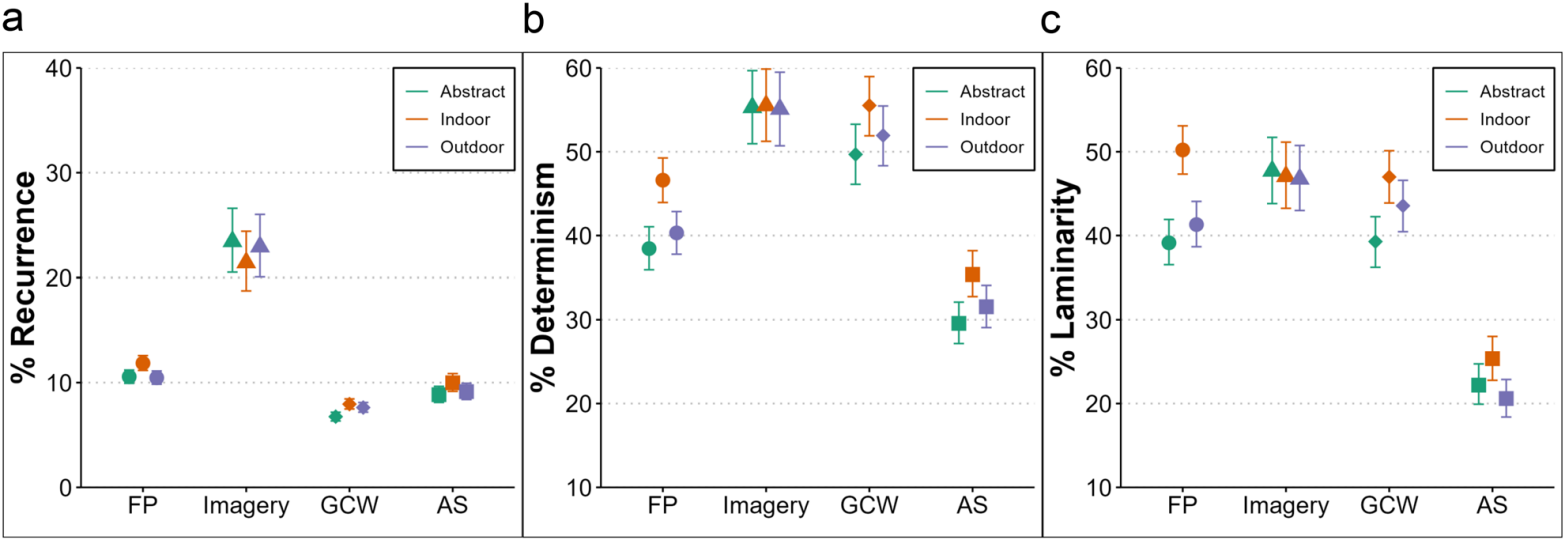
RQA results for Experiment 1. Recurrence (**a**), determinism (**b**), and laminarity (**c**) within each condition (x axis), for abstract art (green), indoor (orange), and outdoor (purple) picture categories. The shapes represent predicted values (estimates of the Bayesian model) while controlling for the fixation spread, and the bars represent the 95% credible intervals. Recurrence was higher during imagery, with more refixations (**a**), while determinism was greater in imagery and GCW encoding (**b**) but lower in AS. Laminarity was lower in AS (**c**), and both determinism and laminarity were higher for indoor scenes.

*Recurrence* was higher during MI compared to FP (estimate = 0.865, lower CI = 0.694, upper CI = 1.033), and lower during GCW (estimate = -0.490, lower CI = -0.535, upper CI = -0.443) and AS encoding (estimate = -0.231, lower CI = -0.308, upper CI = -0.157) compared to FP (Fig. 3a). Pictures of indoor scenes led to more recurrent fixations (estimate = 0.131, lower CI = 0.056, upper CI = 0.205) compared to abstract art pictures. Pictures from outdoor scenes did not differ from abstract pictures in terms of recurrent fixations (estimate = -0.011, lower CI = -0.086, upper CI = 0.065).

*Determinism* was higher during both MI (estimate = 0.730, lower CI = 0.552, upper CI = 0.912) and GCW encoding (estimate = 0.457, lower CI = 0.338, upper CI = 0.577) compared to FP, and lower in the AS compared to the FP condition (estimate = -0.376, lower CI = -0.478, upper CI = -0.273, Fig. 3b). Moreover, determinism was generally higher for indoor compared to abstract pictures (estimate = 0.335, lower CI = 0.259, upper CI = 0.410).

*Laminarity* was higher during MI compared to FP (estimate = 0.368, lower CI = 0.216, upper CI = 0.527). This indicates that during imagery, participants tend to cluster refixations within specific regions more frequently. The GCW did not lead to a change in laminarity compared to free perception (estimate = 0.015, lower CI = -0.080, upper CI = 0.113). The AS substantially decreased laminarity (estimate = -0.781, lower CI = -0.887, upper CI = -0.676), meaning that participants were less likely to make clustered refixations (Fig. 3c). Compared to abstract art, indoor scenes led to higher laminarity (estimate = 0.458, lower CI = 0.367, upper CI = 0.551), while laminarity for outdoor scenes did almost not differ from abstract art (estimate = 0.093, lower CI = 0.002, upper CI = 0.182).

#### Probe and vividness

Participants answered the questions following the probes with an overall accuracy of 70%. Accuracy was 70% for GCW trials and 69% for AS trials. Accuracy per category was 67% for abstract art, 77% for indoor scenes, and 65% for outdoor scenes.

The overall average vividness rating was 4.07 (on a 7-point scale). The average vividness rating was 4.13 for GCW trials and 4.02 for AS trials. Separately for each category, vividness ratings were 3.02 for abstract art, 4.55 for indoor scenes, and 4.65 for outdoor scenes.

#### Discussion

Experiment 1 shows that gaze patterns during mental imagery (MI) align with those during part-based viewing (GCW) and not with those during holistic viewing (AS) or free perception (FP). Specifically, MultiMatch results show that the overall shape of the fixation scanpaths during MI closely aligns with the scanpaths during GCW viewing, and differs most from those during AS viewing. The length and direction of saccades also show the highest similarity between MI and GCW viewing. A replay of eye movements during MI is not supported by these findings^24,38^. Instead, the results are in line with findings showing that fixations during MI are focused on specific parts that are important for mental reconstruction^7^. This aligns with the view that mental images are constructed by combining elements rather than retrieving the entire image holistically^39^. The similarity to part-based viewing supports the concept that different parts need to be reassembled to form a coherent mental scene^5,6,10^.

During encoding, visual exploration helps to construct robust memories. Eye movements assist in later recognition by focusing on specific parts to verify whether the scene matches a stored memory^40^. Similarly, in pattern completion tasks, gaze is directed to regions previously fixated during encoding that are most informative for recognizing an encoded image over a similar lure^41^. Thus, in the present study, it is likely that participants have selectively reinstated fixations to regions most relevant for constructing the mental image; similar to GCW viewing, where scanpaths had to be adapted to efficiently encode and assemble isolated parts into a coherent representation.

RQA results provide further support; recurrence was higher during imagery than during free perception, indicating that participants frequently returned to previously inspected locations. This is in line with previous studies indicating that refixations during imagery serve to refresh fragile internal representations^28,29^. Supporting this, fixations during imagery tend to favor low spatial frequencies^42^, suggesting that refixations help reactivate image regions that are represented with less detail. Importantly, determinism and laminarity were higher during both MI and GCW viewing, reflecting more clustered refixations occurring in the same sequential order. A prior study using GCW reported similar results^43^, and proposed that these gaze patterns reflect clustered refixations on specific regions for detailed inspection. Thus, high determinism and laminarity during imagery suggest a part-by-part construction of mental images rather than holistic retrieval. In line with this, both determinism and laminarity were higher for indoor scenes. Indoor scenes contain more objects, prompting systematic refixations to specific parts (higher determinism) and more clustered refixations for detailed inspection (higher laminarity)^43^. During imagery, stereotypical refixations can function as spatial anchors, linking visual details to specific locations. This allows retrieval by revisiting these areas in a consistent order, rather than relying on active maintenance in working memory^44^. Stereotypical refixations may also support the binding of spatial relations to a coherent representation. Refixations to previously occupied locations are known to facilitate such binding during sequential object encoding^45^. Furthermore, looking at empty screen regions during imagery has been proposed as a strategy to reduce cognitive load^13^. Therefore, repeated fixations in a consistent order might be the optimal strategy during imagery. In support of this, refixation sequences tend to follow systematic, sequential orders under high memory load^46^.

What determines which specific parts participants focus on during imagery? One possibility is that these parts were already prioritized during free encoding through covert attention shifts and were later revisited through overt fixations during imagery, ultimately resembling part-based viewing. Covert attention shifts have been shown to aid memory retrieval^47^. The GCW encourages overt attention to isolated parts, while the AS promotes a broader, global focus. Consequently, differences in initial exploration could lead to different gaze patterns during imagery. It is also possible that eye movements during mental imagery are independent of encoding. To investigate this, we conducted a second experiment that directly manipulates how pictures are initially encoded (freely, part-by-part, or holistically).

### Experiment 2

**Fig. 4:**
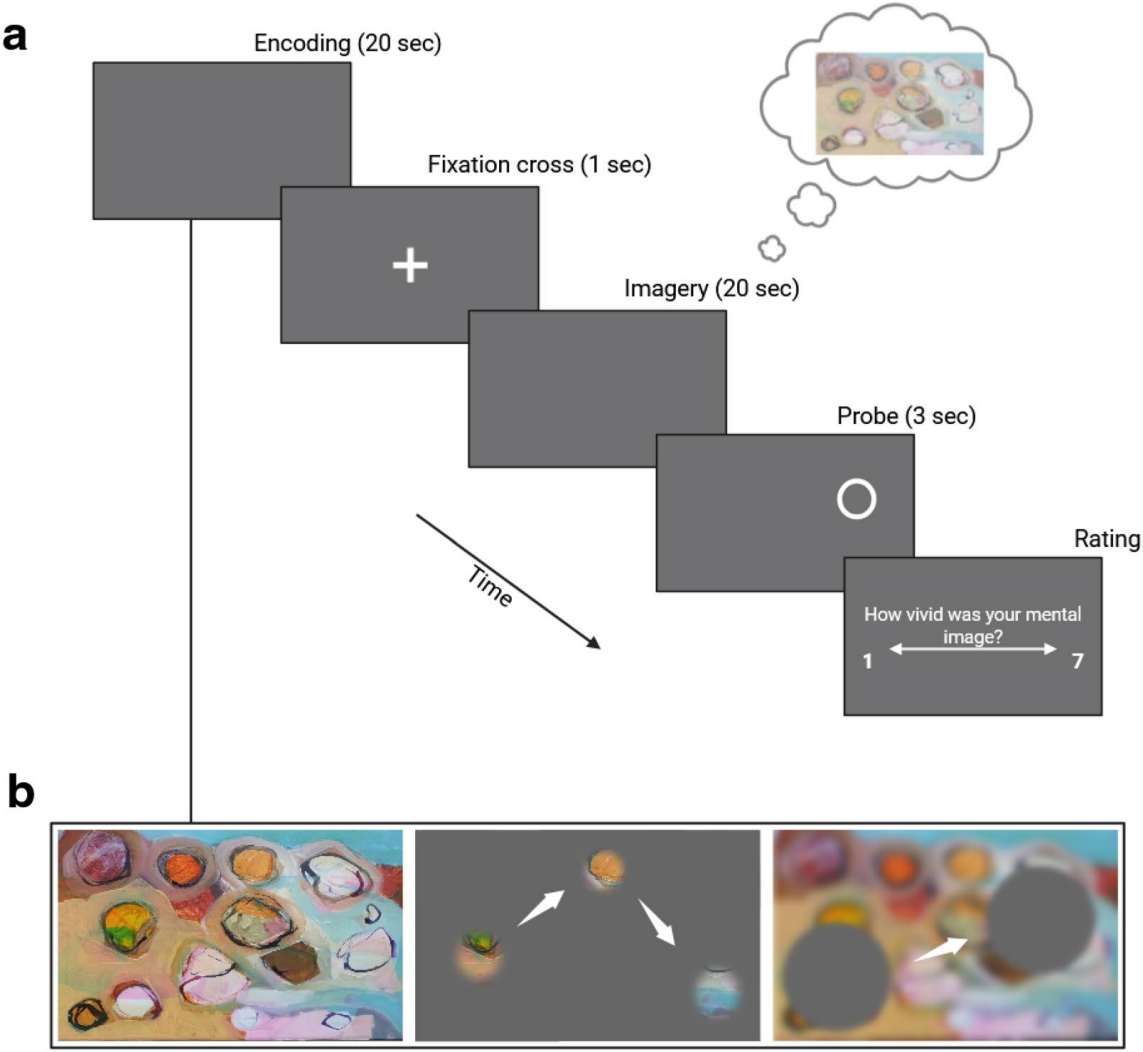
Procedure for Experiment 2. (**a**) First, participants encoded pictures from three categories (art, indoor, and outdoor scenes) either freely, with a gaze-contingent window (GCW), or with an artificial scotoma (AS) for 20 sec, before a 1 sec fixation cross. Afterwards, they were instructed to imagine the picture as vividly as possible for 20 sec, before a probe appeared during the 3 last seconds, and the participants were required to judge whether a specific object or color fell under the probe. At the conclusion of each trial, participants had to rate the vividness of their mental image from 1 to 7. (**b**) Illustration of the 3 encoding conditions: free perception (left), encoding with the GCW (center), and with the AS (right). In the AS illustration, the image is blurred to illustrate that participants saw the picture with peripheral vision.

In Experiment 2, we manipulate encoding before participants imagine the picture (see Figure 4). If gaze patterns during imagery mirror part-based viewing regardless of how the pictures are encoded, this would suggest that part-by-part construction is an intrinsic property of imagery. However, if gaze patterns during imagery vary depending on the encoding type, part-based eye movements in Experiment 1 could have been influenced by free encoding. Experiment 2 also addresses a potential confound in Experiment 1, where participants always viewed and imagined the scenes before reinspection with the GCW or AS, potentially biasing gaze patterns during reinspection. Hence, Experiment 2 investigates whether the part-based pattern stems from perceptual encoding or whether it reflects a characteristic intrinsic to mental image generation.

#### Looking at nothing

We investigated whether the LAN effect differed after different encoding types. Similar to Experiment 1, the results reveal a significant main effect (estimate = 0.024, lower CI = 0.021, upper CI = 0.027), but no interaction between fixations during the initial encoding phase and the GCW condition (estimate = -0.002, lower CI = -0.006, upper CI = 0.003), nor between fixations during encoding and the AS condition (estimate = -0.001, lower CI = -0.005, upper CI = 0.003).

#### Scanpaths

The results show the same pattern of results as in Experiment 1: the vector similarity was higher between MI and GCW (estimate = 0.465, lower CI = 0.418, upper CI = 0.512) and lower between MI and AS (estimate = -0.193, lower CI = -0.233, upper CI = -0.152), compared to FP. The direction (estimate = 0.144, lower CI = 0.080, upper CI = 0.207) and length (estimate = 0.569, lower CI = 0.504, upper CI = 0.636) similarity were higher between MI-GCW compared to MI-FP. The position similarity was again lower between MI and GCW (estimate = -0.288, lower CI = -0.335, upper CI = -0.242), as well as between MI and AS (estimate = -0.105, lower CI = -0.152, upper CI = -0.057), compared to MI-FP. There was again no difference for the duration similarity, where the credible intervals for both MI-GCW (estimate = 0.073, lower CI = -0.005, upper CI = 0.150) and MI-AS (estimate = 0.050, lower CI = -0.027, upper CI = 0.129) included 0.

#### Temporal gaze dynamics

**Fig. 5:**
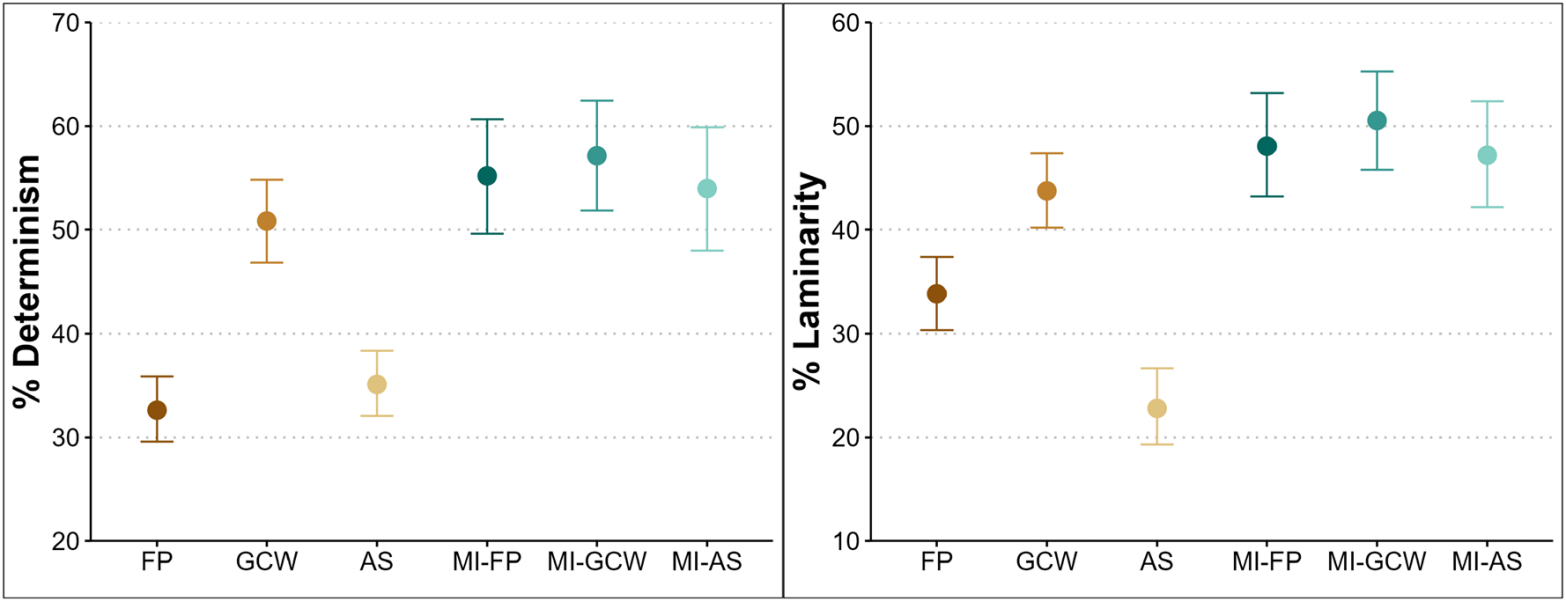
RQA results for Experiment 2. Determinism was higher during GCW encoding and the three imagery conditions (left). Laminarity was lower during AS encoding compared to the other encoding and imagery conditions (right). The plots demonstrate that determinism is similar between all three imagery conditions and GCW encoding. Laminarity differed substantially for AS encoding compared to the other encoding and imagery conditions. The shapes represent predicted values (estimates of the Bayesian model) while controlling for the fixation spread, and the bars represent the 95% credible intervals.

Results for determinism indicate higher values for MI after FP (estimate = 0.970, lower CI = 0.765, upper CI = 1.174), MI after GCW encoding (estimate = 1.023, lower CI = 0.778, upper CI = 1.269) and MI after AS encoding (estimate = 0.924, lower CI = 0.661, upper CI = 1.188), as well as for GCW encoding itself (estimate = 0.756, lower CI = 0.563, upper CI = 0.954) compared to FP (Fig. 5, left panel). Results for laminarity indicate higher values during MI following FP encoding (estimate = 0.623, lower CI = 0.438, upper CI = 0.811), GCW encoding (estimate = 0.698, lower CI = 0.463, upper CI = 0.931), and AS encoding (estimate = 0.599, lower CI = 0.358, upper CI = 0.841), compared to FP (Fig. 5, right panel). Similarly, GCW encoding showed increased laminarity (estimate = 0.418, lower CI = 0.220, upper CI = 0.615), while AS encoding showed reduced laminarity (estimate = -0.532, lower CI = -0.752, upper CI = -0.308).

**Fig. 6:**
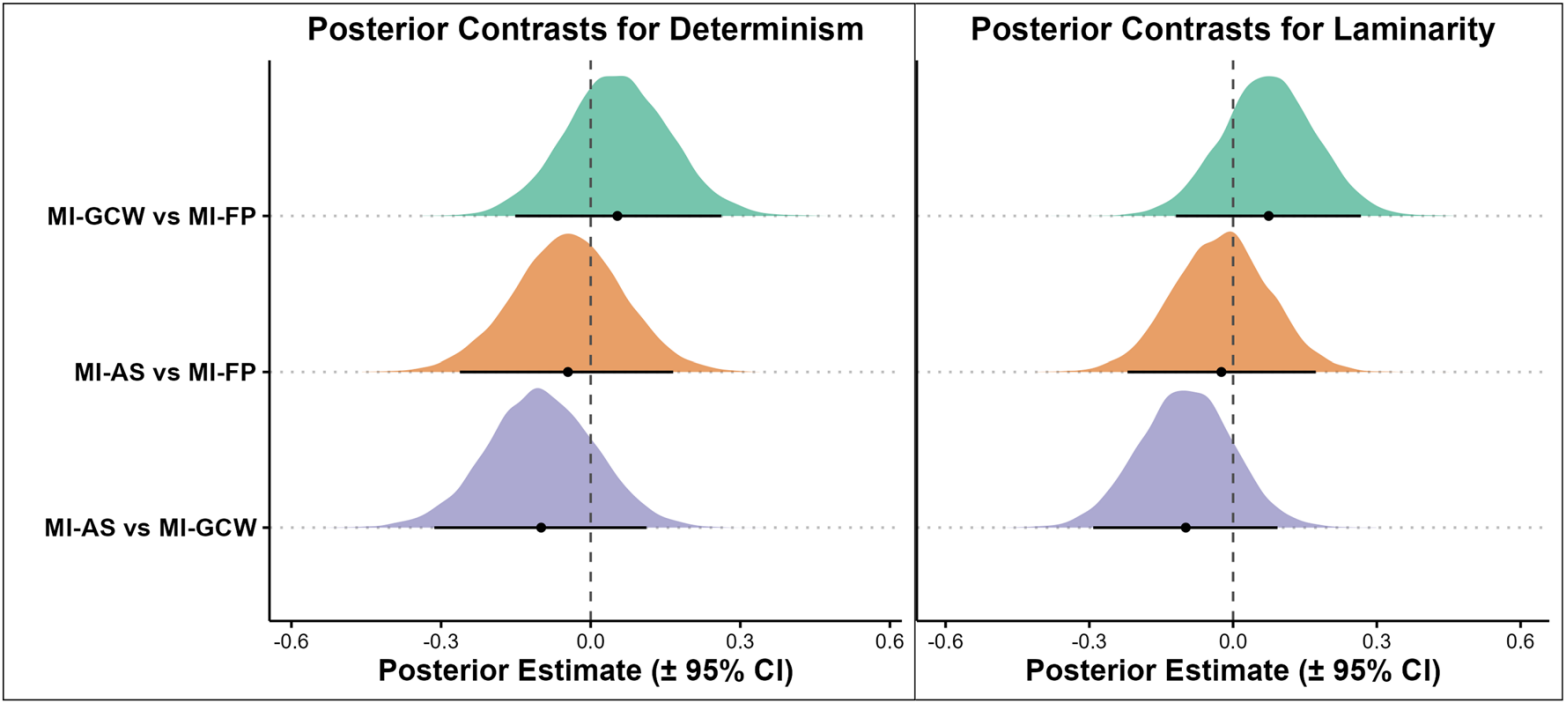
Posterior contrasts between mental imagery conditions for determinism (left) and laminarity (right) in Experiment 2. The plots display Bayesian model estimates of fixed-effect contrasts comparing mental imagery following different encoding types: free perception (MI-FP), gaze-contingent window (MI-GCW), and artificial scotoma (MI-AS). None of the 95% credible intervals excluded zero, indicating that determinism and laminarity during mental imagery did not substantially differ as a function of the prior encoding condition. Density shapes represent the posterior distributions of each contrast, and horizontal bars indicate the 95% credible intervals.

To investigate whether encoding influences subsequent MI, we compared determinism and laminarity between the three MI conditions (MI-FP, MI-GCW, MI-AS). Results for determinism indicate no differences between mental imagery conditions (Fig. 6, left panel). Credible intervals for the comparison between MI-GCW and MI-FP (estimate = 0.054, lower CI = -0.151, upper CI = 0.262), MI-AS and MI-FP (estimate = -0.046, lower CI = -0.262, upper CI = 0.165), and the comparison between MI-AS and MI-GCW (estimate = -0.099, lower CI = -0.314, upper CI = 0.112) all included zero, indicating that the type of encoding did not result in distinct gaze patterns during mental imagery. Similarly, results for laminarity indicate no differences between MI conditions (Fig. 6, right panel). Credible intervals for the comparison between MI-GCW and MI-FP (estimate = 0.074, lower CI = -0.119, upper CI = 0.267), MI-AS and MI-FP (estimate = -0.024, lower CI = -0.220, upper CI = 0.172), and the comparison between MI-AS and MI-GCW (estimate = -0.099, lower CI = -0.292, upper CI = 0.093) all included zero, indicating that the type of encoding did not result in distinct laminarity during imagery.

#### Probe and vividness

Participants answered the questions following the probes with an overall accuracy of 62%. Accuracy was 67% for FP trials, 66% for GCW trials, and 54% for AS trials. Accuracy by image type was 67% for abstract art, 64% for indoor scenes, and 55% for outdoor scenes.

The overall average vividness rating was 3.99 (on a 7-point scale). The average vividness rating was 4.16 for FP trials, 3.66 for GCW trials, and 4.16 for AS trials. By image type, vividness ratings were 2.78 for abstract art, 4.63 for indoor scenes, and 4.57 for outdoor scenes.

## General discussion

In this study, we investigated the role of eye movements during visual mental imagery by comparing gaze patterns during imagery to those observed in part-based, holistic, and free perception. The results of Experiment 1 show that gaze patterns during imagery closely resemble those observed when pictures are encoded part-by-part (gaze-contingent window). Experiment 2 shows that this similarity persists regardless of whether the pictures were originally encoded freely, part-by-part, or holistically. This suggests that part-based patterns during imagery are independent of prior encoding. The results provide evidence that mental images are generated by assembling distinct elements^31^, and that eye movements reflect this construction process.

Several studies on gaze patterns during mental imagery suggest a reactivation of spatial indices tied to different parts of a picture to enable perceptual recall^5,6,13^. It has been concluded that eye movements organize the reassembly of mental images from individual parts^6,7^, but this hypothesis has not been tested directly. Moreover, these studies did not investigate the specific reasons why gaze patterns during imagery often differ from those during perception. It also remained unclear to what extent imagery-related eye movements depend on encoding. In the present study, we tested whether imagery-related gaze patterns reflect a part-by-part construction process, and whether this process depends on the initial encoding.

The results showed highest similarity in both experiments when comparing MI to part-based encoding (GCW). This finding challenges the assumption that free perception and imagery involve similar scanpaths, despite the LAN effect being consistently reported in the literature. Previous studies have mostly shown the LAN effect using area of interest (AOI) analyses^1,4,6,7,11^, and we replicate this in both experiments: participants re-fixated the same locations during imagery as during perception. However, the LAN effect did not differ across conditions, suggesting that AOI analyses are not sensitive enough to detect subtle differences in fixation scanpaths, such as those distinguishing between holistic and part-based viewing. Previous studies have shown that although the spatial distribution of fixations is similar in free viewing and mental imagery, this similarity becomes less systematic when investigating scanpaths and the temporal gaze patterns^27–29^. Indeed, our RQA and MultiMatch results show that the temporal gaze dynamics and fixation scanpaths during imagery resemble those observed during part-based viewing. Although eye fixations during imagery land in similar screen locations as during free perception, the underlying scanpaths and refixation patterns differ. Thus, gaze patterns during imagery are not merely a replay of fixations from perception.

Why do gaze patterns during imagery resemble part-based encoding? Visual imagery, like perception, involves two components: object imagery, which emphasizes visual details such as shape and color, and spatial imagery, which represents the spatial arrangement and configuration of objects^48,49^. Importantly, eye movements during imagery are tied to the spatial aspects of mental images. For instance, restricting eye movements during imagery impairs memory for spatial relations between objects rather than object features^8^. Indeed, keeping central fixation during imagery leads to a more holistic imagery strategy resulting in less detailed recall^5,6^. Individuals with less spatial imagery abilities show increased eye movements during imagery^50^, suggesting that eye fixations help to construct the spatial layout of mental images. These findings indicate that eye movements supporting part-based processing are essential for constructing spatially detailed mental images. Thus, because eye movements are tied to the spatial aspects of imagery^8,50^, and spatial imagery relies on part-by-part construction^48^, this may explain why gaze patterns during mental imagery resemble those observed during part-based encoding.

The results of Experiment 2 provide further insight into the origin of part-based gaze patterns during imagery. Despite clear differences in perceptual encoding between the AS and GCW conditions, participants showed similar gaze patterns during subsequent imagery. These findings are consistent with studies showing that imagery-related eye movements do not necessarily reflect those made during encoding^5,11,50^. In a previous study^5^, participants maintained central fixation during encoding, yet they still showed spread out fixations during imagery. However, it is possible that covert attention shifts during encoding accounted for later eye movements. Covert attention shifts are known to support memory retrieval^47^ similar to overt eye movements. This could explain why there were eye movements during imagery without overt gaze shifts during perceptual encoding. Here, in the AS condition, covert attention shifts were encouraged because fixating an area made it disappear immediately. In contrast, the GCW condition required overt attention (e.g.^51^), because participants could only see the part of the image they directly fixated. Since gaze patterns during imagery did not change across encoding conditions, covert attention shifts during perception cannot account for eye fixations during imagery. Instead, eye movements during imagery reflect an internal process that operates independently of prior encoding. This suggests that imagery-related gaze patterns are not reinstated from perception, but instead reflect the part-by-part generation and maintenance of mental images.

Mental images are fragile and begin to fade once a part is activated, and they need to be maintained by repeatedly re-focusing attention on the same parts^52^. Since eye movements overtly reflect the spatial focus of attention^53^, they may serve as external markers of how each part of the mental image is assembled and maintained. Increased determinism and laminarity support this interpretation: clustered refixations following the same sequential order likely reflect the systematic reactivation of image parts. Crucially, eye movements were independent of how the image was originally encoded (freely, part-by-part, or holistically). While the initial content retrieved from memory may vary depending on the conditions of encoding, the way the image is mentally constructed relies on a part-based process. Hence, eye movements during imagery reflect shifts in spatial attention to arrange and maintain parts of a mental image, and this process is independent of how the information was encoded.

When recalling spoken facts, participants tend to fixate the screen location where a speaker had previously appeared, indicating that spatial indices can guide eye movements even when the remembered information is purely auditory and semantic^11^. In fact, Ferreira et al. (2008)^3^ proposed that eye movements are guided by internally constructed spatial indices, regardless of whether they were originally derived from visual, linguistic, or conceptual input. Likewise, the current results show that imagery-related eye movements reflect internally constructed mental representations, independent of how the information was visually encoded. Thus, the current findings suggest that mental images are not stored as holistic representations in memory, but are assembled part-by-part during recall. Gaze patterns during imagery consistently resemble those observed during part-based viewing, even when the image was originally perceived holistically.

Several limitations must be acknowledged. While there are no established standards for selecting the threshold radius in RQA, we followed the approach used in previous studies^28,29,54,55^ to ensure comparability of results. Another potential limitation concerns the probe task, which appeared after the imagery phase and asked participants to recall specific visual details (e.g., an object or a color). This may have encouraged attention toward specific image regions and, in principle, biased gaze patterns during imagery toward part-based viewing. However, in Experiment 1, the probe was only shown after the second imagery phase, whereas our analyses were based exclusively on the first imagery phase, which did not include a probe. Moreover, despite imagery always being followed by a probe in Experiment 2, the pattern of results closely matched those of Experiment 1. This makes it unlikely that the observed effects were driven by a task-induced focus on specific image regions. Finally, the similarity between imagery and part-based viewing may arise from the fact that we used complex scenes as visual stimuli. Previous studies showed that eye movements follow more systematic and repeated scanpaths under high memory workload^46^. Future studies could investigate whether these part-based patterns persist with less complex visual stimuli.

To conclude, the present study provides direct evidence that eye movements during mental imagery are not simply replayed from perception. Instead, they resemble the spatiotemporal dynamics of part-based viewing, suggesting that they support the construction of mental images piece by piece. Crucially, this pattern emerged regardless of whether the scenes were initially encoded freely, holistically, or part-by-part. Mental images are not retrieved as holistic visual representations from memory. Instead, they are reconstructed part-by-part during recall, and eye movements reflect this generative process.

## Methods

### Participants

For Experiment 1, 52 participants (mean = 22.77, sd = 2.53, range = 19-35, female = 42) were recruited for the study. Three participants were excluded. One because a lot of tracking time was missing, and two because of unusual gaze behavior (see supplementary materials S7.1 for details). The final sample consisting of 49 participants (mean = 22.80, sd = 2.59, range = 19-35, female = 39) was kept for subsequent analyses. For Experiment 2, 55 new participants that did not participate in the first experiment were recruited. Two participants were excluded because of calibration issues during the eye-tracking experiment, and 3 participants had to be excluded because their gaze behavior was highly unusual (see supplementary materials S7.2 for details). This resulted in a final sample of 50 participants (mean = 22.72, sd = 3.16, range = 18 - 34, female = 39). Exclusion criteria for both experiments were the use of medication that can impair consciousness or vision or wearing glasses. Furthermore, participants were asked if they were able to generate mental images, and those with self-reported aphantasia were excluded. All participants gave their informed consent prior to the study. The study was approved by the Ethics Committee of the Faculty of Human Sciences at the University of Bern. All methods were performed in accordance with the ethical standards of the institutional research committee and with the 1964 Declaration of Helsinki and its later amendments or comparable ethical standards.

### Apparatus

Fixation data was acquired with an EyeLink 1000 Plus eye-tracker (SR Research, Canada). For each participants the dominant eye was determined using the Miles Test^56^ and tracked with a sampling rate of 1000 Hz. Fixations were defined as the absence of saccade and blink^57^. Thus, each sample was considered as part of a fixation when both the velocity and acceleration were below a threshold of $30^{\circ }\hbox {/sec}$ and $8000^{\circ }\hbox {/sec}$, respectively. When the pupil was missing, very small, or distorted, samples were not labeled as part of a fixation. The fixation data was exported with the SR research Data Viewer software (SR Research Ltd., version 4.3.210) for preprocessing and further analyses.

### Gaze-contingent windows

Two types of gaze-following objects were used for the experiment. The first was a gaze-contingent window (GCW) which is comparable to “tunnel vision” where only the center of the current fixation position is visible, the peripheral part being covered with gray pixels. The second was an artificial scotoma (AS), where the center of the current fixation location was covered in gray pixels, and thus only peripheral information remained visible. We conducted a small pretest (n=5) to determine which eye-tracking parameters led to the smoothest gaze-following with the least possible latency. We concluded that there were no subjective differences between using both eyes or only the dominant eye to move the window. Furthermore, monocular tracking with the EyeLink 1000 Plus allows for a sampling rate of 1000 Hz, whereas binocular was limited to 500 Hz. Thus, we decided to track the dominant eye and use its position to move the GCW and the AS. The size of the GCW corresponded to $5^{\circ }$ of the visual angle, which in our case was 270 pixels diameter. The size of the AS was 377 pixels diameter, corresponding to $7^{\circ }$ of visual angle. The AS was bigger than the GCW, as prior research suggests that smaller central scotomas do not sufficiently interfere with scene processing or fixation behavior^58^. Both masks were created in Photoshop^59^, and a Gaussian filter was applied to make the edges less sharp, and reduce afterimages.

### Stimuli and materials

45 pictures (15 abstract art, 15 indoor, and 15 outdoor pictures) were displayed at a distance of 855 mm from the participants on a 1920 $\times$ 1080 computer screen with a refresh rate of 144Hz. Pictures of indoor and outdoor scenes were selected from the FIGRIM^60^ and LaMem^61^ databases. Details about the selection of stimuli can be found in the supplementary material S1.

For Experiment 2, the same set of 45 stimuli (15 from each category) was used as in Experiment 1. However, each picture was shown only once per condition (GCW, AS, FP), resulting in 15 pictures per condition, with 5 from each category.

The VVIQ 2 was used to assess vividness in mental imagery^62^, and participant’s cognitive styles with the OSIVQ^63^. We used Vanderberg’s task^64^ to assess mental rotation, and a visual n-back task for visual working memory programmed in MATLAB^65^ with PsychToolbox-3^66^. These were used for exploratory purposes and more information can be found in the supplementary material S6.

### Procedure

#### Experiment 1

First, participants read a cover story, telling them that we were measuring pupil dilatation with respect to image complexity. This was to avoid that participants focus on their eye movements during mental imagery. Subsequently, participants read the instructions and a 9-point-calibration was performed after determining the infrared light intensity threshold. The experiment began with a habituation phase with 10 GCW trials and 10 AS trials. Each trial lasted 10 sec, and consisted of images different from those used in the experimental phase. Additionally, there were 2 practice trials (one with GCW and one with AS). Each trial started with a drift correction dot. First, a picture was displayed during 20 sec, followed by a fixation cross for 1 sec. Then, a blank gray screen appeared during 20 sec, on which participants had to visually imagine the picture they just saw as vividly and precisely as possible. Longer duration during imagery ensures that eye movements do not merely stem from visual after effects, and are required to gather enough data for RQA. Subsequently, participants were asked how precisely and vividly their mental image was, and had to press a key from 1 (no imagery) to 7 (like a real picture). After, participants saw a fixation cross (1 sec) and the same image again for 20 sec with either a GCW (block 1) or an AS (block 2). Finally, participants had to imagine the picture again on a blank gray screen displayed for 10 sec, and a probe appeared during the last 3 seconds, followed by a question whether it appeared on a specific object of the previously perceived image. Participants had to answer “yes” or “no” by pressing a key on the keyboard and the amount of correct responses being “yes” and “no” were equal. A second imagery phase was necessary for the probe, to avoid that participants focus their fixations on verifying their response accuracy regarding the probe during the reinspection. The experiment consisted of 2 blocks, one in which the image was reinspected with a GCW, and with an AS in the other block. Both the order of blocks and images was randomized, and the same images were used in both the GCW and AS blocks. This allowed us to compare eye movements during imagery with those during GCW and AS for the same pictures. Each block lasted approximately 1 hour, with a 10 minutes break in-between. After the eye-tracking experiment, participants completed the OSIVQ, the mental rotation task, the VVIQ, and the visual n-back task. More information about the n-back task can be found in the supplementary material S6.1.4. To conclude the experiment, participants were asked what they believed the aim of the eye-tracking experiment was, before a debriefing. None of the participants guessed the real purpose of the experiment.

#### Experiment 2

The procedure was essentially the same as in Experiment 1, except that there was no reinspection phase, and the encoding type was manipulated during the first encoding. Like in Experiment 1, participants read a cover story, telling them that we were measuring pupil dilatation with respect to image complexity. The experiment also began with a habituation phase with 10 GCW trials and 10 AS trials, and there were 3 practice trials (one for each condition, FP, GCW and AS). First, a picture was displayed during 20 sec and encoded either freely (block 1), with a GCW (block 2), or with an AS (block 3), followed by a fixation cross for 1 sec. Then, a blank gray screen appeared during 20 sec, on which participants had to visually imagine the picture they just saw, and a probe appeared for 3 seconds, followed by a question whether it appeared on a specific object of the previously perceived image. At the end of each trial, participants had to press a key from 1 (no imagery) to 7 (like a real picture). The block order as well as the stimulus presentation within each block were randomized.

After the eye-tracking experiment, participants completed exactly the same tasks as described in Experiment 1 (i.e. VVIQ, OSIVQ, mental rotation, and n-back). To conclude the experiment, participants were asked what they believed the aim of the eye-tracking experiment was, before a debriefing. None of the participants guessed the real purpose of the experiment.

### Eye movement analyses

Raw fixation reports from the Data Viewer software were preprocessed to remove fixations outside of the screen, and fixations longer than 5000 ms or shorter than 100 ms. The time spent on fixating the screen as well as the number and duration of fixations for each participant were compared in relation to the other participants to detect outliers in gaze behavior and potential technical problems. A detailed report can be found in the supplementary materials S7.

### Area of interest analysis

To investigate the LAN effect, we separated the screen into four equally sized quadrants (AOIs), following the procedure of previous studies^7,28^. For each trial and participant, we calculated the amount of fixations falling in each AOI within the different experimental phases. Then, we tested whether fixations during mental imagery are predicted by fixations during encoding. Thus, the fixation locations during visual imagery were used as an outcome variable, and fixation locations during the other phases as predictors.

### MultiMatch

MultiMatch^67,68^ computes the similarity between two scanpaths represented as geometrical vectors across different dimensions. *Vector (shape)* refers to the similarity of the geometrical shape between two aligned vectors (saccade pairs) regardless of their relative position in space. *Length* compares the similarity between the amplitude of two saccades, in terms of length between endpoints. *Direction* indicates whether the angular distance between two vectors is similar. *Position* compares the Euclidean distance between the fixation points of two aligned vectors. Finally, *Duration* represents the similarity of the temporal duration between aligned fixations. In the current study, these similarity metrics were computed for the comparisons between mental imagery and the different encoding conditions (FP, GCW, and AS) for each participant and trial.

### Recurrence quantification analysis

While MultiMatch quantifies and compares gaze scanpaths in the spatial domain, recurrence quantification analysis (RQA) is a method providing various measures to characterize the temporal dynamics of fixation data. RQA provides a non-linear analysis of complex dynamic systems. More recently, RQA has been successfully implemented for the analysis of temporal dynamics of gaze patterns^43,69^. Importantly, RQA parameters have been used to reveal unique temporal properties of eye movements during mental imagery, and allowed to distinguish gaze properties that are unique to imagery^27,28^.

#### Recurrence

Recurrence is the fundamental unit in RQA which is then used to compute several other parameters. Two fixations are considered recurrent if the Euclidean distance between them is equal or below a given distance threshold. Recurrent fixations are usually represented in a two dimensional recurrence plot, where each axis represents the sequence of fixations, denoted as $S$, from the first ($S_1$) to the last ($S_n$). The Euclidean distance is calculated for each pair ($S_i$, $S_j$) of fixations within this sequence. If this distance is less than or equal to a predetermined threshold, indicating that the fixations are spatially close, a dot is plotted at the coordinates $(i, j)$ on the recurrence plot. Each dot on a recurrence plot shows that the fixations at positions $i$ and $j$ within the sequence $S$ are considered recurrent. Thus, a square recurrence matrix can be derived with:

1 $$\begin{aligned} R_{i,j} = \Theta (\epsilon - \Vert x_i - x_j \Vert ) \end{aligned}$$

where $R_{i,j}$ represents an element of the recurrence matrix, indicating whether the fixations $x_i$ and $x_j$ are recurrent within the threshold $\epsilon$. $\Theta$ denotes the Heaviside step function, which is 1 if its argument is non-negative (i.e., $x_i$ and $x_j$ are within the threshold distance $\epsilon$) and 0 otherwise. $\Vert x_i - x_j\Vert$ is the chosen distance (in our case Euclidean) between the fixations $x_i$ and $x_j$^70^. The recurrence threshold was set to $2.5^{\circ }$ of visual angle (135px in our setup). Similar threshold values have been used in eye-tracking RQA studies^55,69,71^, including prior work on eye movements during mental imagery^27,28^, allowing direct comparison of parameter values. Thresholds in this range correspond to a spatial scale that encompasses the fovea and extends into the parafoveal region, which provides a sensible spatial radius for identifying recurrent fixations^69^.

#### Determinism

Different RQA measures are extracted from scale structures of the recurrence matrix defined above, such as vertical and diagonal lines. Determinism shows refixations that occur in the same sequential order as previous fixations. It is computed as the percentage of recurrence points which lie on diagonal lines in the recurrence matrix:

2 $$\begin{aligned} \text {DET} = 100 \cdot \frac{\sum _{L=L_{\text {min}}}^{n} D_L}{\sum _{i,j=1}^{n} (1 - \delta _{ij}) R_{i,j}} \end{aligned}$$

where $D_L$ represents the number of recurrence points in the recurrence matrix $R_{i,j}$ forming diagonal lines of a minimum length $L$. The Kronecker delta $\delta _{ij}$ is used in the equation to exclude the diagonal of self recurrence, where $i$ = $j$. A recurrence matrix is by definition symmetrical, thus only one triangle can be taken into account when computing determinism. The DET value increases when areas of an image are reinspected in the same sequential order for at least $L$ (generally $L = 2$) fixations. Therefore, a high DET value indicates that the order in which parts of an image are inspected is dependent on previous fixations on those same areas.

#### Laminarity

Laminarity represents the percentage of vertical and/or horizontal lines in the recurrence matrix, and can be defined as

3 $$\begin{aligned} \text {LAM} = 100 \cdot \frac{\sum _{L=L_{\text {min}}}^{n} H_L + V_L}{\sum _{i,j=1}^{n} (1 - \delta _{ij}) R_{i,j}} \end{aligned}$$

where $H_L$ and $V_L$ represent the number of recurrence points belonging to horizontal or vertical lines of minimum length $L$, respectively. A high LAM value either indicates that areas were first inspected with a single fixation, and later reinspected with at least $L$ refixations (vertical lines); or that areas were initially inspected in detail before being briefly reinspected with a single fixation (horizontal lines).

### Data analysis

#### Implementation details

To compute the different RQA parameters, we used the MATLAB functions from^43^. The MultiMatch parameters were computed in Python with the re-implementation of MATLAB functions^68^ into Python^72^.

#### Statistical analyses

All statistical analyses were performed with R^73^, in RStudio^74^, with Bayesian hierarchical generalized regression models implemented in the brms package^75^. In all our models to estimate RQA parameters, we used zero-one-inflated beta (zoib) regressions, as they are suitable for proportion data while accounting for zero and one’s present in the data (unlike simple beta regressions). Moreover, zoib regressions have the potential to model the zero-one inflation (i.e. the probability of a score being exactly zero or one, denoted as zoi) as well as the conditional one inflation (i.e. if a score is exactly zero or one, its probability of being one, denoted as coi). For the MultiMatch analyses, we used Beta regressions because all similarity scores were bounded between 0 and 1. Thus, the model does not predict values outside the range of possible values. Bayesian models provide posterior distributions for each estimated parameter. This distribution represents the probability of different parameter values given the observed data and prior information. From these, we derive credible intervals, which indicate the range within which the true parameter likely falls. A 95% credible interval means there is a 95% probability that the true parameter value lies within this interval. If this interval does not include zero, it suggests the presence of an effect. The brms package uses Hamiltonian Monte Carlo (HMC), as MCMC sampling method. We used 4 Markov-Chains with 6000 iterations each to ensure convergence in all our models. Both the pareto-k and visual inspection of the posterior predictive distribution were used as a criteria to assess the goodness of fit of all our models. Posterior predictive checks consist in comparing the distribution of the observed data to the distribution of data generated based on the parameters specified in a given model. If these two distributions look similar, it suggests good fit. To assess model convergence and sampling efficiency, we examined the potential scale reduction factor (Rhat), bulk effective sample size, and tail effective sample size. All model information and specifications can be found in the supplementary material S2 (Experiment 1) and S3 (Experiment 2).

## Supplementary Information

Supplementary Information.

## Data Availability

All materials, data, and analysis scripts are made publicly available: https://osf.io/zaht8/?view_only=bea57fcc57c14c05aeb6815e4d080839.

## References

[1] Altmann, G. T. M. Language-mediated eye movements in the absence of a visual world: The ‘blank screen paradigm’. *Cognition* **93**, B79–B87. 10.1016/j.cognition.2004.02.005 (2004). 15147941 10.1016/j.cognition.2004.02.005

[2] Johansson, R., Holsanova, J. & Holmqvist, K. Pictures and spoken descriptions elicit similar eye movements during mental imagery, both in light and in complete darkness. *Cognit. Sci.* **30**, 1053–1079. 10.1207/s15516709cog0000_86 (2006). 21702846 10.1207/s15516709cog0000_86

[3] Ferreira, F., Apel, J. & Henderson, J. M. Taking a new look at looking at nothing. *Trends Cognit. Sci.* **12**, 405–410. 10.1016/j.tics.2008.07.007 (2008). 18805041 10.1016/j.tics.2008.07.007

[4] Martarelli, C. S. & Mast, F. W. Eye movements during long-term pictorial recall. *Psychol. Res.* **77**, 303–309. 10.1007/s00426-012-0439-7 (2013). 22610303 10.1007/s00426-012-0439-7

[5] Johansson, R., Holsanova, J., Dewhurst, R. & Holmqvist, K. Eye movements during scene recollection have a functional role, but they are not reinstatements of those produced during encoding. *J. Exp. Psychol. Hum. Percept. Perform.* **38**, 1289–1314. 10.1037/a0026585 (2012). 22201467 10.1037/a0026585

[6] Laeng, B. & Teodorescu, D.-S. Eye scanpaths during visual imagery reenact those of perception of the same visual scene. *Cognit. Sci.* **26**, 207–231. 10.1207/s15516709cog2602_3 (2002).

[7] Laeng, B., Bloem, I. M., D’Ascenzo, S. & Tommasi, L. Scrutinizing visual images: The role of gaze in mental imagery and memory. *Cognition* **131**, 263–283. 10.1016/j.cognition.2014.01.003 (2014). 24561190 10.1016/j.cognition.2014.01.003

[8] Johansson, R. & Johansson, M. Look here, eye movements play a functional role in memory retrieval. *Psychol. Sci.* **25**, 236–242. 10.1177/0956797613498260 (2014). 24166856 10.1177/0956797613498260

[9] Foulsham, T. & Kingstone, A. Fixation-dependent memory for natural scenes: An experimental test of scanpath theory. *J. Exp. Psychol. Gen.* **142**, 41–56. 10.1037/a0028227 (2013). 22506754 10.1037/a0028227

[10] Mast, F. W. & Kosslyn, S. M. Eye movements during visual mental imagery. *Trends Cognit. Sci.* **6**, 271–272. 10.1016/S1364-6613(02)01931-9 (2002). 12110350 10.1016/s1364-6613(02)01931-9

[11] Richardson, D. C. & Spivey, M. J. Representation, space and hollywood squares: Looking at things that aren’t there anymore. *Cognition* **76**, 269–295. 10.1016/S0010-0277(00)00084-6 (2000). 10913578 10.1016/s0010-0277(00)00084-6

[12] Hoover, M. A. & Richardson, D. C. When facts go down the rabbit hole: Contrasting features and objecthood as indexes to memory. *Cognition* **108**, 533–542. 10.1016/j.cognition.2008.02.011 (2008). 18423431 10.1016/j.cognition.2008.02.011

[13] Kumcu, A. & Thompson, R. L. Spatial interference and individual differences in looking at nothing for verbal memory. In *Proceedings of the Annual Meeting of the Cognitive Science Society*. Vol. 38 (2016).

[14] Hesslow, G. The current status of the simulation theory of cognition. *Brain Res.* **1428**, 71–79. 10.1016/j.brainres.2011.06.026 (2012). 21763643 10.1016/j.brainres.2011.06.026

[15] Moulton, S. T. & Kosslyn, S. M. Imagining predictions: Mental imagery as mental emulation. *Philos. Trans. R. Soc. B Biol. Sci.* **364**, 1273–1280. 10.1098/rstb.2008.0314 (2009). 10.1098/rstb.2008.0314 PMC2666712 19528008

[16] Dijkstra, N., Zeidman, P., Ondobaka, S., van Gerven, M.A. J. & Friston, K. Distinct top-down and bottom-up brain connectivity during visual perception and imagery. *Sci. Rep.* **7**, 5677. 10.1038/s41598-017-05888-8 (2017). 10.1038/s41598-017-05888-8 PMC5516016 28720781

[17] Xie, S., Kaiser, D. & Cichy, R. M. Visual imagery and perception share neural representations in the alpha frequency band. *Curr. Biol.* **30**, 2621-2627.e5. 10.1016/j.cub.2020.04.074 (2020). 32531274 10.1016/j.cub.2020.04.074 PMC7342016

[18] Dijkstra, N., Bosch, S. E. & Gerven, M. A.J.V. Vividness of visual imagery depends on the neural overlap with perception in visual areas. *J. Neurosci.* **37**, 1367–1373. 10.1523/JNEUROSCI.3022-16.2016 (2017). 10.1523/JNEUROSCI.3022-16.2016 PMC6596858 28073940

[19] Pearson, J., Clifford, C. W. G. & Tong, F. The functional impact of mental imagery on conscious perception. *Curr. Biol.* **18**, 982–986. 10.1016/j.cub.2008.05.048 (2008). 18583132 10.1016/j.cub.2008.05.048 PMC2519957

[20] Dijkstra, N., Mazor, M., Kok, P. & Fleming, S. Mistaking imagination for reality: Congruent mental imagery leads to more liberal perceptual detection. *Cognition* **212**, 104719. 10.1016/j.cognition.2021.104719 (2021). 33878636 10.1016/j.cognition.2021.104719 PMC8164160

[21] Moro, V., Berlucchi, G., Lerch, J., Tomaiuolo, F. & Aglioti, S. M. Selective deficit of mental visual imagery with intact primary visual cortex and visual perception. *Cortex J. Devot. Study Nervous Syst. Behav.* **44**, 109–118. 10.1016/j.cortex.2006.06.004 (2008). 10.1016/j.cortex.2006.06.004 18387540

[22] Behrmann, M., Winocur, G. & Moscovitch, M. Dissociation between mental imagery and object recognition in a brain-damaged patient. *Nature* **359**, 636–637. 10.1038/359636a0 (1992). 1406994 10.1038/359636a0

[23] Behrmann, M., Moscovitch, M. & Winocur, G. Intact visual imagery and impaired visual perception in a patient with visual agnosia. *J. Exp. Psychol. Hum. Percept. Perform.* **20**, 1068–1087. 10.1037/0096-1523.20.5.1068 (1994). 7964528 10.1037//0096-1523.20.5.1068

[24] Hebb, D. O. Concerning imagery. *Psychol. Rev.* **75**, 466–477. 10.1037/h0026771 (1968). 4973669 10.1037/h0026771

[25] Neisser, U. Cognitive psychology. In *Century Psychology Series* (Appleton-Century-Crofts, 1967) (OCLC: 192730).

[26] Bourlon, C., Oliviero, B., Wattiez, N., Pouget, P. & Bartolomeo, P. Visual mental imagery: What the head’s eye tells the mind’s eye. *Brain Res.* **1367**, 287–297. 10.1016/j.brainres.2010.10.039 (2011). 20969839 10.1016/j.brainres.2010.10.039

[27] Gurtner, L. M., Bischof, W. F. & Mast, F. W. Recurrence quantification analysis of eye movements during mental imagery. *J. Vis.* **19**, 17. 10.1167/19.1.17 (2019). 30699229 10.1167/19.1.17

[28] Gurtner, L. M., Hartmann, M. & Mast, F. W. Eye movements during visual imagery and perception show spatial correspondence but have unique temporal signatures. *Cognition* **210**, 104597. 10.1016/j.cognition.2021.104597 (2021). 33508576 10.1016/j.cognition.2021.104597

[29] Gurtner, L. M., Bischof, W. F. & Mast, F. W. Gaze restriction and reactivation of place-bound content drive eye movements during mental imagery. *J. Cognit.* **6**. 10.5334/joc.316 (2023). 10.5334/joc.316 PMC10473167 37663138

[30] Peelen, M. V., Berlot, E. & de Lange, F. P. Predictive processing of scenes and objects. *Nat. Rev. Psychol.* **3**, 13–26. 10.1038/s44159-023-00254-0 (2024). 38989004 10.1038/s44159-023-00254-0 PMC7616164

[31] Kosslyn, S. M., Thompson, W. L. & Ganis, G. *The Case for Mental Imagery* (Oxford University Press, 2006).

[32] van Diepen, P. M. J., Wampers, M. & d’Ydewalle, G. Functional division of the visual field: Moving masks and moving windows. In *Eye Guidance in Reading and Scene Perception*. 337–355. 10.1016/B978-008043361-5/50016-X (Elsevier Science Ltd, 1998).

[33] Hagen, S. et al. A perceptual field test in object experts using gaze-contingent eye tracking. *Sci. Rep.* **13**, 11437. 10.1038/s41598-023-37695-9 (2023). 37454134 10.1038/s41598-023-37695-9 PMC10349839

[34] Van Belle, G. et al. Impairment of holistic face perception following right occipito-temporal damage in prosopagnosia: converging evidence from gaze-contingency. *Neuropsychologia* **49**, 3145–3150. 10.1016/j.neuropsychologia.2011.07.010 (2011). 21802435 10.1016/j.neuropsychologia.2011.07.010

[35] Van Belle, G., De Graef, P., Verfaillie, K., Rossion, B. & Lefèvre, P. Face inversion impairs holistic perception: Evidence from gaze-contingent stimulation. *J. Vis.* **10**. 10.1167/10.5.10 (2010). 10.1167/10.5.10 20616142

[36] Bombari, D., Mast, F. W. & Lobmaier, J. S. Featural, configural, and holistic face-processing strategies evoke different scan patterns. *Perception* **38**, 1508–1521. 10.1068/p6117 (2009). 19950482 10.1068/p6117

[37] Schwarzer, G., Huber, S. & Dümmler, T. Gaze behavior in analytical and holistic face processing. *Mem. Cognit.* **33**, 344–354. 10.3758/BF03195322 (2005). 16028588 10.3758/bf03195322

[38] Brandt, S. A. & Stark, L. W. Spontaneous eye movements during visual imagery reflect the content of the visual scene. *J. Cognit. Neurosci.* **9**, 27–38. 10.1162/jocn.1997.9.1.27 (1997). 23968178 10.1162/jocn.1997.9.1.27

[39] Hassabis, D. & Maguire, E. A. The construction system of the brain. *Philos. Trans. R. Soc. B Biol. Sci.* **364**, 1263–1271. 10.1098/rstb.2008.0296 (2009). 10.1098/rstb.2008.0296 PMC2666702 19528007

[40] Damiano, C. & Walther, D. B. Distinct roles of eye movements during memory encoding and retrieval. *Cognition* **184**, 119–129. 10.1016/j.cognition.2018.12.014 (2019). 30594878 10.1016/j.cognition.2018.12.014

[41] Wynn, J. S., Ryan, J. D. & Buchsbaum, B. R. Eye movements support behavioral pattern completion. *Proc. Natl. Acad. Sci.* **117**, 6246–6254. 10.1073/pnas.1917586117 (2020). 32123109 10.1073/pnas.1917586117 PMC7084073

[42] Martarelli, C. S. & Mast, F. W. Pictorial low-level features in mental images: Evidence from eye fixations. *Psychol. Res.* **86**, 350–363. 10.1007/s00426-021-01497-3 (2022). 33751199 10.1007/s00426-021-01497-3

[43] Anderson, N. C., Bischof, W. F., Laidlaw, K. E. W., Risko, E. F. & Kingstone, A. Recurrence quantification analysis of eye movements. *Behav. Res. Methods* **45**, 842–856. 10.3758/s13428-012-0299-5 (2013). 23344735 10.3758/s13428-012-0299-5

[44] Ballard, D. H., Hayhoe, M. M. & Pelz, J. B. Memory representations in natural tasks. *J. Cognit. Neurosci.* **7**, 66–80. 10.1162/jocn.1995.7.1.66 (1995). 23961754 10.1162/jocn.1995.7.1.66

[45] Ryan, J. D. & Villate, C. Building visual representations: The binding of relative spatial relations across time. *Vis. Cognit.* **17**, 254–272. 10.1080/13506280802336362 (2009).

[46] Meghanathan, R. N., Nikolaev, A. R. & van Leeuwen, C. Refixation patterns reveal memory-encoding strategies in free viewing. *Attent. Percept. Psychophys.* **81**, 2499–2516. 10.3758/s13414-019-01735-2 (2019). 10.3758/s13414-019-01735-2 PMC6848043 31044400

[47] Scholz, A., Klichowicz, A. & Krems, J. F. Covert shifts of attention can account for the functional role of “eye movements to nothing’’. *Mem. Cognit.* **46**, 230–243. 10.3758/s13421-017-0760-x (2018). 28975576 10.3758/s13421-017-0760-x

[48] Kozhevnikov, M., Kosslyn, S. & Shephard, J. Spatial versus object visualizers: A new characterization of visual cognitive style. *Mem. Cognit.* **33**, 710–726. 10.3758/BF03195337 (2005). 16248335 10.3758/bf03195337

[49] Blazhenkova, O. Vividness of object and spatial imagery. *Percept. Motor Skills* **122**, 490–508. 10.1177/0031512516639431 (2016). 27166329 10.1177/0031512516639431

[50] Johansson, R., Holsanova, J. & Homqvist, K. The dispersion of eye movements during visual imagery is related to individual differences in spatial imagery ability. *Proc. Annu. Meet. Cognit. Sci. Soc.* **33** (2011).

[51] Perkovic, S., Schoemann, M., Lagerkvist, C.-J. & Orquin, J. L. Covert attention leads to fast and accurate decision-making. *J. Exp. Psychol. Appl.* **29**, 78–94. 10.1037/xap0000425 (2023). 35511553 10.1037/xap0000425

[52] Kosslyn, S. M. *Image and Brain: The Resolution of the Imagery Debate*. Vol. viii. 516 (The MIT Press, 1994).

[53] Henderson, J. M. Visual attention and eye movement control during reading and picture viewing. In *Eye Movements and Visual Cognition: Scene Perception and Reading* (ed. Rayner, K.) 260–283 (Springer, 1992). 10.1007/978-1-4612-2852-3_15.

[54] Farnand, S., Vaidyanathan, P. & Pelz, J. B. Recurrence metrics for assessing eye movements in perceptual experiments. *J. Eye Mov. Res.* **9**. 10.16910/jemr.9.4.1 (2016).

[55] Vaidyanathan, P., Pelz, J., Alm, C., Shi, P. & Haake, A. Recurrence quantification analysis reveals eye-movement behavior differences between experts and novices. In *Proceedings of the Symposium on Eye Tracking Research and Applications*, ETRA ’14. 303–306. 10.1145/2578153.2578207 (Association for Computing Machinery, 2014).

[56] Miles, W. R. Ocular dominance in human adults. *J. Gen. Psychol.* **3**, 412–430. 10.1080/00221309.1930.9918218 (1930).

[57] Hessels, R. S., Niehorster, D. C., Nyström, M., Andersson, R. & Hooge, I. T. C. Is the eye-movement field confused about fixations and saccades? A survey among 124 researchers. *R. Soc. Open Sci.* **5**, 180502. 10.1098/rsos.180502 (2018). 30225041 10.1098/rsos.180502 PMC6124022

[58] Cornelissen, F. W., Bruin, K. J. & Kooijman, A. C. The influence of artificial scotomas on eye movements during visual search. *Optom. Vis. Sci.* **82**, 27. 10.1097/01.OPX.0000150250.14720.C5 (2005). 15630401

[59] Adobe. Adobe Photoshop (Computer Software, 2025).

[60] Bylinskii, Z., Isola, P., Bainbridge, C., Torralba, A. & Oliva, A. Intrinsic and extrinsic effects on image memorability. *Vis. Res.* **116**, 165–178. 10.1016/j.visres.2015.03.005 (2015). 25796976 10.1016/j.visres.2015.03.005

[61] Khosla, A., Raju, A. S., Torralba, A. & Oliva, A. Understanding and predicting image memorability at a large scale. In *2015 IEEE International Conference on Computer Vision (ICCV)*. 2390–2398. 10.1109/ICCV.2015.275 (2015).

[62] Marks, D. F. New directions for mental imagery research. *J. Ment. Imag.* **19**, 153–167 (1995).

[63] Blazhenkova, O. & Kozhevnikov, M. The new object-spatial-verbal cognitive style model: Theory and measurement. *Appl. Cognit. Psychol.* **23**, 638–663. 10.1002/acp.1473 (2009).

[64] Vandenberg, S. G. & Kuse, A. R. Mental rotations, a group test of three-dimensional spatial visualization. *Percept. Motor Skills* **47**, 599–604. 10.2466/pms.1978.47.2.599 (1978). 724398 10.2466/pms.1978.47.2.599

[65] Inc., T. M. *MATLAB Version: 9.13.0 (R2022b)*. (Natick, 2022).

[66] Brainard, D. H. The psychophysics toolbox. *Spatial Vis.* **10**, 433–436 (1997). 9176952

[67] Jarodzka, H., Holmqvist, K. & Nyström, M. A vector-based, multidimensional scanpath similarity measure. In *Proceedings of the 2010 Symposium on Eye-Tracking Research & Applications*, ETRA ’10. 211–218. 10.1145/1743666.1743718 (Association for Computing Machinery, 2010).

[68] Dewhurst, R. et al. It depends on how you look at it: Scanpath comparison in multiple dimensions with MultiMatch, a vector-based approach. *Behav. Res. Methods* **44**, 1079–1100. 10.3758/s13428-012-0212-2 (2012). 22648695 10.3758/s13428-012-0212-2

[69] Anderson, N. C., Anderson, F., Kingstone, A. & Bischof, W. F. A comparison of scanpath comparison methods. *Behav. Res. Methods* **47**, 1377–1392. 10.3758/s13428-014-0550-3 (2015). 25540126 10.3758/s13428-014-0550-3

[70] Webber, C., Ioana, C. & Marwan, N. *Recurrence Plots and Their Quantifications: Expanding Horizons: Proceedings of the 6th International Symposium on Recurrence Plots, Grenoble, France*, 17–19 June 2015 (2016).

[71] Farnand, S., Vaidyanathan, P. & Pelz, J. B. Recurrence metrics for assessing eye movements in perceptual experiments. *J. Eye Mov. Res.* **9**. 10.16910/jemr.9.4.1 (2016).

[72] Wagner, A. S., Halchenko, Y. O. & Hanke, M. multimatch-gaze: The MultiMatch algorithm for gaze path comparison in Python. *J. Open Source Softw.* **4**, 1525. 10.21105/joss.01525 (2019).

[73] R Core Team. *R: A Language and Environment for Statistical Computing*. (R Foundation for Statistical Computing, 2023).

[74] RStudio Team. *RStudio: Integrated Development Environment for R*. (RStudio, PBC., 2020).

[75] Bürkner, P.-C. brms: An R package for Bayesian multilevel models using Stan. *J. Stat. Softw.* **80**, 1–28. 10.18637/jss.v080.i01 (2017).

