# Supplementary material for: Gaze patterns during visual mental imagery reflect part-based generation
Source: Sci Rep. 2026 Jan 13;16:5108. doi: 10.1038/s41598-026-35447-z (PMC12876999; doi:10.1038/s41598-026-35447-z)
Supplement: Supplementary file 1 — Supplementary Information. [file 41598_2026_35447_MOESM1_ESM.pdf]

Gaze Patterns During Mental Imagery Reflect Part-Based Generation

Enea J. Weber[1] & Fred W. Mast[1]

[1] Institute of Psychology, Universität Bern

Author Note

Add complete departmental affiliations for each author here. Each new line herein must be indented, like this line. Enter author note here.

Correspondence concerning this article should be addressed to Enea J. Weber, Fabrikstrasse 8 - 3012 Bern. E-mail: enea.weber@unibe.ch

Gaze Patterns During Mental Imagery Reflect Part-Based Generation

The Supplementary material contains additional information about the main article.

S1. Stimuli used (Experiment 1 and 2)

Pictures of indoor and outdoor scenes were selected from the FIGRIM (Bylinskii et al., 2015) and LaMem (Khosla et al., 2015) databases. Selected pictures had high memorability scores (>60) to ensure consistency in the difficulty to imagine the pictures within each category. Abstract art pictures were retrieved from the internet and analyzed with ResMem, which is a validated machine learning model for predicting the intrinsic memorability of an image (Davis & Bainbridge, 2023). Again, only pictures with high memorability scores within the prior sample were used for the experiment. None of the selected pictures contained any faces or text. Since many pictures were only available with a low resolution, they were upscaled using the Photo AI software (Topaz Labs, version 3.0.3) to fit the screen with good resolution.

Next, a pretest (n = 18) was conducted online using PsytoolKit (Stoet, 2010, 2017) with all selected pictures. Participants had to both visually inspect and imagine each image for 10 sec and indicate how difficult it was to visually imagine it from 1 (very easy) to 7 (very difficult). All stimuli were presented in a randomized order. After the pretest was completed, responses were analyzed with R (R Core Team, 2023) and Rstudio (RStudio Team, 2020). Low and high percentiles (0.25, 0.75) were calculated within each category. Scores that were lower or higher than those percentiles, were excluded to ensure consistency within each category. Both the mean and median were used for those computations and compared. To get exactly 15 pictures per category, we further excluded those that deviated the most from each category's mean.

S2. Statistical models (Experiment 1)

The following section reports the coefficients of all models for Experiment 1, along with their corresponding model formulas. The posterior predictive checks of the models present in the main article are also reported here. Models for other gaze patterns can also be found in this section.

S2.1. AOI model

We predicted the percentage of fixation per quadrant in imagery by the percentage in the respective quadrant during perception and by the phase (GCW or AS) and the interaction between the two using the following formula:

```
per_imagery/100 ~ 1 + fixations in AOI * phase + AOI + (1 | Participant) + (1 | Stimulus
```

We added the AOI as a predictor to control for systematic biases towards any AOI and used a zero-one-inflated beta regression to fit the data. Fitted coefficients are in table 1. The posterior predictive check can be found in Figure 1

S2.2. MultiMatch models

All MultiMatch models were fitted with Beta regressions, as the similarity scores all lie between 0 and 100.

S2.2.1 Vector similarity models. We predicted the vector similarity using the following formula:

```
Vector ~ comparison * StimType + (1 | Participant) + (1 | Stimulus)
```

The fitted coefficients are in table 2. The posterior predictive check can be found in Figure 2

S2.2.2 Direction similarity models. We predicted the direction similarity using the following formula:

```
Direction ~ comparison * StimType + (1 | Participant) + (1 | Stimulus)
```

The fitted coefficients are in table 3. The posterior predictive check can be found in Figure 3

S2.2.3 Length similarity models. We predicted the length similarity using the following formula:

```
Length ~ comparison * StimType + (1 | Participant) + (1 | Stimulus)
```

The fitted coefficients are in table 4. The posterior predictive check can be found in Figure 4

S2.2.4 Position similarity models. We predicted the position similarity using the following formula:

```
Position ~ comparison * StimType + (1 | Participant) + (1 | Stimulus)
```

The fitted coefficients are in table 5. The posterior predictive check can be found in Figure 5.

S2.2.5 Duration similarity models. We predicted the position similarity using the following formula:

```
Duration ~ comparison * StimType + (1 | Participant) + (1 | Stimulus)
```

The fitted coefficients are in table 6. The posterior predictive check can be found in Figure 6.

S2.3. RQA models

All RQA models were fitted with zero-one-inflated Beta (ZOIB) regressions, because all values were between 0 and 1 included, and we wanted to account for values being exactly 0 and exactly 1. The zero-one inflation (zoi) and the precision (phi) distributional parameters were included in the models because these distribution parameters varied greatly between the different experimental phases. Not including them leads to bad fits. Outcome variables were divided by 100 to make the values compatible with the ZOIB regressions.

S2.3.1 Recurrence. We predicted the recurrence values using the following model formula:

```
Recurrence/100 ~ 1 + Phase * StimType + FixationDispersion + (1 + Phase | Participant) +
     zoi ~ Phase
     phi ~ Phase
```

The fitted coefficients are in table 7. The posterior predictive check can be found in Figure 7.

S2.3.2 Determinism. We predicted the determinism values using the following model formula:

```
Determinism/100 ~ 1 + Phase * StimType + FixationDispersion + (1 + Phase | Participant)
     zoi ~ Phase
     phi ~ Phase
```

The fitted coefficients are in table 8. The posterior predictive check can be found in Figure 8.

S2.3.3. Laminarity. We predicted the determinism values using the following model formula:

```
Laminarity/100 ~ 1 + Phase * StimType + FixationDispersion + (1 + Phase | Participant) +
     zoi ~ Phase
     phi ~ Phase
```

The fitted coefficients are in table 9. The posterior predictive check can be found in Figure 9.

S2.3.4. Center of Recurrence Mass (CORM). We predicted the CORM values using the following model formula:

```
CORM/100 ~ 1 + Phase * StimType + FixationDispersion + (1 + Phase | Participant) + (1 |
     zoi ~ Phase
     phi ~ Phase
```

The fitted coefficients are in table 10.

S2.4. Gaze models

S2.4.1. Number of fixations. We predicted the number of fixations in a poisson-regression using the following model formula:

```
NumFixations ~ Phase * StimType + (1 + StimType | Participant) + (1 | Stimulus)
```

The fitted coefficients are in table 11.

S2.4.2. Fixation durations. We predicted the median duration of fixations in a lognormal regression using the following model formula:

```
MedianDuration ~ Phase * StimType + (1 + StimType | Participant) + (1 | Stimulus)
```

The fitted coefficients are in table 12.

S2.4.3. Fixation spread. We predicted the spread of fixations in a lognormal regression using the following model formula:

```
FixationDispersion + 1e-06 ~ Phase * StimType + (1 + StimType | Participant) + (1 | Stim
```

A small value was added for the 8 out of 13217 trials where the spread was 0 (due to a single fixation recorded during imagery), since lognormal regressions require values greater than 0. The fitted coefficients are in table 13.

S3. Statistical models (Experiment 2)

The following section reports the coefficients of all models for experiment 2, along with their corresponding model formulas. Models are presented in the same order as they appear in the Results section of the main article. The posterior predictive checks of the models present in the main article are also reported here. Models for other gaze patterns can also be found in this section.

S3.1. AOI model

We predicted the percentage of fixation per quadrant in imagery by the percentage in the respective quadrant during perception and by the phase (GCW or AS) and the interaction between the two using the following formula:

```
per_imagery/100 ~ 1 + fixations in AOI * phase + AOI + (1 | Participant) + (1 | Stimulus
```

We added the AOI as a predictor to control for systematic biases towards any AOI and used a zero-one-inflated beta regression to fit the data. Fitted coefficients are in table 14. The posterior predictive check can be found in Figure 10.

S3.2. MultiMatch models

All MultiMatch models were fitted with Beta regressions, as the similarity scores all lie between 0 and 100.

S3.2.1. Vector similarity models. We predicted the vector similarity using the following formula:

```
Vector ~ comparison * StimType + (1 | Participant) + (1 | Stimulus)
```

The fitted coefficients are in table 15. The posterior predictive check can be found in Figure 11.

S3.2.2. Direction similarity models. We predicted the direction similarity using the following formula:

```
Direction ~ comparison * StimType + (1 | Participant) + (1 | Stimulus)
```

The fitted coefficients are in table 16. The posterior predictive check can be found in Figure 12.

S3.2.3. Length similarity models. We predicted the length similarity using the following formula:

```
Length ~ comparison * StimType + (1 | Participant) + (1 | Stimulus)
```

The fitted coefficients are in table 17. The posterior predictive check can be found in Figure 13.

S3.2.4 Position similarity models. We predicted the position similarity using the following formula:

```
Position ~ comparison * StimType + (1 | Participant) + (1 | Stimulus)
```

The fitted coefficients are in table 18. The posterior predictive check can be found in Figure 14.

S3.2.5 Duration similarity models. We predicted the position similarity using the following formula:

```
Duration ~ comparison * StimType + (1 | Participant) + (1 | Stimulus)
```

The fitted coefficients are in table 19. The posterior predictive check can be found in Figure 15.

S3.3. RQA models

All RQA models were fitted with zero-one-inflated Beta (ZOIB) regressions, because all values were between 0 and 1 included, and we wanted to account for values being exactly 0 and exactly 1. The zero-one inflation (zoi) and the precision (phi) distributional parameters were included in the models because these distribution parameters varied greatly between the different experimental phases. Not including them leads to bad fits. Outcome variables were divided by 100 to make the values compatible with the ZOIB regressions.

S3.3.1. Recurrence. We predicted the recurrence values using the following model formula:

```
Recurrence/100 ~ 1 + Phase * StimType + FixationDispersion + (1 + Phase | Participant) +
    zoi ~ Phase
    phi ~ Phase
```

The fitted coefficients are in table 20. The posterior predictive check can be found in Figure 16.

S3.3.2. Determinism. We predicted the determinism values using the following model formula:

```
Determinism/100 ~ 1 + Phase * StimType + FixationDispersion + (1 + Phase | Participant)
    zoi ~ Phase
    phi ~ Phase
```

The fitted coefficients are in table 21. The posterior predictive check can be found in Figure 17.

S3.3.3. Laminarity. We predicted the determinism values using the following model formula:

```
Laminarity/100 ~ 1 + Phase * StimType + FixationDispersion + (1 + Phase | Participant) +
    zoi ~ Phase
    phi ~ Phase
```

The fitted coefficients are in table 22. The posterior predictive check can be found in Figure 18.

S3.3.4. Center of Recurrence Mass (CORM). We predicted the CORM values using the following model formula:

```
CORM/100 ~ 1 + Phase * StimType + FixationDispersion + (1 + Phase | Participant) + (1 |
    zoi ~ Phase
    phi ~ Phase
```

The fitted coefficients are in table 23.

S3.4. Gaze models

S3.4.1. Number of fixations. We predicted the number of fixations in a poisson-regression using the following model formula:

```
NumFixations ~ Phase * StimType + (1 + StimType | Participant) + (1 | Stimulus)
```

The fitted coefficients are in table 24.

S3.4.2. Fixation durations. We predicted the median duration of fixations in a lognormal regression using the following model formula:

```
MedianDuration ~ Phase * StimType + (1 + StimType | Participant) + (1 | Stimulus)
```

The fitted coefficients are in table 25.

S3.4.3. Fixation spread. We predicted the spread of fixations in a lognormal regression using the following model formula:

```
FixationDispersion + 1e-06 ~ Phase * StimType + (1 + StimType | Participant) + (1 | Stim
```

A small value was added for the few trials where the spread was 0 (due to a single fixation recorded during imagery), since lognormal regressions require values greater than 0. The fitted coefficients are in table 26.

S3.4. Bayesian pairwise comparisons

To test for differences between the three mental imagery (MI) conditions, following free perception (FP), gaze-contingent window (GCW), and artificial scotoma (AS), we conducted Bayesian pairwise comparisons. Specifically, we tested:

```
MI-GCW vs. MI-FP
```

```
MI-AS vs. MI-FP
```

```
MI-AS vs. MI-GCW
```

These comparisons were performed separately for both determinism and laminarity, based on the posterior samples from the fitted Bayesian models (det_exp2 and lam_exp2).

Pairwise comparisons between MI conditions were conducted using the `hypothesis()` function in **brms**. The resulting estimates and 95% credible intervals are reported in Tables 27 (determinism) and 28 (laminarity).

S4. Probe accuracy and vividness ratings Experiment 1

S4.1. Probe Accuracy (Experiment 1)

A summary about the accuracy in answering the probe questions can be found in Table 29.

S4.2. Vividness ratings (Experiment 1)

A summary of the trial-wise vividness ratings can be found in Table 30.

S5. Probe accuracy and vividness ratings Experiment 2

S5.1. Probe accuracy (Experiment 2)

A summary about the accuracy in answering the probe questions can be found in Table 31.

S5.2. Vividness ratings (Experiment 2)

A summary of the trial-wise vividness ratings can be found in Table 32.

S6. Sample information

This section shows descriptive statistics of questionnaires and tasks completed by our participants for both Experiments. The questionnaires and tasks were collected for exploratory purposes.

S6.1. Experiment 1

S6.1.1 VVIQ (Experiment 1). We assessed the Vividness of Visual Imagery Questionnaire 2 (VVIQ 2, Marks, 1995), which assesses individual abilities to generate vivid mental images. An overview of the distribution can be found in Figure 19. The average VVIQ score accross all participants in Experiment 1 was 3.46 (out of 5).

S6.1.2 OSIVQ (Experiment 1). We assessed the Object-Spatial Imagery and Verbal Questionnaire (OSIVQ, Blazhenkova & Kozhevnikov, 2009), which asses individual cognitive styles for imagery. The mean scores from all participants for each factor are illustrated in Figure 20, and for a summary table, check Table 33.

S6.1.3 Mental Rotation (Experiment 1). We assessed mental rotation performance with Vanderberg's task (Vandenberg & Kuse, 1978). The distribution from total scores are illustrated in Figure 21. The average total score across all participants is 22.31.

S6.1.4 Working Memory (Experiment 1). We assessed Working Memory with an N-Back task coded in MATLAB. The n-back task consisted of 2 training blocks (2 and 3-n-back), followed by 6 experimental blocks. Each block consisted of 20 trials. During the task, a circle appeared every 4 sec on a random position on a 3x3 grid, and participants had to press "yes" or "no" whether the circle appeared on the same position as n steps before. Each block had exactly 5 correct trials (i.e. where the correct answer was "yes") to ensure consistent difficulty between participants. The task always started with the 2-n-back condition, and the difficulty varied depending on the percentage of correct responses in each block. If the block accuracy was 70% or below, the n level decreased by one, but not below 2-n-back. If the block accuracy was 90% or above, the n level increased by one, and remained unchanged if the accuracy was between the two thresholds. The average score across all participants was 2.88. The distribution of these scores can be found in Figure 22

S6.2. Experiment 2

S6.2.1 VVIQ (Experiment 2). We assessed the Vividness of Visual Imagery Questionnaire 3 (VVIQ 2, Marks, 1995), which assesses individual abilities to generate vivid mental images. An overview of the distribution can be found in Figure 23. The average VVIQ score accross all participants in Experiment 1 was 3.48 (out of 5).

S6.2.2 OSIVQ (Experiment 2). We assessed the Object-Spatial Imagery and Verbal Questionnaire (OSIVQ, Blazhenkova & Kozhevnikov, 2009), which asses individual cognitive styles for imagery. The mean scores from all participants for each factor are illustrated in Figure 24, and for a summary table, check Table 34.

S6.2.3 Mental Rotation (Experiment 2). We assessed mental rotation performance with Vanderberg's task (Vandenberg & Kuse, 1978). The distribution from total scores are illustrated in Figure 25. The average total score across all participants is 24.1.

S6.2.4 Working Memory (Experiment 2). We assessed Working Memory with an N-Back task coded in MATLAB. The scores for each participant were the defined as n-level reached in the final block, weighted by each participant's accuracy in that block (e.g., 90% correct). The average score across all participants was 3.13. The distribution of these scores can be found in Figure 26

S7. Eye-tracking data check

We investigated gaze patterns from each participant in relation to other participants, to detect outliers in gaze behavior and potential technical problems.

S7.1 Experiment 1

Participants that were excluded and reason:

- g17: too much time where the eye-tracker was not tracking
- g21: very few and extremely long fixations
- g41: very few and long fixations

Plots showing gaze patterns from each participant in relation to other participants can be found in *experiment_1/analyses/Participant_Plots.pdf*

S7.2 Experiment 2

Participants that were excluded and reason:

- vp11: excluded because the eye-tracker did not work
- vp14: short fixations, and many fixations outside the screen
- vp27: excluded because of eye-tracking calibration issues
- vp45: excluded because of aphantasia
- vp54: very few, very long fixations

Plots showing gaze patterns from each participant in relation to other participants can be found in *experiment_2/analyses/Participant_Plots.pdf*

Aknowledgements

We thank Lorena Di Matteo, Luca Panico and Noam Sedemund for their help during data collection.

This research was supported by the Swiss National Science Foundation, grant no. 100014_214940 (PI: FWM).

References

Blazhenkova, O., & Kozhevnikov, M. (2009). The new object–spatial–verbal cognitive style model: Theory and measurement. *Applied Cognitive Psychology, 23*(5), 638–663. https://doi.org/10.1002/acp.1473

Bylinskii, Z., Isola, P., Bainbridge, C., Torralba, A., & Oliva, A. (2015). Intrinsic and extrinsic effects on image memorability. *Vision Research, 116*, 165–178. https://doi.org/10.1016/j.visres.2015.03.005

Davis, T. M., & Bainbridge, W. A. (2023). Memory for artwork is predictable. *Proceedings of the National Academy of Sciences, 120*(28), e2302389120. https://doi.org/10.1073/pnas.2302389120

Khosla, A., Raju, A. S., Torralba, A., & Oliva, A. (2015). Understanding and predicting image memorability at a large scale. In *Proceedings of the IEEE International Conference on Computer Vision (ICCV)* (pp. 2390–2398). https://openaccess.thecvf.com/content_iccv_2015/html/Khosla_Understanding_and_Predicting_ICCV_2015_paper.html

Marks, D. F. (1995). New directions for mental imagery research. *Journal of Mental Imagery, 19*(3–4), 153–167.

R Core Team. (2023). *R: A language and environment for statistical computing* [Software]. R Foundation for Statistical Computing. https://www.R-project.org/

RStudio Team. (2020). *RStudio: Integrated development environment for R* [Software]. RStudio, PBC. https://www.rstudio.com/

Stoet, G. (2010). PsyToolkit: A software package for programming psychological experiments using Linux. *Behavior Research Methods, 42*(4), 1096–1104. https://doi.org/10.3758/BRM.42.4.1096

Stoet, G. (2017). PsyToolkit: A novel web-based method for running online

questionnaires and reaction-time experiments. *Teaching of Psychology, 44*(1), 24–31. https://doi.org/10.1177/0098628316677643

Topaz Labs. (n.d.). *Topaz Labs* (Version 3.0.3) [Software]. https://www.topazlabs.com

Vandenberg, S. G., & Kuse, A. R. (1978). Mental rotations, a group test of three-dimensional spatial visualization. *Perceptual and Motor Skills, 47*(2), 599–604. https://doi.org/10.2466/pms.1978.47.2.599

Table 1

predictors	estimate	lower CI	upper CI
Intercept	-0.99	-1.03	-0.94
fixations in AOI	0.02	0.02	0.02
phase_GCW	0.04	-0.02	0.10
phase_AS	0.03	-0.03	0.08
AOI 2	-0.16	-0.18	-0.13
AOI 3	-0.69	-0.72	-0.66
AOI 4	-0.74	-0.77	-0.71
fixations in AOI : GCW	0.00	0.00	0.00
fixations in AOI : AS	0.00	0.00	0.00

Table 2

predictors	estimate	lower CI	upper CI
Intercept	2.98	2.90	3.05
Comparison_FP - GCW	0.14	0.12	0.17
Comparison_FP - AS	-0.15	-0.18	-0.13
Comparison_Imagery - GCW	0.35	0.32	0.38
Comparison_Imagery - AS	-0.24	-0.26	-0.22
StimType_Indoor	0.11	0.06	0.16
StimType_Outdoor	0.03	-0.02	0.08
Comparison_FP - GCW : StimType_Indoor	-0.01	-0.04	0.03
Comparison_FP - AS : StimType_Indoor	-0.05	-0.08	-0.02
Comparison_Imagery - GCW : StimType_Indoor	-0.05	-0.09	-0.01
Comparison_Imagery - AS : StimType_Indoor	-0.05	-0.08	-0.02
Comparison_FP - GCW : StimType_Outdoor	0.03	-0.01	0.06
Comparison_FP - AS : StimType_Outdoor	-0.03	-0.06	0.00
Comparison_Imagery - GCW : StimType_Outdoor	-0.02	-0.06	0.02
Comparison_Imagery - AS : StimType_Outdoor	-0.08	-0.11	-0.04

Table 3

predictors	estimate	lower CI	upper CI
Intercept	1.21	1.15	1.28
Comparison_FP - GCW	0.12	0.09	0.15
Comparison_FP - AS	0.14	0.11	0.17
Comparison_Imagery - GCW	0.08	0.05	0.12
Comparison_Imagery - AS	-0.02	-0.05	0.01
StimType_Indoor	-0.03	-0.10	0.03
StimType_Outdoor	0.06	-0.01	0.13
Comparison_FP - GCW : StimType_Indoor	0.01	-0.04	0.05
Comparison_FP - AS : StimType_Indoor	-0.04	-0.08	0.01
Comparison_Imagery - GCW : StimType_Indoor	0.04	0.00	0.09
Comparison_Imagery - AS : StimType_Indoor	0.04	0.00	0.09
Comparison_FP - GCW : StimType_Outdoor	0.08	0.04	0.13
Comparison_FP - AS : StimType_Outdoor	0.08	0.04	0.13
Comparison_Imagery - GCW : StimType_Outdoor	0.05	0.01	0.10
Comparison_Imagery - AS : StimType_Outdoor	0.04	0.00	0.09

Table 4

predictors	estimate	lower CI	upper CI
Intercept	2.70	2.61	2.78
Comparison_FP - GCW	0.31	0.28	0.34
Comparison_FP - AS	-0.01	-0.04	0.02
Comparison_Imagery - GCW	0.43	0.40	0.47
Comparison_Imagery - AS	-0.31	-0.34	-0.28
StimType_Indoor	0.12	0.06	0.19
StimType_Outdoor	0.02	-0.04	0.09
Comparison_FP - GCW : StimType_Indoor	-0.05	-0.09	0.00
Comparison_FP - AS : StimType_Indoor	-0.11	-0.16	-0.07
Comparison_Imagery - GCW : StimType_Indoor	-0.06	-0.11	-0.01
Comparison_Imagery - AS : StimType_Indoor	-0.06	-0.10	-0.02
Comparison_FP - GCW : StimType_Outdoor	0.01	-0.04	0.05
Comparison_FP - AS : StimType_Outdoor	-0.09	-0.13	-0.05
Comparison_Imagery - GCW : StimType_Outdoor	-0.04	-0.08	0.01
Comparison_Imagery - AS : StimType_Outdoor	-0.10	-0.14	-0.06

Table 5

predictors	estimate	lower CI	upper CI
Intercept	1.49	1.43	1.54
Comparison_FP - GCW	-0.22	-0.24	-0.19
Comparison_FP - AS	-0.07	-0.10	-0.04
Comparison_Imagery - GCW	-0.26	-0.28	-0.23
Comparison_Imagery - AS	-0.08	-0.11	-0.05
StimType_Indoor	-0.13	-0.18	-0.07
StimType_Outdoor	-0.01	-0.07	0.05
Comparison_FP - GCW : StimType_Indoor	0.14	0.10	0.18
Comparison_FP - AS : StimType_Indoor	0.02	-0.02	0.06
Comparison_Imagery - GCW : StimType_Indoor	0.15	0.11	0.19
Comparison_Imagery - AS : StimType_Indoor	0.05	0.01	0.09
Comparison_FP - GCW : StimType_Outdoor	0.02	-0.02	0.06
Comparison_FP - AS : StimType_Outdoor	0.00	-0.04	0.04
Comparison_Imagery - GCW : StimType_Outdoor	0.01	-0.03	0.05
Comparison_Imagery - AS : StimType_Outdoor	0.01	-0.03	0.05

Table 6

predictors	estimate	lower CI	upper CI
Intercept	0.01	-0.08	0.10
Comparison_FP - GCW	0.65	0.61	0.69
Comparison_FP - AS	0.53	0.49	0.58
Comparison_Imagery - GCW	-0.01	-0.06	0.03
Comparison_Imagery - AS	-0.04	-0.08	0.00
StimType_Indoor	-0.06	-0.11	-0.02
StimType_Outdoor	-0.03	-0.07	0.01
Comparison_FP - GCW : StimType_Indoor	0.07	0.01	0.14
Comparison_FP - AS : StimType_Indoor	-0.01	-0.07	0.05
Comparison_Imagery - GCW : StimType_Indoor	0.04	-0.01	0.10
Comparison_Imagery - AS : StimType_Indoor	0.03	-0.03	0.09
Comparison_FP - GCW : StimType_Outdoor	0.04	-0.03	0.10
Comparison_FP - AS : StimType_Outdoor	0.02	-0.04	0.08
Comparison_Imagery - GCW : StimType_Outdoor	0.00	-0.06	0.06
Comparison_Imagery - AS : StimType_Outdoor	-0.02	-0.08	0.04

Table 7

predictors	estimate	lower CI	upper CI
Intercept	-0.81	-0.89	-0.74
phi_Intercept	4.41	4.37	4.46
zoi_Intercept	-11.50	-19.70	-7.63
Phase_Imagery	0.86	0.69	1.03
Phase_GCW	-0.49	-0.54	-0.44
Phase_AS	-0.23	-0.31	-0.16
StimType_Indoor	0.13	0.06	0.20
StimType_Outdoor	-0.01	-0.09	0.06
Fixation Dispersion	0.00	0.00	0.00
Phase_Imagery : StimType_Indoor	-0.26	-0.31	-0.20
Phase_GCW : StimType_Indoor	0.05	0.01	0.08
Phase_AS : StimType_Indoor	0.01	-0.04	0.06
Phase_Imagery : StimType_Outdoor	-0.02	-0.08	0.04
Phase_GCW : StimType_Outdoor	0.14	0.10	0.18
Phase_AS : StimType_Outdoor	0.05	0.00	0.10
phi_Imagery	-2.15	-2.21	-2.09
phi_GCW	1.27	1.20	1.35
phi_AS	-0.04	-0.12	0.03
zoi_Imagery	7.85	3.97	16.10
zoi_GCW	-2.40	-19.60	8.67
zoi_AS	5.86	1.90	14.20

Table 8

predictors	estimate	lower CI	upper CI
Intercept	0.25	0.13	0.37
phi_Intercept	2.55	2.51	2.59
zoi_Intercept	-6.45	-7.24	-5.78
Phase_Imagery	0.73	0.55	0.91
Phase_GCW	0.46	0.34	0.58
Phase_AS	-0.38	-0.48	-0.27
StimType_Indoor	0.34	0.26	0.41
StimType_Outdoor	0.08	0.00	0.15
Fixation Dispersion	0.00	0.00	0.00
Phase_Imagery : StimType_Indoor	-0.32	-0.39	-0.26
Phase_GCW : StimType_Indoor	-0.10	-0.17	-0.04
Phase_AS : StimType_Indoor	-0.06	-0.14	0.01
Phase_Imagery : StimType_Outdoor	-0.09	-0.15	-0.02
Phase_GCW : StimType_Outdoor	0.01	-0.05	0.08
Phase_AS : StimType_Outdoor	0.02	-0.06	0.09
phi_Imagery	-0.62	-0.68	-0.56
phi_GCW	0.23	0.15	0.30
phi_AS	0.08	0.01	0.15
zoi_Imagery	2.86	2.16	3.67
zoi_GCW	-0.71	-2.55	0.75
zoi_AS	2.80	2.06	3.64

Table 9

predictors	estimate	lower CI	upper CI
Intercept	0.57	0.45	0.69
phi_Intercept	2.62	2.58	2.66
zoi_Intercept	-6.81	-7.78	-6.02
Phase_Imagery	0.37	0.22	0.53
Phase_GCW	0.01	-0.08	0.11
Phase_AS	-0.78	-0.89	-0.68
StimType_Indoor	0.46	0.37	0.55
StimType_Outdoor	0.09	0.00	0.18
Fixation Dispersion	0.00	0.00	0.00
Phase_Imagery : StimType_Indoor	-0.47	-0.54	-0.41
Phase_GCW : StimType_Indoor	-0.14	-0.20	-0.08
Phase_AS : StimType_Indoor	-0.28	-0.36	-0.21
Phase_Imagery : StimType_Outdoor	-0.13	-0.19	-0.07
Phase_GCW : StimType_Outdoor	0.08	0.02	0.14
Phase_AS : StimType_Outdoor	-0.19	-0.27	-0.11
phi_Imagery	-0.28	-0.34	-0.22
phi_GCW	0.57	0.50	0.64
phi_AS	-0.04	-0.12	0.03
zoi_Imagery	2.77	1.95	3.77
zoi_GCW	-1.20	-3.97	0.82
zoi_AS	3.22	2.37	4.21

Table 10

predictors	estimate	lower CI	upper CI
Intercept	-0.61	-0.66	-0.56
phi_Intercept	4.20	4.16	4.24
zoi_Intercept	-6.44	-7.26	-5.77
Phase_Imagery	-0.10	-0.14	-0.05
Phase_GCW	-0.06	-0.12	0.01
Phase_AS	0.13	0.09	0.18
StimType_Indoor	-0.05	-0.08	-0.01
StimType_Outdoor	-0.01	-0.05	0.02
Fixation Dispersion	0.00	0.00	0.00
Phase_Imagery : StimType_Indoor	0.00	-0.03	0.03
Phase_GCW : StimType_Indoor	0.00	-0.04	0.04
Phase_AS : StimType_Indoor	0.02	-0.02	0.05
Phase_Imagery : StimType_Outdoor	0.00	-0.03	0.03
Phase_GCW : StimType_Outdoor	0.06	0.02	0.10
Phase_AS : StimType_Outdoor	0.02	-0.01	0.06
phi_Imagery	-0.39	-0.46	-0.33
phi_GCW	-0.50	-0.57	-0.42
phi_AS	0.07	-0.01	0.14
zoi_Imagery	2.67	1.96	3.51
zoi_GCW	-0.71	-2.58	0.80
zoi_AS	2.77	2.04	3.63

Table 11

predictors	estimate	lower CI	upper CI
Intercept	3.93	3.88	3.98
Phase_Imagery	-0.58	-0.59	-0.57
Phase_GCW	0.15	0.14	0.16
Phase_AS	-0.07	-0.08	-0.05
StimType_Indoor	0.04	0.01	0.06
StimType_Outdoor	0.00	-0.02	0.02
Phase_Imagery : StimType_Indoor	-0.03	-0.04	-0.01
Phase_GCW : StimType_Indoor	-0.05	-0.07	-0.04
Phase_AS : StimType_Indoor	-0.08	-0.10	-0.06
Phase_Imagery : StimType_Outdoor	-0.01	-0.02	0.01
GCW : StimType_Outdoor	0.02	0.00	0.03
Phase_AS : StimType_Outdoor	0.00	-0.02	0.02

Table 12

predictors	estimate	lower CI	upper CI
Intercept	5.60	5.54	5.65
Phase_Imagery	0.52	0.50	0.54
Phase_GCW	-0.11	-0.14	-0.08
Phase_AS	0.05	0.02	0.08
StimType_Indoor	-0.03	-0.06	-0.01
StimType_Outdoor	0.01	-0.01	0.04
Phase_Imagery : StimType_Indoor	0.06	0.03	0.09
Phase_GCW : StimType_Indoor	0.04	0.00	0.08
Phase_AS : StimType_Indoor	0.08	0.04	0.12
Phase_Imagery : StimType_Outdoor	0.03	0.00	0.06
GCW : StimType_Outdoor	-0.04	-0.08	0.00
Phase_AS : StimType_Outdoor	-0.02	-0.06	0.02

Table 13

predictors	estimate	lower CI	upper CI
Intercept	5.86	5.76	5.97
Phase_Imagery	-0.81	-0.86	-0.76
Phase_GCW	0.24	0.18	0.30
Phase_AS	0.05	-0.01	0.11
StimType_Indoor	0.15	0.08	0.23
StimType_Outdoor	0.03	-0.05	0.10
Phase_Imagery : StimType_Indoor	-0.03	-0.10	0.04
Phase_GCW : StimType_Indoor	-0.16	-0.24	-0.08
Phase_AS : StimType_Indoor	-0.09	-0.18	-0.01
Phase_Imagery : StimType_Outdoor	0.01	-0.06	0.08
GCW : StimType_Outdoor	0.00	-0.09	0.08
Phase_AS : StimType_Outdoor	0.00	-0.08	0.09

Table 14

predictors	estimate	lower CI	upper CI
Intercept	-1.32	-1.42	-1.22
fixations in AOI	0.02	0.02	0.03
phase_GCW	0.05	-0.08	0.18
phase_AS	0.03	-0.09	0.15
AOI 2	0.06	0.01	0.11
AOI 3	-0.42	-0.48	-0.36
AOI 4	-0.45	-0.51	-0.38
fixations in AOI : GCW	0.00	-0.01	0.00
fixations in AOI : AS	0.00	-0.01	0.00

Table 15

predictors	estimate	lower CI	upper CI
Intercept	2.89	2.82	2.96
Comparison_Imagery - GCW	0.46	0.42	0.51
Comparison_Imagery - AS	-0.19	-0.23	-0.15
StimType_Indoor	0.14	0.09	0.18
StimType_Outdoor	0.03	-0.02	0.07
Comparison_Imagery - GCW : StimType_Indoor	-0.04	-0.11	0.03
Comparison_Imagery - AS : StimType_Indoor	-0.09	-0.15	-0.03
Comparison_Imagery - GCW : StimType_Outdoor	-0.02	-0.09	0.05
Comparison_Imagery - AS : StimType_Outdoor	-0.11	-0.17	-0.05

Table 16

predictors	estimate	lower CI	upper CI
Intercept	1.29	1.22	1.36
Comparison_Imagery - GCW	0.14	0.08	0.21
Comparison_Imagery - AS	-0.02	-0.08	0.05
StimType_Indoor	-0.07	-0.13	-0.01
StimType_Outdoor	0.07	0.01	0.13
Comparison_Imagery - GCW : StimType_Indoor	0.12	0.03	0.21
Comparison_Imagery - AS : StimType_Indoor	0.08	-0.01	0.17
Comparison_Imagery - GCW : StimType_Outdoor	0.08	-0.01	0.17
Comparison_Imagery - AS : StimType_Outdoor	0.00	-0.09	0.09

Table 17

predictors	estimate	lower CI	upper CI
Intercept	2.58	2.48	2.68
Comparison_Imagery - GCW	0.57	0.50	0.64
Comparison_Imagery - AS	-0.23	-0.28	-0.17
StimType_Indoor	0.16	0.10	0.22
StimType_Outdoor	0.03	-0.03	0.09
Comparison_Imagery - GCW : StimType_Indoor	-0.06	-0.15	0.04
Comparison_Imagery - AS : StimType_Indoor	-0.15	-0.23	-0.07
Comparison_Imagery - GCW : StimType_Outdoor	-0.07	-0.17	0.02
Comparison_Imagery - AS : StimType_Outdoor	-0.18	-0.26	-0.10

Table 18

predictors	estimate	lower CI	upper CI
Intercept	1.48	1.43	1.53
Comparison_Imagery - GCW	-0.29	-0.34	-0.24
Comparison_Imagery - AS	-0.10	-0.15	-0.06
StimType_Indoor	-0.12	-0.16	-0.07
StimType_Outdoor	-0.06	-0.11	-0.01
Comparison_Imagery - GCW : StimType_Indoor	0.15	0.08	0.21
Comparison_Imagery - AS : StimType_Indoor	0.03	-0.03	0.10
Comparison_Imagery - GCW : StimType_Outdoor	0.10	0.03	0.16
Comparison_Imagery - AS : StimType_Outdoor	0.07	0.00	0.14

Table 19

predictors	estimate	lower CI	upper CI
Intercept	-0.07	-0.19	0.05
Comparison_Imagery - GCW	0.07	0.00	0.15
Comparison_Imagery - AS	0.05	-0.03	0.13
StimType_Indoor	0.06	-0.02	0.14
StimType_Outdoor	0.10	0.02	0.18
Comparison_Imagery - GCW : StimType_Indoor	-0.09	-0.20	0.02
Comparison_Imagery - AS : StimType_Indoor	-0.14	-0.25	-0.03
Comparison_Imagery - GCW : StimType_Outdoor	-0.15	-0.26	-0.04
Comparison_Imagery - AS : StimType_Outdoor	-0.16	-0.27	-0.04

Table 20

predictors	estimate	lower CI	upper CI
Intercept	-1.11	-1.23	-0.99
phi_Intercept	4.91	4.80	5.01
zoi_Intercept	-12.90	-29.30	-6.33
Phase_MI_FP	1.11	0.91	1.31
Phase_MI_GCW	1.07	0.84	1.30
Phase_MI_AS	1.05	0.80	1.30
Phase_GCW	-0.35	-0.49	-0.21
Phase_AS	0.02	-0.13	0.16
StimType_Indoor	0.21	0.07	0.35
StimType_Outdoor	0.00	-0.15	0.14
Fixation Dispersion	0.00	0.00	0.00
Phase_MI_FP : StimType_Indoor	-0.32	-0.45	-0.20
Phase_MI_GCW : StimType_Indoor	-0.30	-0.53	-0.08
Phase_MI_AS : StimType_Indoor	-0.15	-0.37	0.08
Phase_GCW : StimType_Indoor	-0.08	-0.28	0.12
Phase_AS : StimType_Indoor	-0.16	-0.36	0.03
Phase_MI_FP : StimType_Outdoor	-0.15	-0.27	-0.02
Phase_MI_GCW : StimType_Outdoor	0.04	-0.18	0.26
Phase_MI_AS : StimType_Outdoor	0.11	-0.11	0.34
Phase_GCW : StimType_Outdoor	0.07	-0.13	0.26
Phase_AS : StimType_Outdoor	-0.05	-0.24	0.15
phi_MI_FP	-2.49	-2.64	-2.34
phi_MI_GCW	-2.54	-2.69	-2.38
phi_MI_AS	-2.61	-2.76	-2.45
phi_GCW	1.37	1.22	1.52
phi_AS	0.06	-0.09	0.21
zoi_MI_FP	9.38	2.84	25.80

Table 21

predictors	estimate	lower CI	upper CI
Intercept	0.05	-0.12	0.22
phi_Intercept	2.75	2.65	2.85
zoi_Intercept	-6.10	-7.84	-4.83
Phase_MI_FP	0.97	0.76	1.17
Phase_MI_GCW	1.02	0.78	1.27
Phase_MI_AS	0.92	0.66	1.19
Phase_GCW	0.76	0.56	0.95
Phase_AS	0.11	-0.06	0.29
StimType_Indoor	0.59	0.42	0.75
StimType_Outdoor	0.22	0.06	0.40
Fixation Dispersion	0.00	0.00	0.00
Phase_MI_FP : StimType_Indoor	-0.58	-0.74	-0.43
Phase_MI_GCW : StimType_Indoor	-0.63	-0.88	-0.39
Phase_MI_AS : StimType_Indoor	-0.36	-0.61	-0.11
Phase_GCW : StimType_Indoor	-0.34	-0.57	-0.10
Phase_AS : StimType_Indoor	-0.42	-0.65	-0.18
Phase_MI_FP : StimType_Outdoor	-0.35	-0.51	-0.20
Phase_MI_GCW : StimType_Outdoor	-0.24	-0.48	0.01
Phase_MI_AS : StimType_Outdoor	-0.09	-0.34	0.15
Phase_GCW : StimType_Outdoor	-0.27	-0.50	-0.04
Phase_AS : StimType_Outdoor	-0.29	-0.52	-0.05
phi_MI_FP	-0.65	-0.80	-0.50
phi_MI_GCW	-0.55	-0.69	-0.40
phi_MI_AS	-0.57	-0.72	-0.42
phi_GCW	0.21	0.06	0.36
phi_AS	0.26	0.11	0.40
zoi_MI_FP	2.32	0.95	4.12

Table 22

predictors	estimate	lower CI	upper CI
Intercept	0.42	0.24	0.60
phi_Intercept	2.68	2.58	2.79
zoi_Intercept	-6.94	-9.60	-5.23
Phase_MI_FP	0.62	0.44	0.81
Phase_MI_GCW	0.70	0.46	0.93
Phase_MI_AS	0.60	0.36	0.84
Phase_GCW	0.42	0.22	0.62
Phase_AS	-0.53	-0.75	-0.31
StimType_Indoor	0.70	0.53	0.88
StimType_Outdoor	0.19	0.02	0.37
Fixation Dispersion	0.00	0.00	0.00
Phase_MI_FP : StimType_Indoor	-0.72	-0.86	-0.58
Phase_MI_GCW : StimType_Indoor	-0.76	-1.01	-0.52
Phase_MI_AS : StimType_Indoor	-0.54	-0.79	-0.30
Phase_GCW : StimType_Indoor	-0.47	-0.71	-0.23
Phase_AS : StimType_Indoor	-0.61	-0.85	-0.37
Phase_MI_FP : StimType_Outdoor	-0.30	-0.44	-0.16
Phase_MI_GCW : StimType_Outdoor	-0.22	-0.47	0.03
Phase_MI_AS : StimType_Outdoor	-0.17	-0.42	0.08
Phase_GCW : StimType_Outdoor	-0.16	-0.40	0.08
Phase_AS : StimType_Outdoor	-0.45	-0.71	-0.20
phi_MI_FP	-0.11	-0.27	0.04
phi_MI_GCW	-0.07	-0.22	0.08
phi_MI_AS	-0.04	-0.19	0.11
phi_GCW	0.79	0.64	0.94
phi_AS	0.23	0.08	0.37
zoi_MI_FP	2.79	0.96	5.53

Table 23

predictors	estimate	lower CI	upper CI
Intercept	-0.51	-0.57	-0.44
phi_Intercept	4.53	4.43	4.64
zoi_Intercept	-6.10	-7.81	-4.88
Phase_MI_FP	-0.08	-0.15	-0.02
Phase_MI_GCW	-0.16	-0.24	-0.08
Phase_MI_AS	-0.12	-0.20	-0.04
Phase_GCW	0.08	0.00	0.16
Phase_AS	0.10	0.02	0.17
StimType_Indoor	-0.04	-0.11	0.03
StimType_Outdoor	-0.02	-0.09	0.05
Fixation Dispersion	0.00	0.00	0.00
Phase_MI_FP : StimType_Indoor	-0.01	-0.08	0.05
Phase_MI_GCW : StimType_Indoor	0.01	-0.09	0.11
Phase_MI_AS : StimType_Indoor	0.01	-0.09	0.11
Phase_GCW : StimType_Indoor	-0.16	-0.26	-0.07
Phase_AS : StimType_Indoor	0.01	-0.09	0.10
Phase_MI_FP : StimType_Outdoor	-0.02	-0.09	0.04
Phase_MI_GCW : StimType_Outdoor	0.03	-0.07	0.13
Phase_MI_AS : StimType_Outdoor	0.01	-0.09	0.11
Phase_GCW : StimType_Outdoor	0.03	-0.07	0.12
Phase_AS : StimType_Outdoor	-0.02	-0.11	0.08
phi_MI_FP	-0.48	-0.63	-0.33
phi_MI_GCW	-0.49	-0.64	-0.34
phi_MI_AS	-0.28	-0.44	-0.13
phi_GCW	-0.38	-0.53	-0.22
phi_AS	0.11	-0.04	0.26
zoi_MI_FP	2.19	0.85	3.95

Table 24

predictors	estimate	lower CI	upper CI
Intercept	4.05	4.00	4.10
Phase_MI_FP	-0.67	-0.77	-0.57
Phase_MI_GCW	-0.55	-0.65	-0.46
Phase_MI_AS	-0.68	-0.80	-0.57
Phase_GCW	0.16	0.10	0.21
Phase_AS	0.01	-0.04	0.07
StimType_Indoor	0.03	-0.01	0.06
StimType_Outdoor	-0.02	-0.06	0.02
Phase_MI_FP : StimType_Indoor	0.01	-0.02	0.05
Phase_MI_GCW : StimType_Indoor	-0.04	-0.10	0.01
Phase_MI_AS : StimType_Indoor	-0.03	-0.09	0.02
Phase_GCW : StimType_Indoor	-0.04	-0.09	0.01
Phase_AS : StimType_Indoor	-0.03	-0.08	0.02
Phase_MI_FP : StimType_Outdoor	0.05	0.01	0.09
Phase_MI_GCW : StimType_Outdoor	-0.02	-0.08	0.03
Phase_MI_AS : StimType_Outdoor	0.02	-0.04	0.07
Phase_GCW : StimType_Outdoor	0.05	0.00	0.10
Phase_AS : StimType_Outdoor	0.05	-0.01	0.10

Table 25

predictors	estimate	lower CI	upper CI
Intercept	5.49	5.45	5.53
Phase_MI_FP	0.58	0.49	0.68
Phase_MI_GCW	0.46	0.37	0.56
Phase_MI_AS	0.56	0.46	0.67
Phase_GCW	-0.09	-0.14	-0.04
Phase_AS	-0.02	-0.08	0.03
StimType_Indoor	-0.01	-0.06	0.03
StimType_Outdoor	0.04	-0.01	0.08
Phase_MI_FP : StimType_Indoor	0.00	-0.05	0.06
Phase_MI_GCW : StimType_Indoor	0.05	-0.01	0.12
Phase_MI_AS : StimType_Indoor	0.09	0.03	0.16
Phase_GCW : StimType_Indoor	0.02	-0.05	0.08
Phase_AS : StimType_Indoor	0.01	-0.06	0.07
Phase_MI_FP : StimType_Outdoor	-0.06	-0.12	0.00
Phase_MI_GCW : StimType_Outdoor	0.02	-0.04	0.08
Phase_MI_AS : StimType_Outdoor	0.02	-0.05	0.08
Phase_GCW : StimType_Outdoor	-0.07	-0.13	0.00
Phase_AS : StimType_Outdoor	-0.06	-0.13	0.00

Table 26

predictors	estimate	lower CI	upper CI
Intercept	5.91	5.78	6.03
Phase_MI_FP	-0.95	-1.15	-0.76
Phase_MI_GCW	-0.87	-1.09	-0.66
Phase_MI_AS	-1.06	-1.36	-0.78
Phase_GCW	0.28	0.11	0.45
Phase_AS	0.11	-0.06	0.28
StimType_Indoor	0.14	-0.02	0.31
StimType_Outdoor	0.07	-0.10	0.23
Phase_MI_FP : StimType_Indoor	0.00	-0.18	0.19
Phase_MI_GCW : StimType_Indoor	-0.03	-0.27	0.21
Phase_MI_AS : StimType_Indoor	0.00	-0.24	0.23
Phase_GCW : StimType_Indoor	-0.14	-0.38	0.10
Phase_AS : StimType_Indoor	-0.10	-0.34	0.14
Phase_MI_FP : StimType_Outdoor	0.04	-0.14	0.22
Phase_MI_GCW : StimType_Outdoor	-0.09	-0.33	0.16
Phase_MI_AS : StimType_Outdoor	-0.21	-0.45	0.03
Phase_GCW : StimType_Outdoor	-0.09	-0.34	0.15
Phase_AS : StimType_Outdoor	-0.07	-0.30	0.17

Table 27

term	estimate	lower.CI	upper.CI
(PhaseMI_GCW-PhaseMI_FP) = 0	0.05	-0.15	0.26
(PhaseMI_Scotoma-PhaseMI_FP) = 0	-0.05	-0.26	0.16
(PhaseMI_Scotoma-PhaseMI_GCW) = 0	-0.10	-0.31	0.11

Table 28

term	estimate	lower.CI	upper.CI
(PhaseMI_GCW-PhaseMI_FP) = 0	0.07	-0.12	0.27
(PhaseMI_Scotoma-PhaseMI_FP) = 0	-0.02	-0.22	0.17
(PhaseMI_Scotoma-PhaseMI_GCW) = 0	-0.10	-0.29	0.09

Table 29

Mean (SD) accuracy of probe responses by stimulus type and condition.

stim_type	condition	mean_accuracy	sd_accuracy
abstract	GCW	0.59	0.49
abstract	rGCW	0.75	0.44
indoor	GCW	0.79	0.40
indoor	rGCW	0.74	0.44
outdoor	GCW	0.69	0.46
outdoor	rGCW	0.61	0.49
Overall		0.70	0.46

Table 30

Mean (SD) vividness ratings by stimulus type and condition.

stim_type	condition	mean_vividness	sd_vividness
abstract	GCW	3.04	1.21
abstract	rGCW	3.00	1.13
indoor	GCW	4.64	1.12
indoor	rGCW	4.47	1.13
outdoor	GCW	4.73	1.12
outdoor	rGCW	4.58	1.13
Overall		4.07	1.36

Table 31

Mean (SD) accuracy of probe responses by stimulus type and condition.

stim_type	condition	mean_accuracy	sd_accuracy
abstract	FP	0.67	0.47
abstract	GCW	0.65	0.48
abstract	Scotoma	0.70	0.46
indoor	FP	0.76	0.43
indoor	GCW	0.53	0.50
indoor	Scotoma	0.64	0.48
outdoor	FP	0.57	0.50
outdoor	GCW	0.81	0.39
outdoor	Scotoma	0.28	0.45
Overall		0.62	0.48

Table 32

Mean (SD) vividness ratings by stimulus type and condition.

stim_type	condition	mean_vividness	sd_vividness
abstract	FP	2.91	1.19
abstract	GCW	2.31	1.20
abstract	Scotoma	3.13	1.22
indoor	FP	4.90	1.28
indoor	GCW	4.17	1.18
indoor	Scotoma	4.83	1.26
outdoor	FP	4.68	1.29
outdoor	GCW	4.52	1.20
outdoor	Scotoma	4.52	1.31
Overall		3.99	1.53

Table 33

Factor	Mean
Object	3.25
Spatial	2.55
Verbal	3.04

Table 34

Factor	Mean
Object	3.14
Spatial	2.70
Verbal	3.11

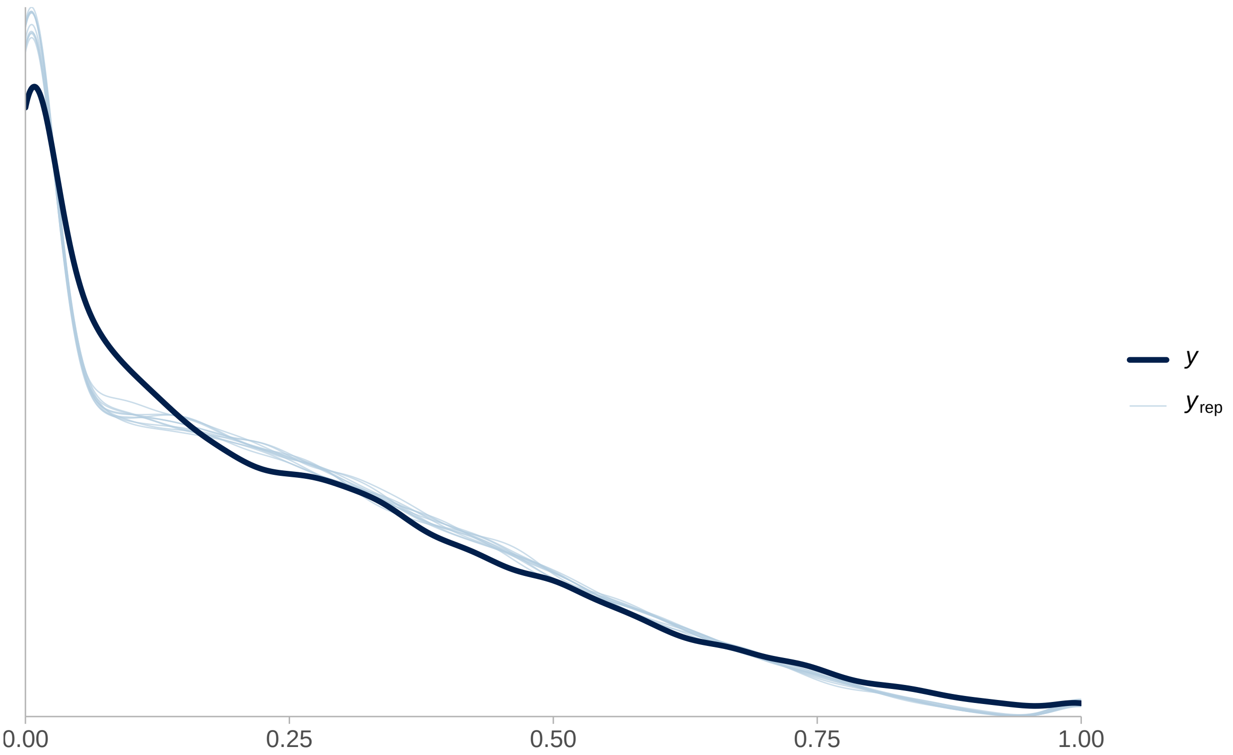

Figure 1

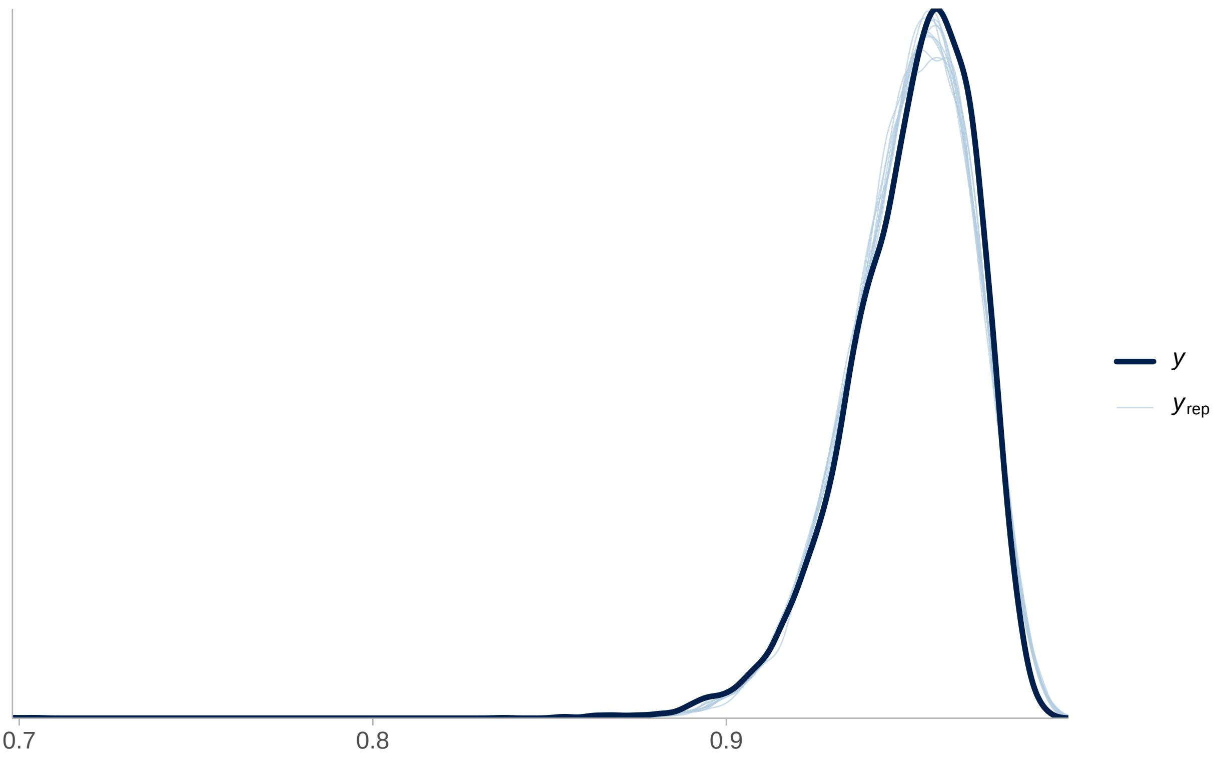

Figure 2

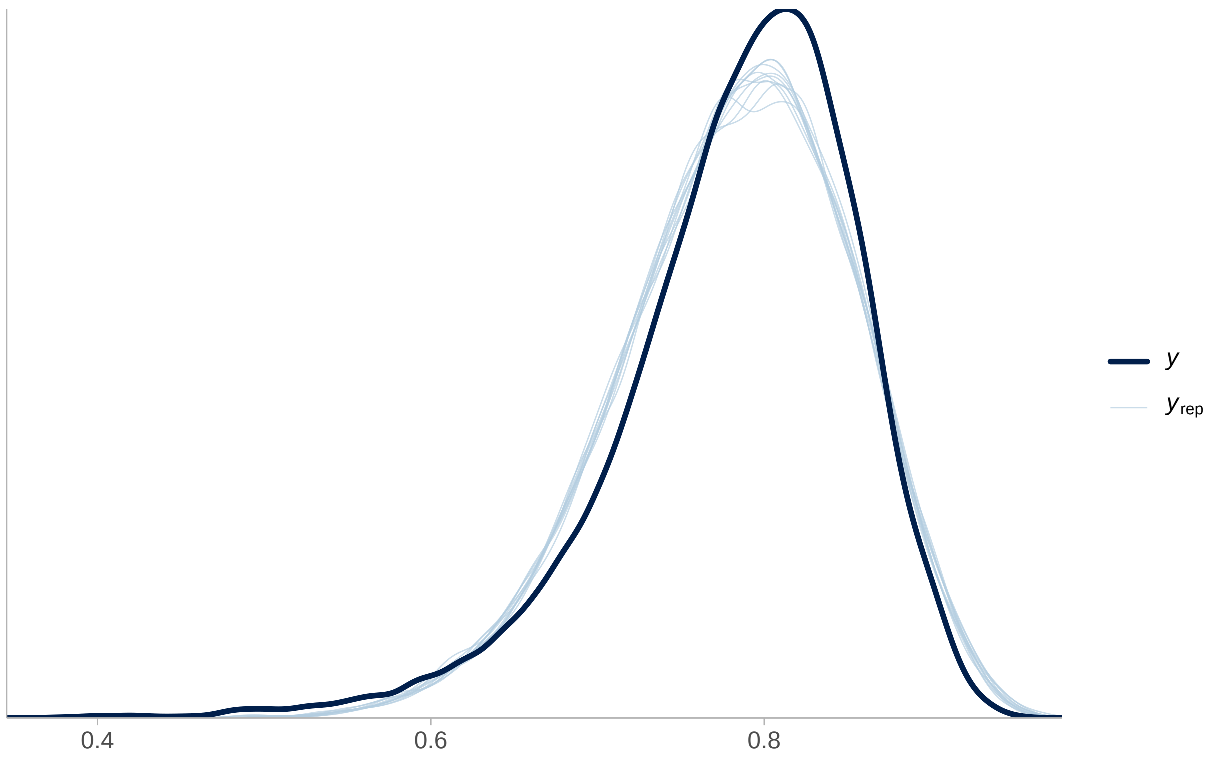

Figure 3

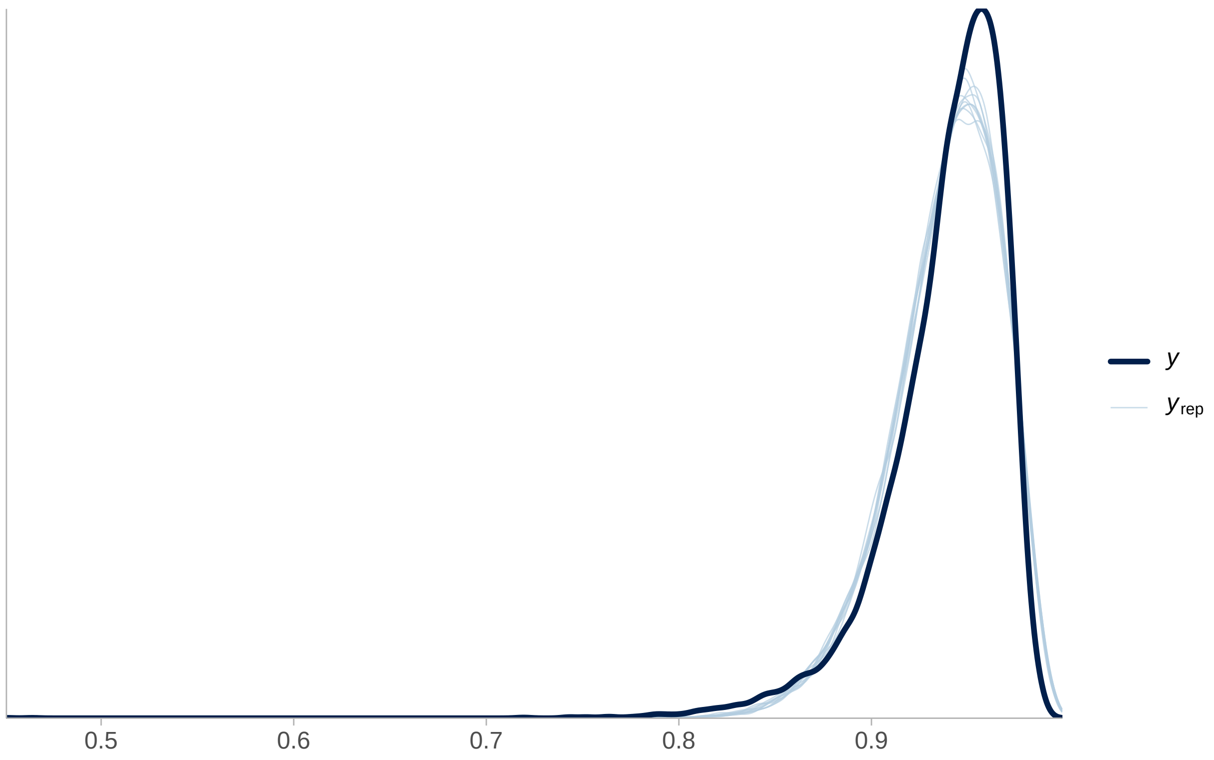

Figure 4

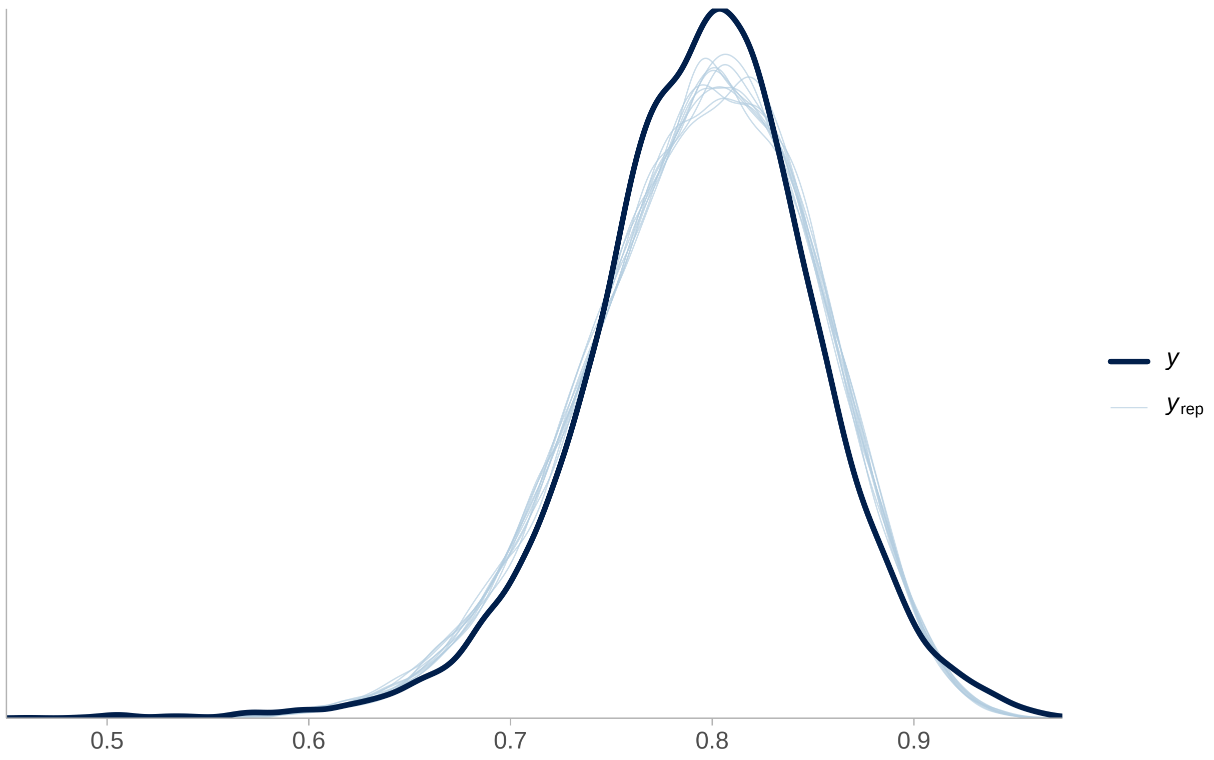

Figure 5

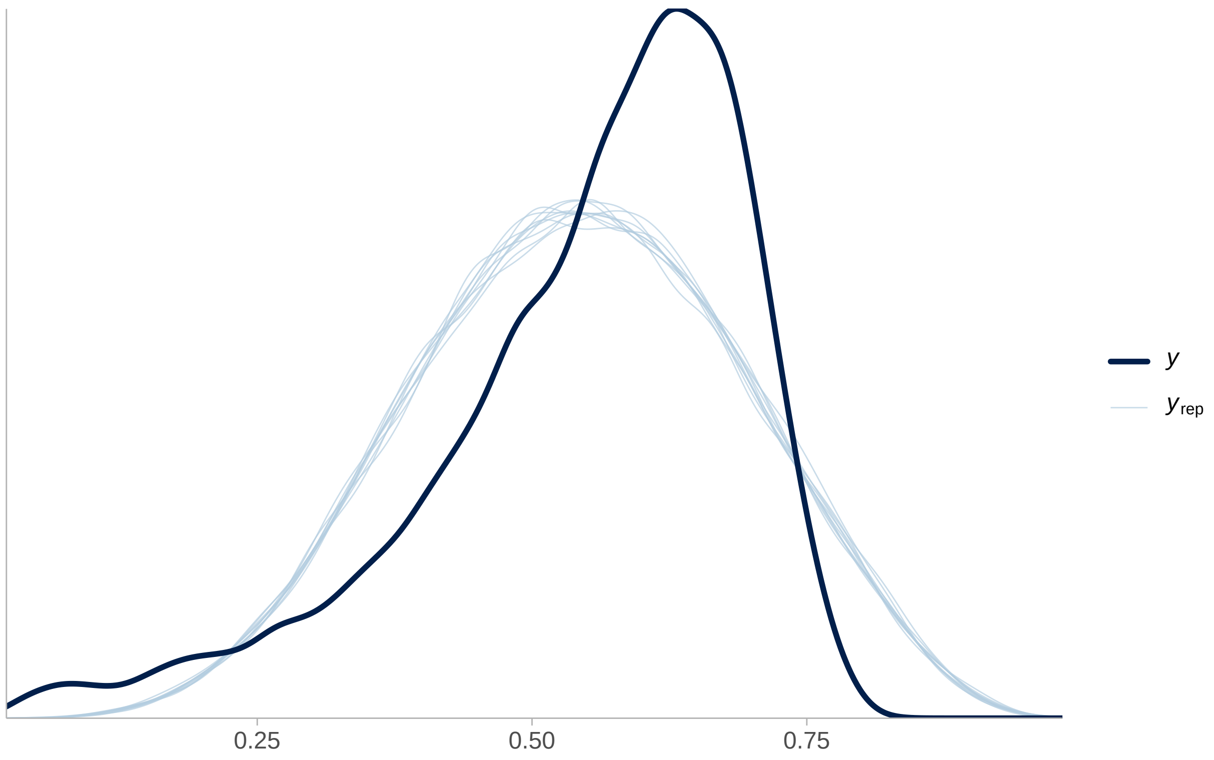

Figure 6

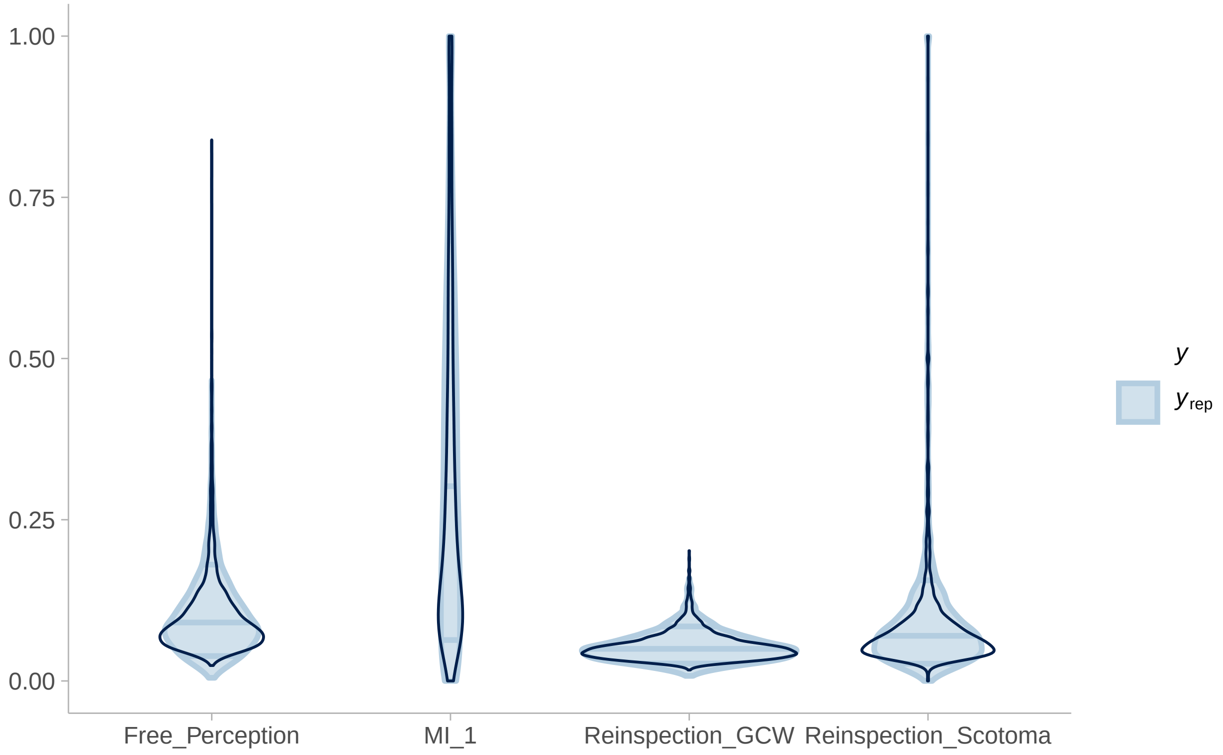
1.00
0.75
0.50
0.25
0.00
Free_Perception
MI_1
Reinspection_GCW
Reinspection_Scotoma
y
y_rep

Figure 7

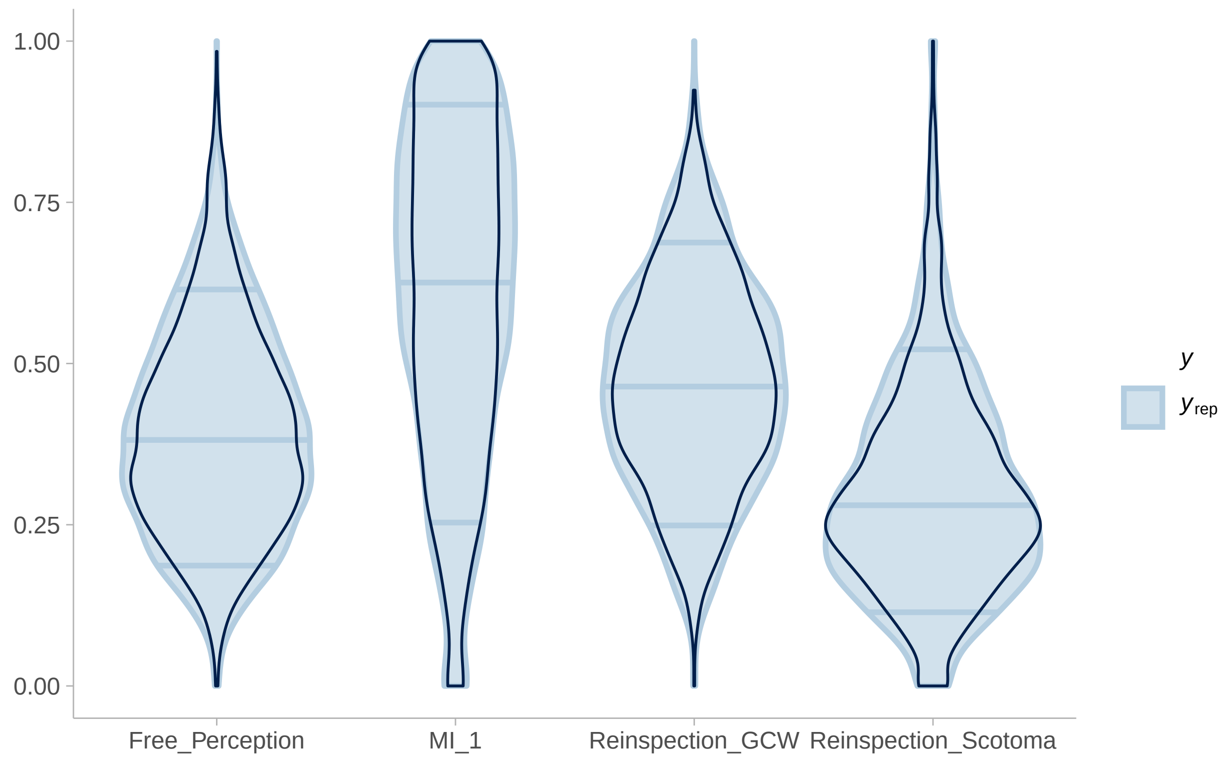
1.00
0.75
0.50
0.25
0.00
Free_Perception
MI_1
Reinspection_GCW
Reinspection_Scotoma
y
y rep

Figure 8

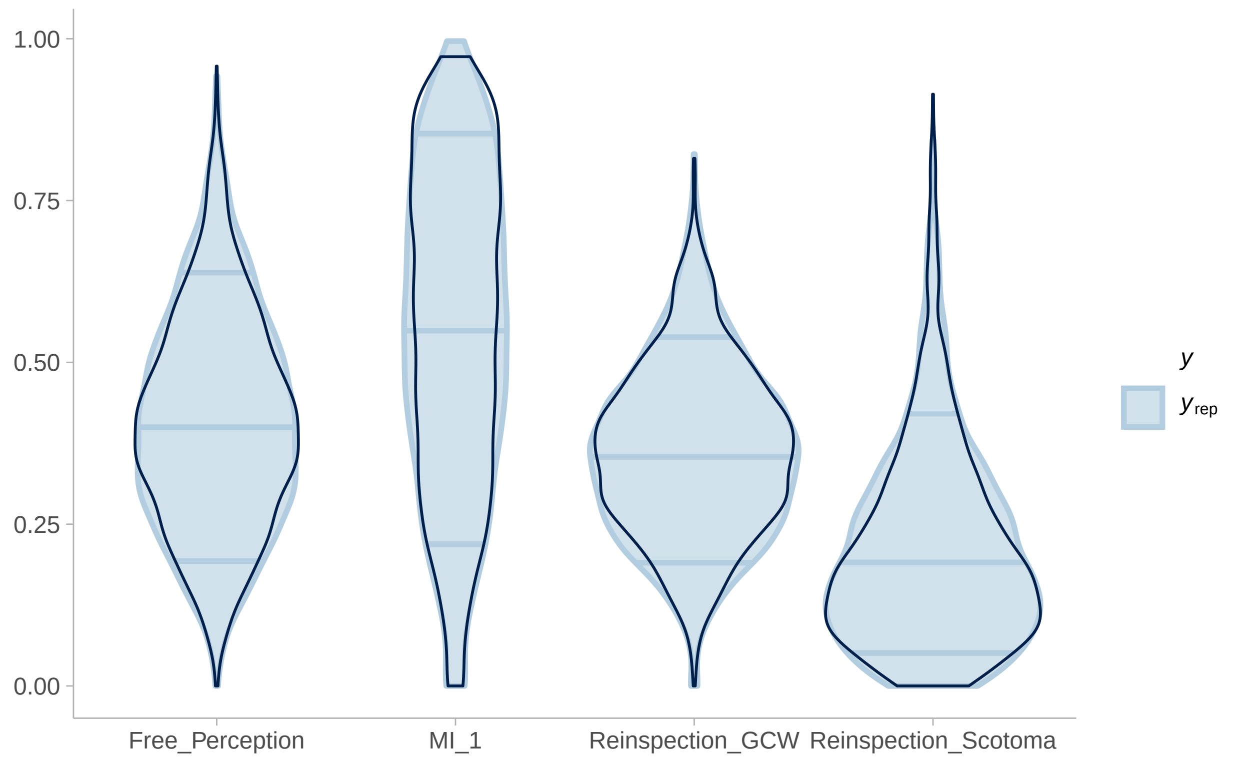
1.00
0.75
0.50
0.25
0.00
Free_Perception
MI_1
Reinspection_GCW
Reinspection_Scotoma
y
y_{rep}

Figure 9

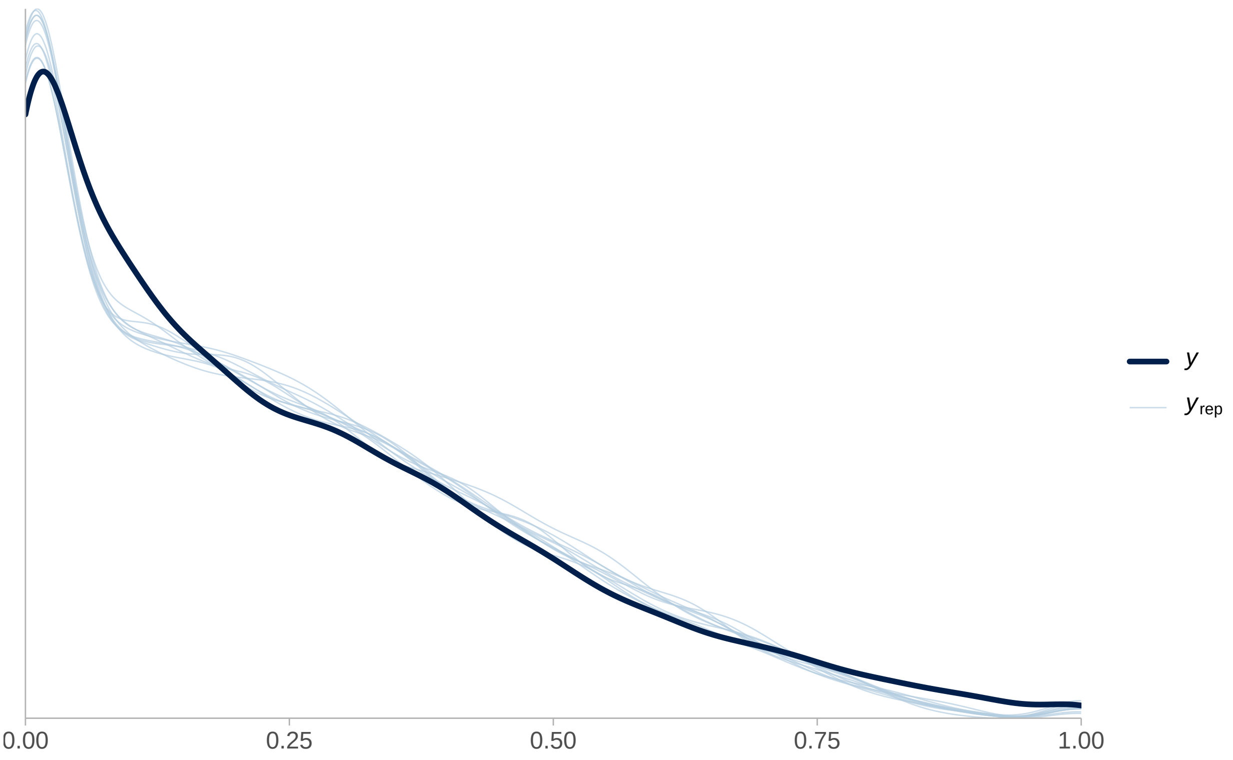

Figure 10

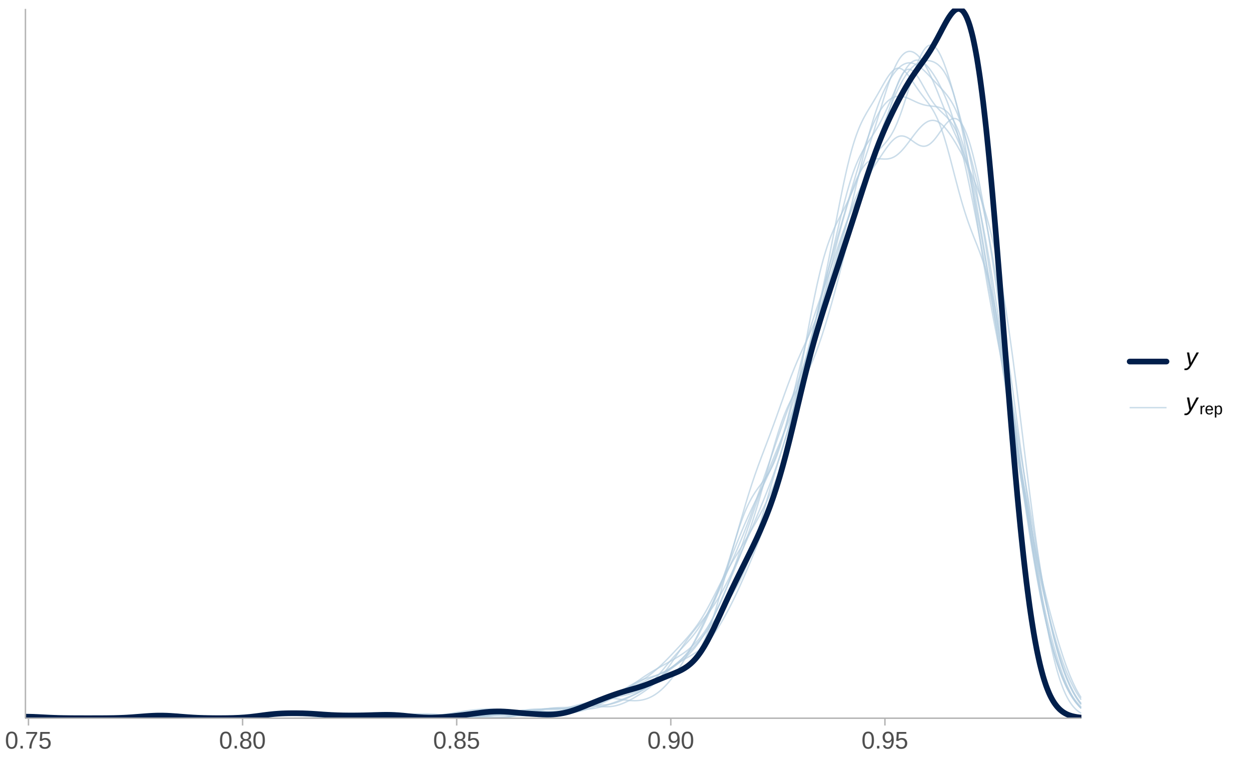

Figure 11

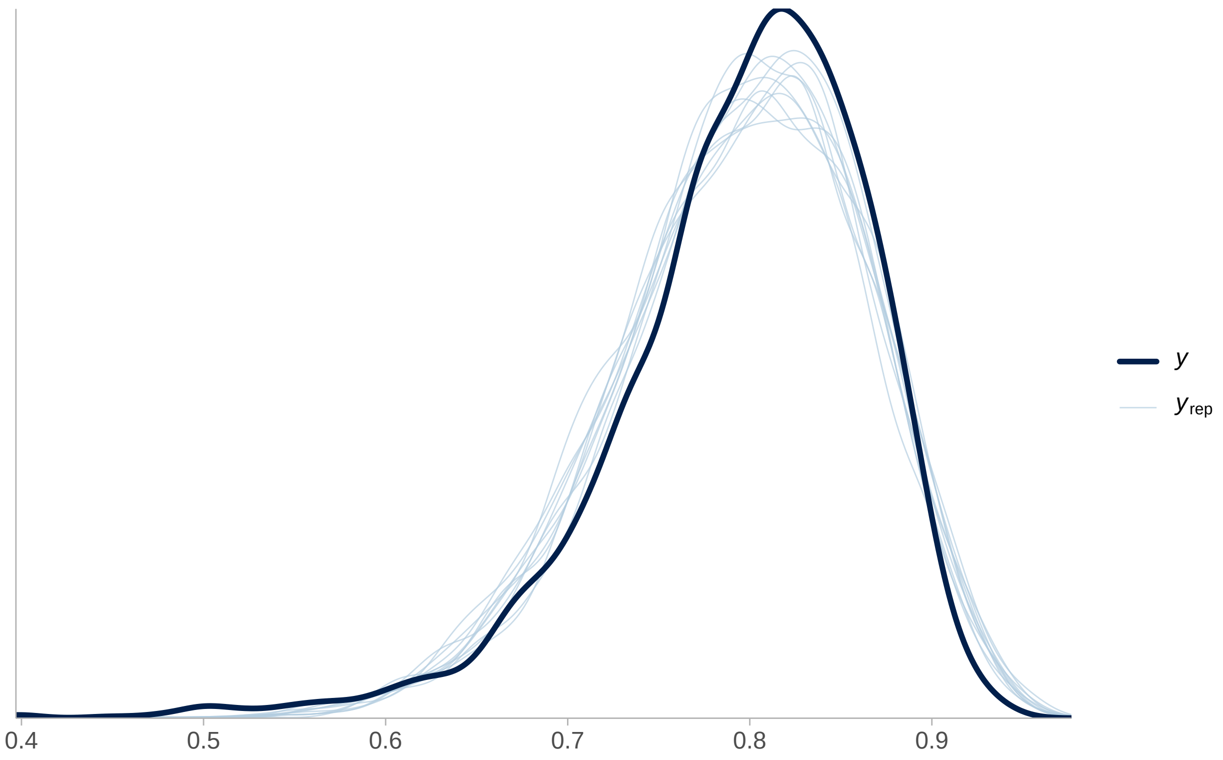

Figure 12

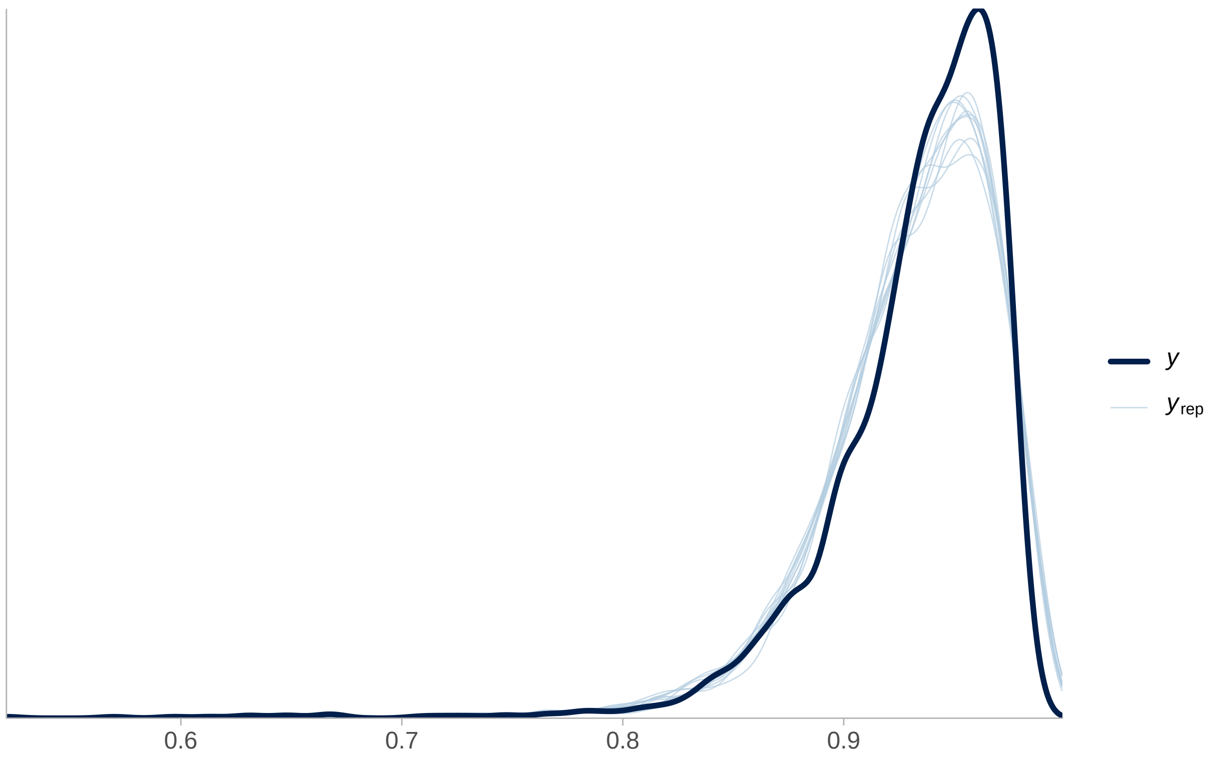

Figure 13

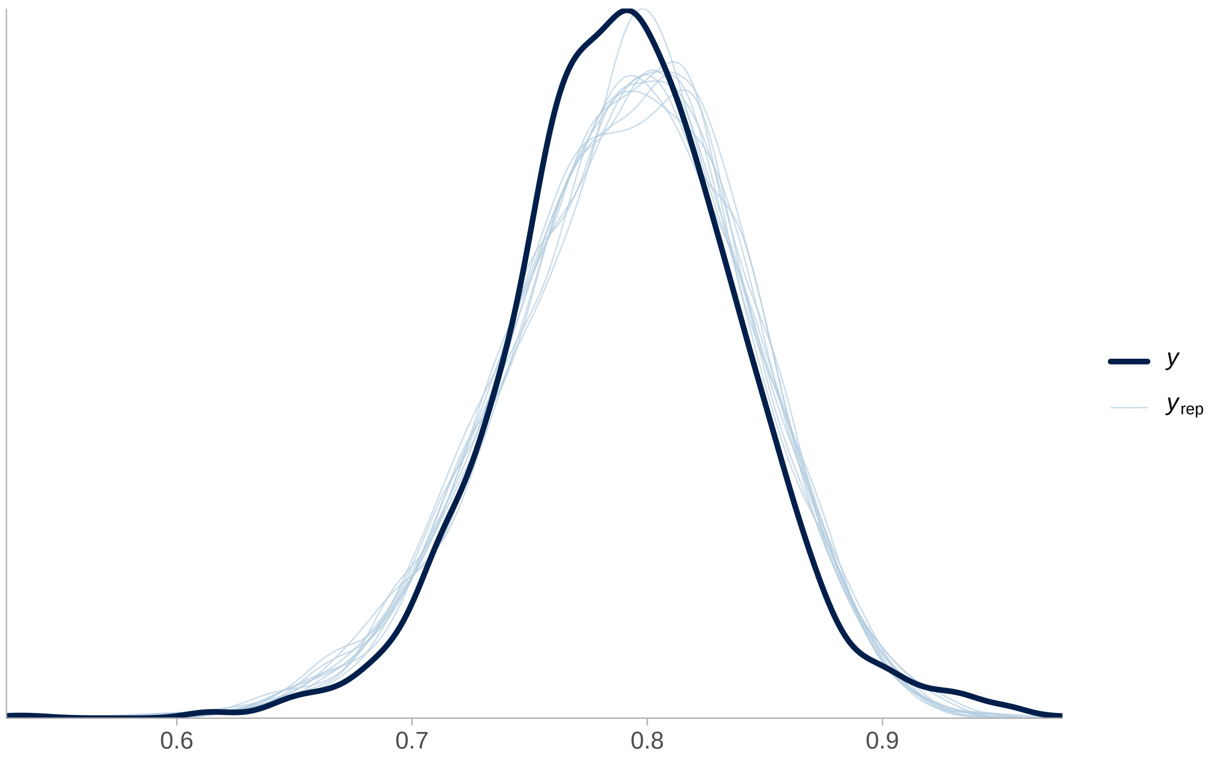

Figure 14

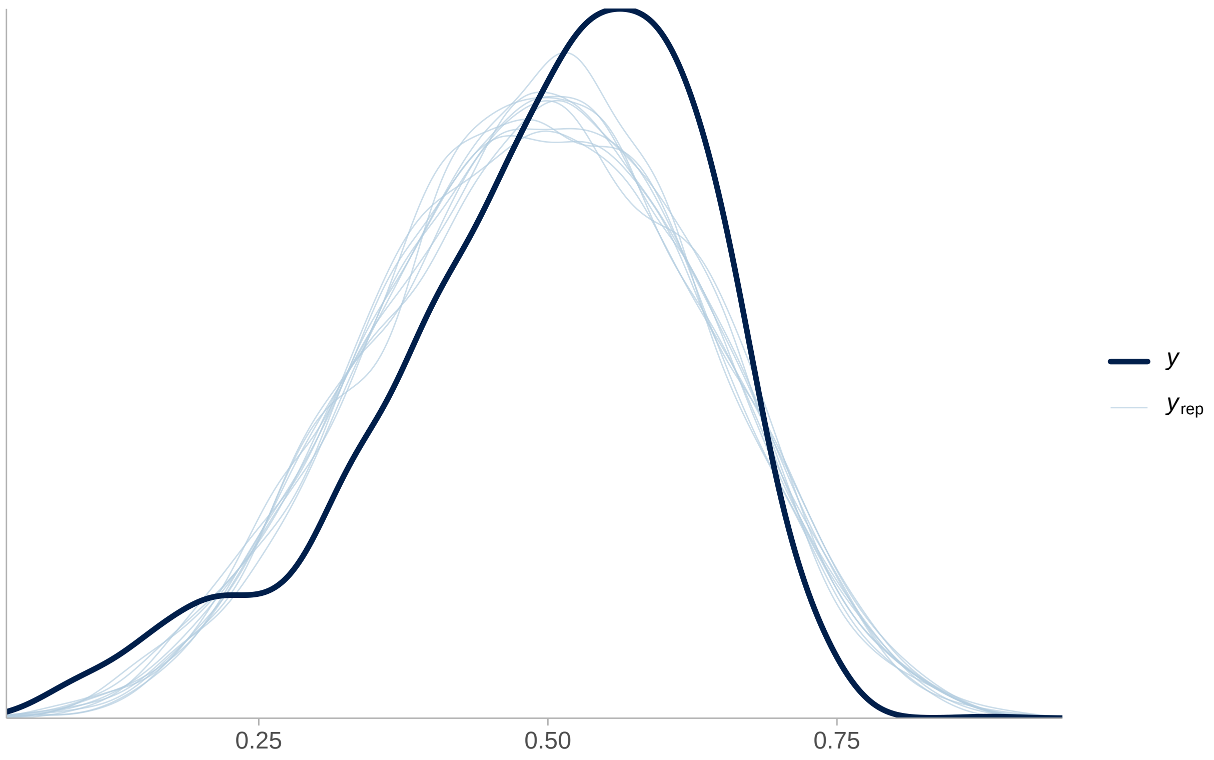

Figure 15

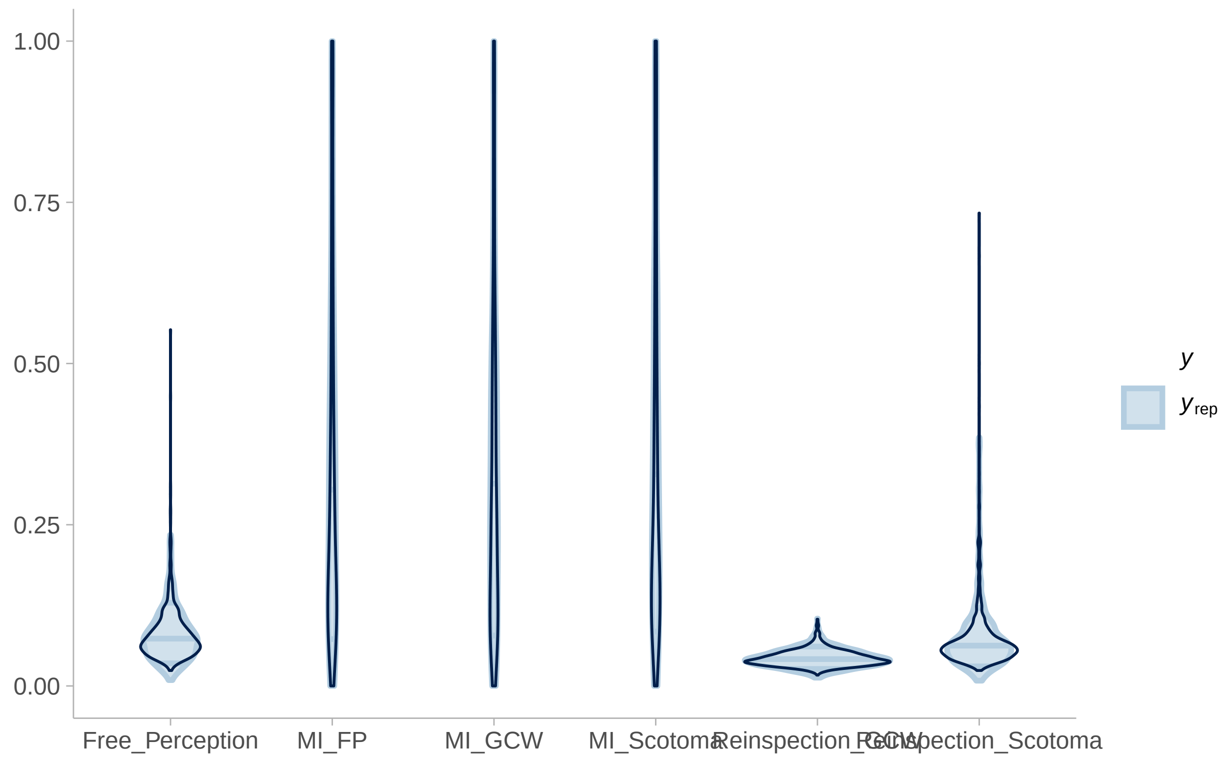
1.00
0.75
0.50
0.25
0.00
Free_Perception
MI_FP
MI_GCW
MI_Scotoma
Reinspection_GCW
Reinspection_Scotoma
y
y_{rep}

Figure 16

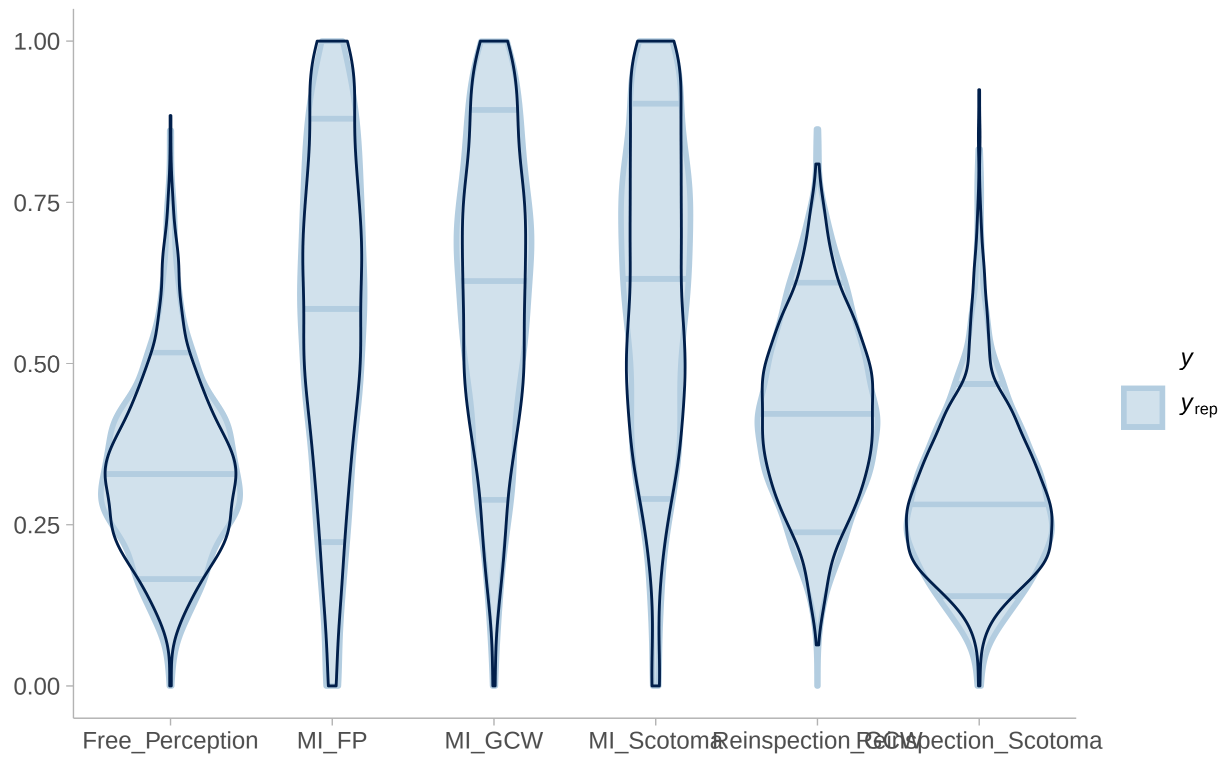
1.00
0.75
0.50
0.25
0.00
Free_Perception
MI_FP
MI_GCW
MI_Scotoma
Reinspection_GCW
Reinspection_Scotoma
y
y_{rep}

Figure 17

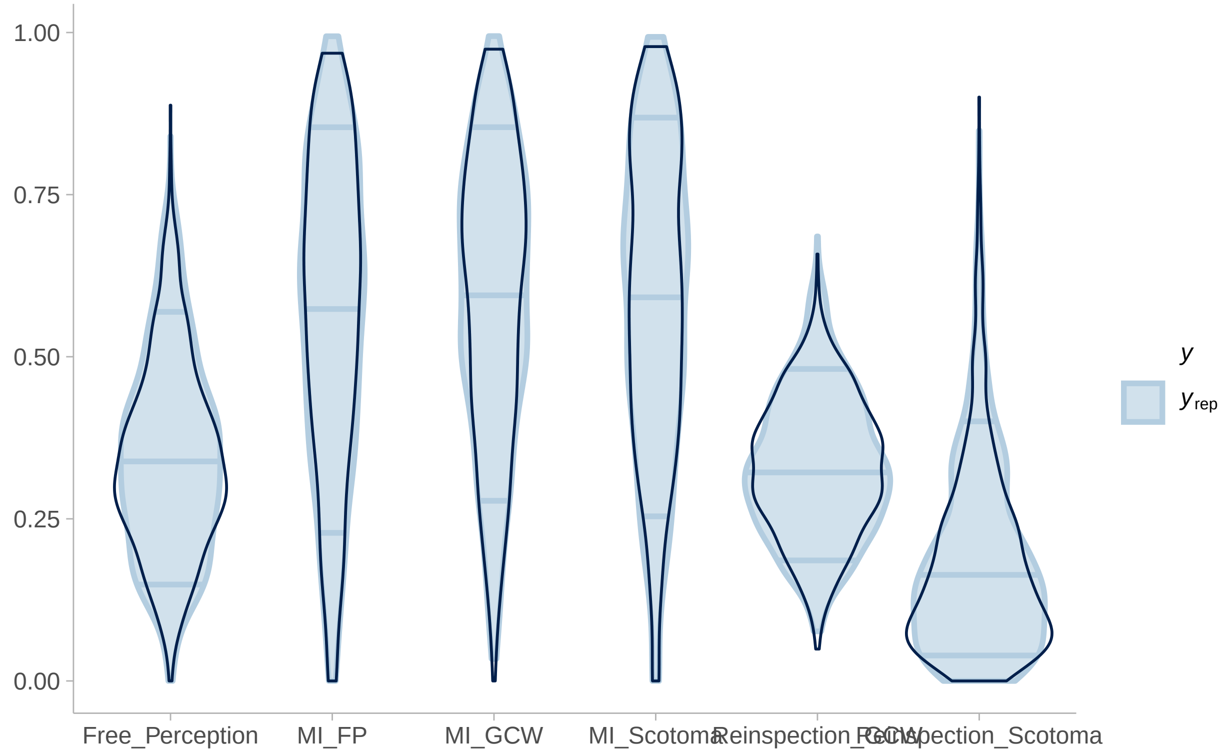
1.00
0.75
0.50
0.25
0.00
Free_Perception
MI_FP
MI_GCW
MI_Scotoma
Reinspection_GCW
Reinspection_Scotoma
y
y_{rep}

Figure 18

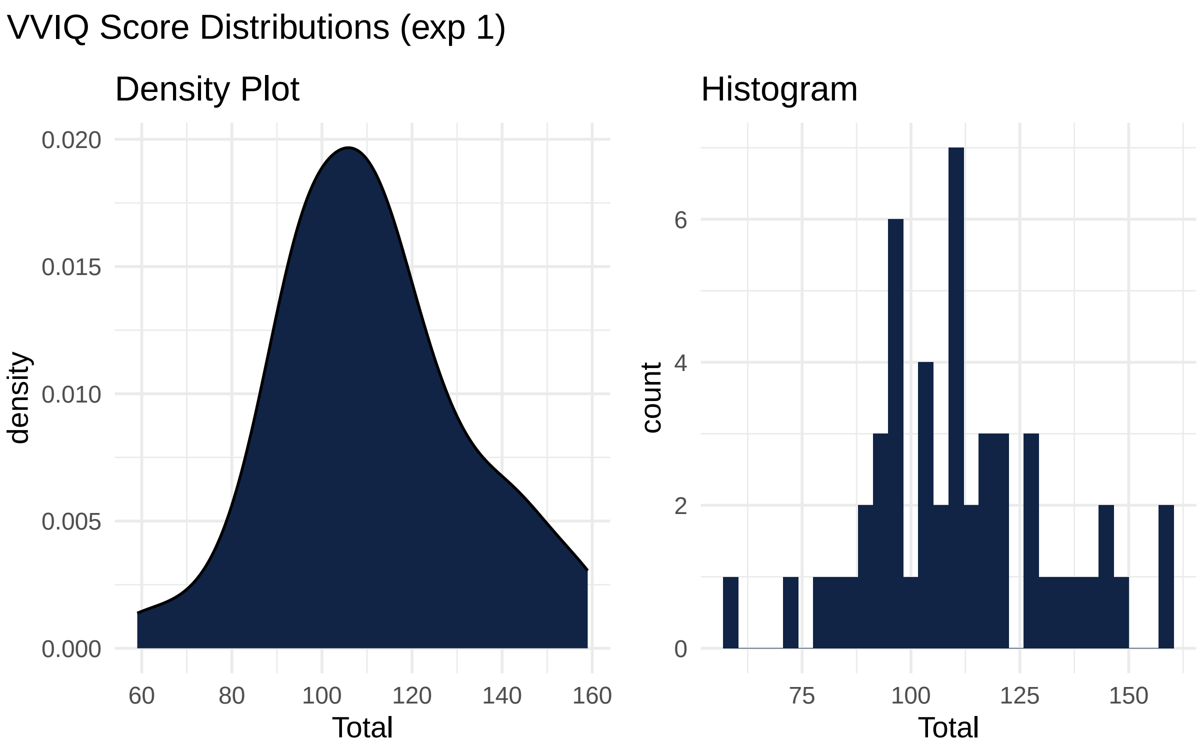
VVIQ Score Distributions (exp 1)
Density Plot
density
0.020
0.015
0.010
0.005
0.000
60
80
100
120
140
160
Total
Histogram
count
6
4
2
0
75
100
125
150
Total

Figure 19

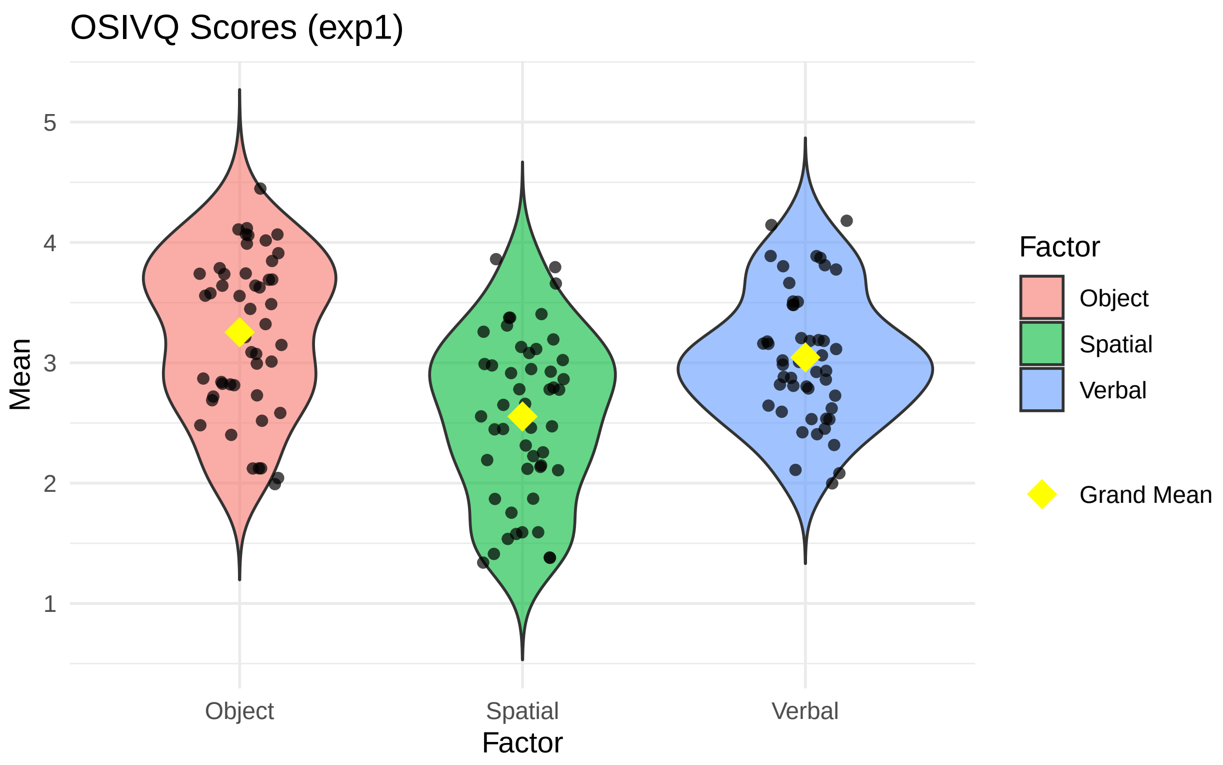
OSIVQ Scores (exp1)
Mean
5
4
3
2
1
Object
Spatial
Verbal
Factor
Factor
Object
Spatial
Verbal
Grand Mean

Figure 20

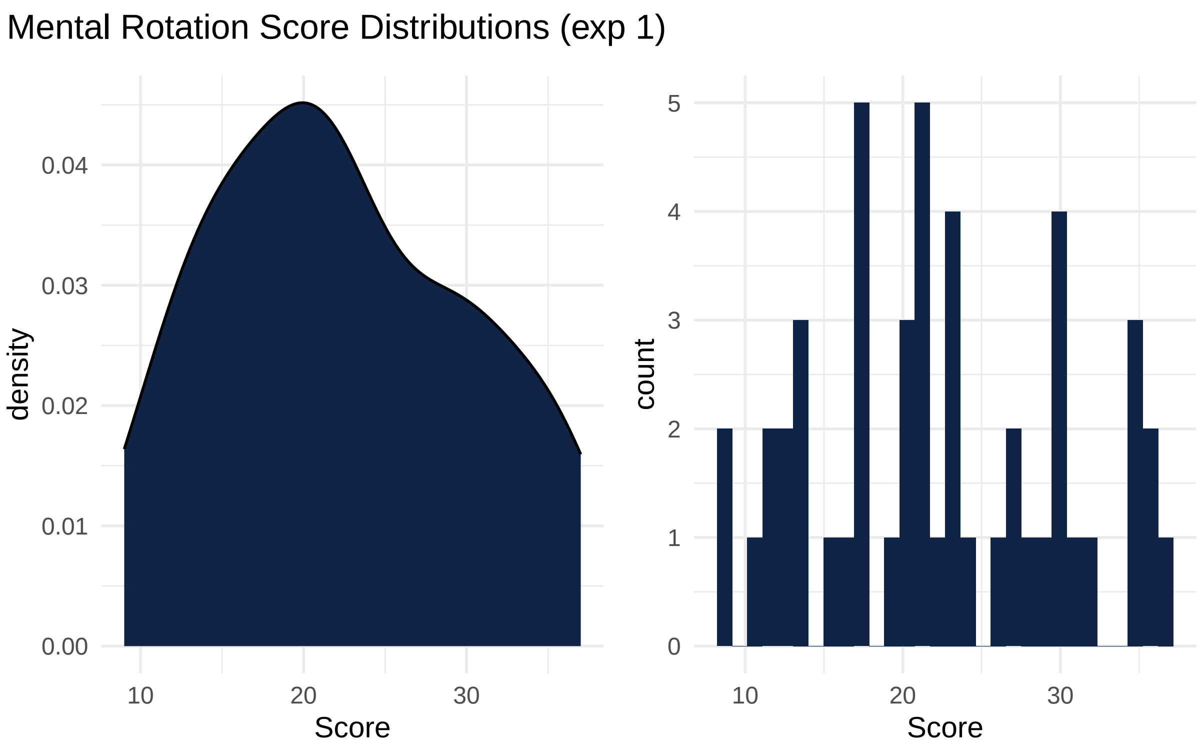
Mental Rotation Score Distributions (exp 1)
density
0.04
0.03
0.02
0.01
0.00
10
20
30
Score
count
5
4
3
2
1
0
10
20
30
Score

Figure 21

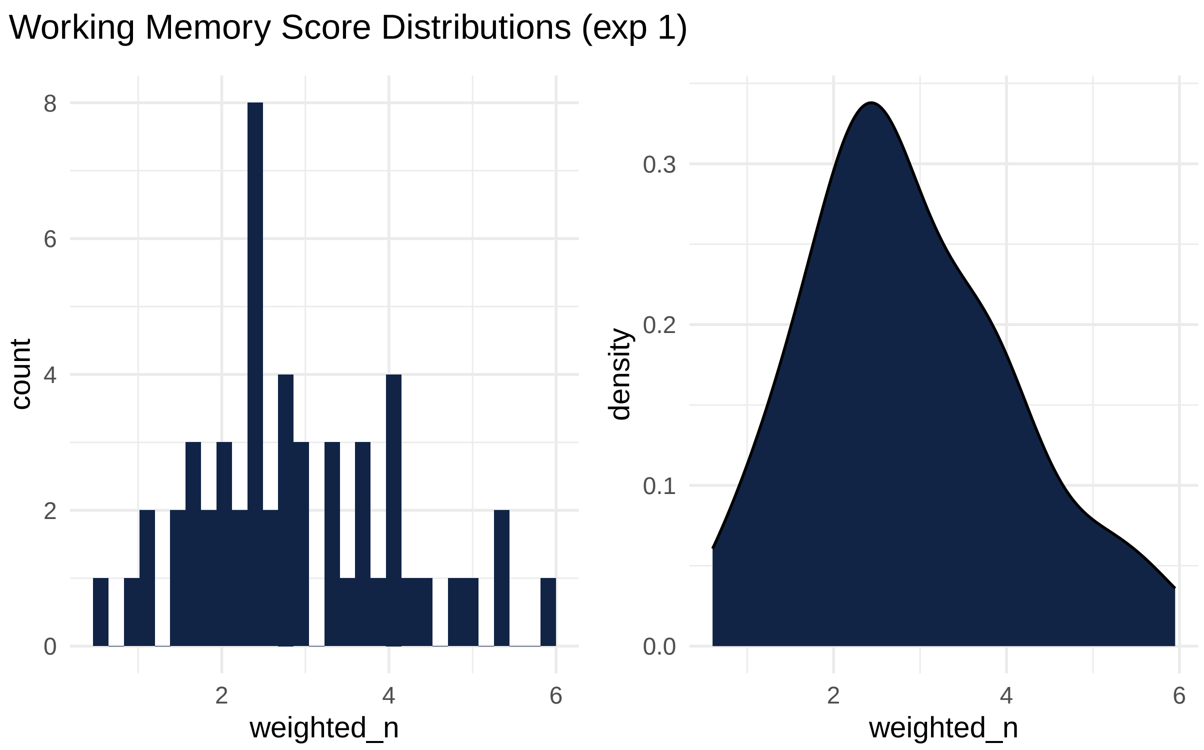
Working Memory Score Distributions (exp 1)
count
8
6
4
2
0
2
4
6
weighted_n
density
0.3
0.2
0.1
0.0
2
4
6
weighted_n

Figure 22

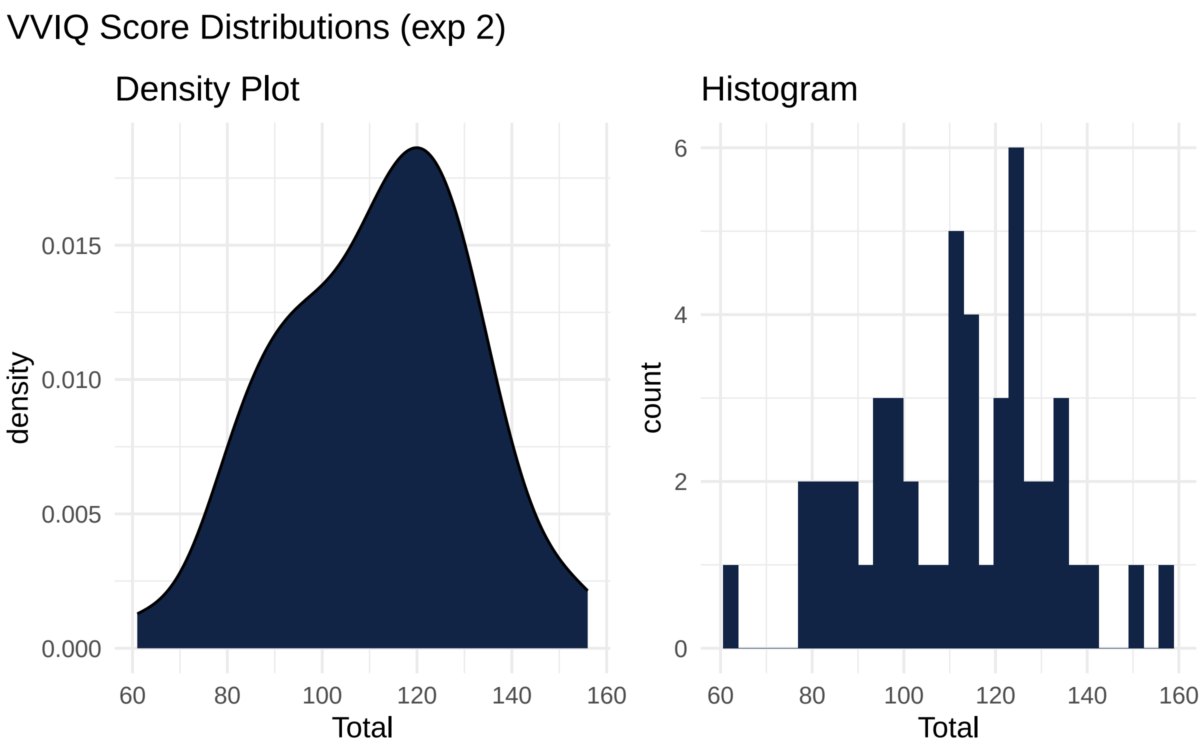
VVIQ Score Distributions (exp 2)
Density Plot
density
0.000
0.005
0.010
0.015
60
80
100
120
140
160
Total
Histogram
count
0
2
4
6
60
80
100
120
140
160
Total

Figure 23

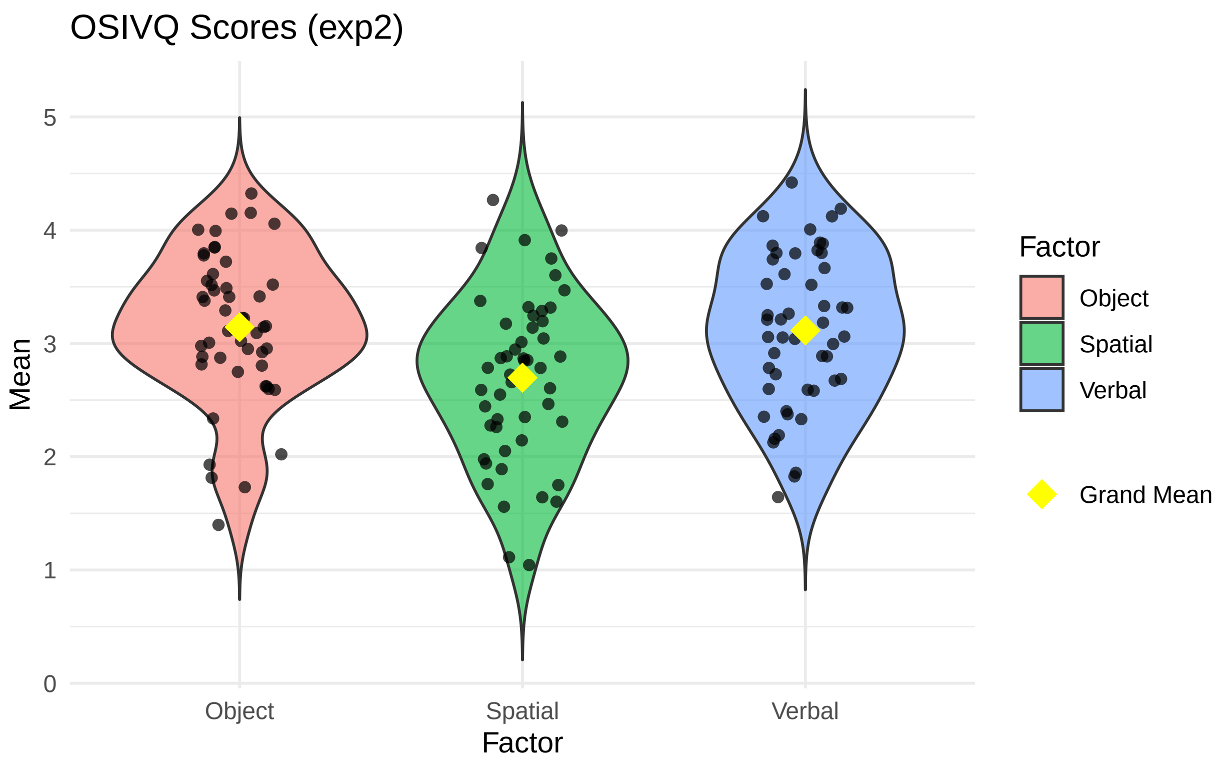
OSIVQ Scores (exp2)
Mean
5
4
3
2
1
0
Object
Spatial
Verbal
Factor
Factor
Object
Spatial
Verbal
Grand Mean

Figure 24

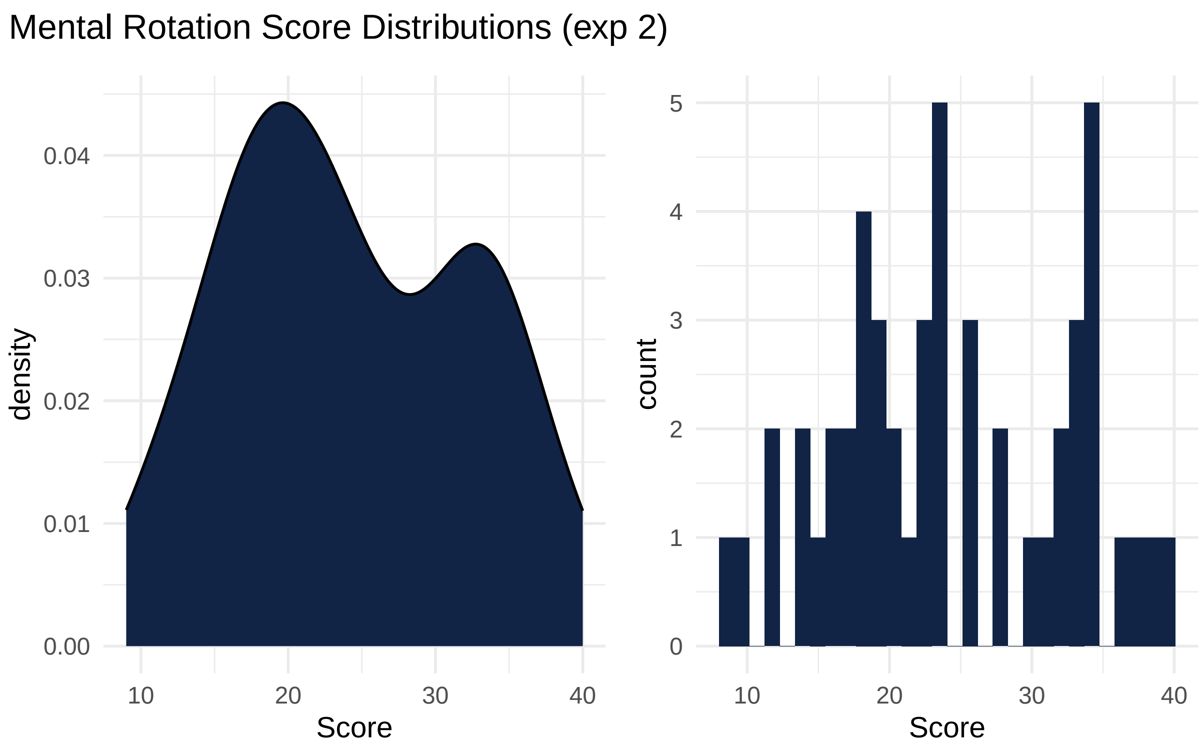
Mental Rotation Score Distributions (exp 2)
density
0.04
0.03
0.02
0.01
0.00
10
20
30
40
Score
count
5
4
3
2
1
0
10
20
30
40
Score

Figure 25

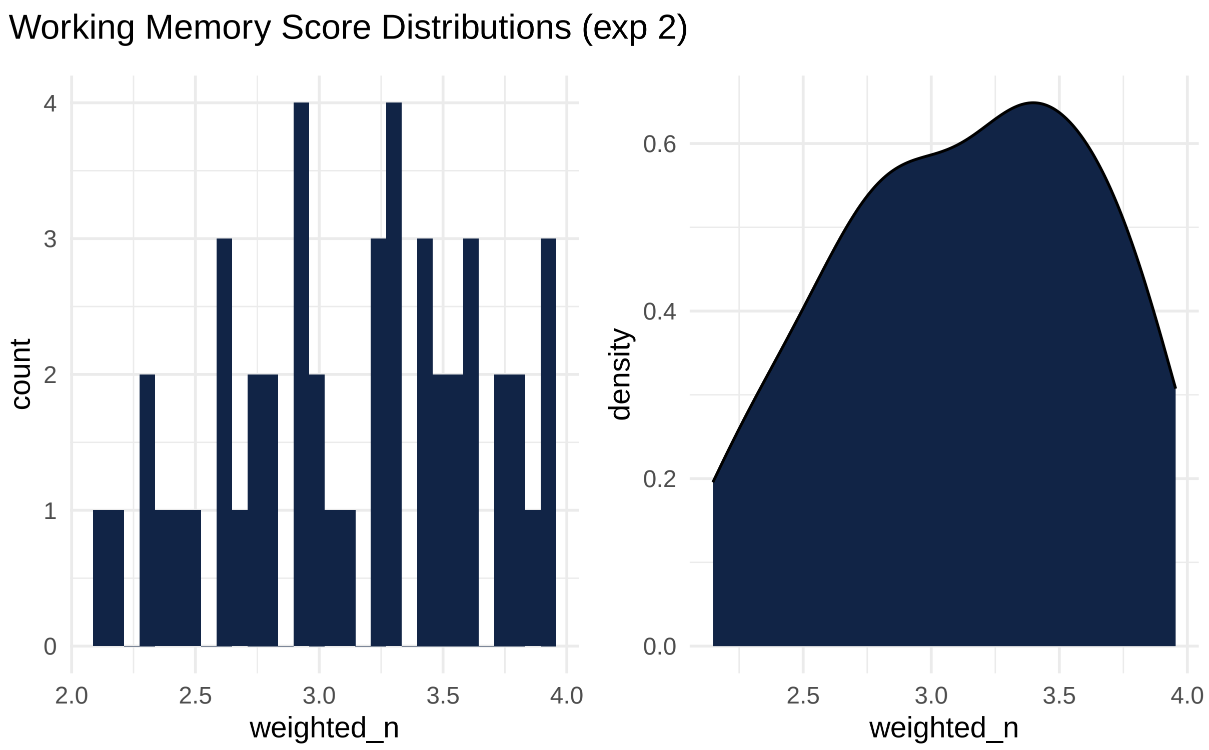
Working Memory Score Distributions (exp 2)
count
weighted_n
density
weighted_n
0
1
2
3
4
2.0
2.5
3.0
3.5
4.0
0.0
0.2
0.4
0.6
2.5
3.0
3.5
4.0

Figure 26
